# Mechanistic Studies on the Synthesis of Pyrrolidines and Piperidines via Copper-Catalyzed Intramolecular C–H Amination

**DOI:** 10.1021/acs.organomet.2c00095

**Published:** 2022-04-29

**Authors:** José María Muñoz-Molina, Daniel Bafaluy, Ignacio Funes-Ardoiz, Adiran de Aguirre, Feliu Maseras, Tomás R. Belderrain, Pedro J. Pérez, Kilian Muñiz

**Affiliations:** † Laboratorio de Catálisis Homogénea, Unidad Asociada al CSIC, CIQSO-Centro de Investigación en Química Sostenible and Departamento de Química, Universidad de Huelva, 21007 Huelva, Spain; ‡ Institute of Chemical Research of Catalonia, ICIQ, The Barcelona Institute of Science and Technology, Av. Països Catalans, 16, 43007 Tarragona, Spain; § Departament de Química, Universitat Autònoma de Barcelona, 08193 Bellaterra, Spain

## Abstract

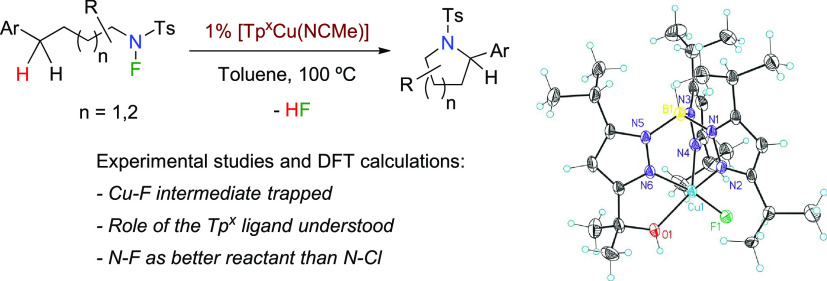

We have recently developed a method for the synthesis of pyrrolidines and piperidines via intramolecular C–H amination of *N*-fluoride amides using [Tp^*x*^CuL] complexes as precatalysts [Tp^*x*^ = tris(pyrazolyl)borate ligand and L = THF or CH_3_CN]. Herein, we report mechanistic studies on this transformation, which includes the isolation and structural characterization of a fluorinated copper(II) complex, [(Tp^iPr2^OH)CuF] [Tp^iPr^ = hydrotris(3,5-diisopropylpyrazolyl)borate], pertinent to the mechanistic pathway. The effects of the nature of the Tp^*x*^ ligand in the copper catalyst as well as of the halide in the N–X amides employed as reactants have been investigated both from experimental and computational perspectives.

## Introduction

**Scheme 1:**
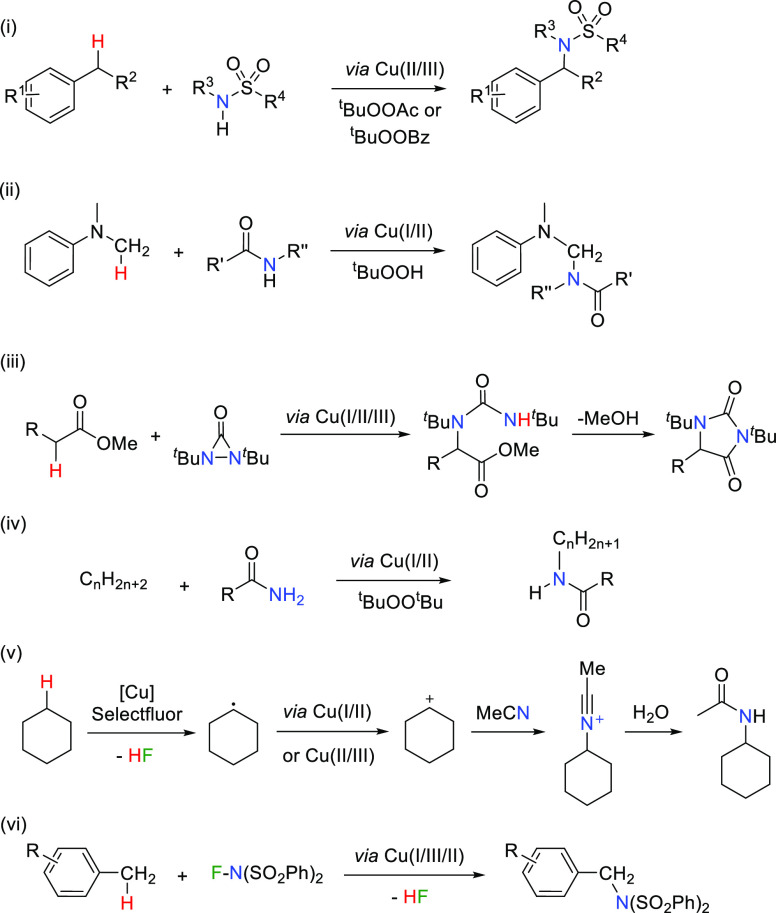
Copper-Catalyzed C–H Amination Processes

Amination reactions have become powerful methods to synthesize natural, organic, and medicinally important compounds. In this context, the direct amination of C(sp^3^)–H bonds constitutes one of the most attractive routes to prepare C(sp^3^)–N bonds, providing chemo-, regio-, and stereoselectivity.^1^ Transition metals can catalyze innovative C–H amination processes, and among them, the first row d-block elements have become an alternative to precious late transition metals, allowing the exploration of unprecedented catalytic transformations.^1^ Particularly, copper complexes exhibit unique and versatile reactivity with good functional group tolerance toward that end.^2^ Several C(sp^3^)–H bond amination processes catalyzed by copper have been described to date (Scheme 1);^2a^ the proposed mechanisms usually involve oxidation states of copper complexes from Cu(I) to Cu(III), either in two-electron or single-electron processes or even with both steps in the same catalytic cycle. Although the state-of-the-art of these systems is dominated by nitrene chemistry, over the last two decades, a number of radical-based functionalization systems have been successfully developed: (i) amidation of allylic and benzylic C–H bonds with sulfonamides using *tert*-butyl peracetate (^*t*^BuOOAc) or *tert*-butyl perbenzoate (^*t*^BuOOBz) as oxidants;^3^ (ii) amidation of inactivated C(sp^3^)–H bonds adjacent to a nitrogen atom using *tert*-butyl hydroperoxide (^*t*^BuOOH) as the oxidant;^4^ (iii) α-amination of esters using di-*tert*-butyldiaziridinone;^5^ (iv) amidation of alkanes, including light alkanes, with di-tertbutylperoxide and amides;^6^ (v) intermolecular C–H amination via the generation of highly reactive radical species from Selectfluor;^7^ and (vi) benzylic C–H bond amination using *N*-fluorobenzenesulfonimide (NFSI).^8^ Some of the proposed mechanisms for these systems still remain unclear as is the case of the latter process. Itami and Musaev showed that the reaction between NFSI and bipyridine-supported CuBr is more complicated than a simple bimolecular one- or two-electron oxidative addition.^9^

We have recently communicated^10^ a method for the synthesis of both pyrrolidines and piperidines via intramolecular C–H amination of *N*-fluoride amides (eq 1) using complex [Tp^iPr2^Cu(NCMe)] as a well-defined precatalyst [Tp^iPr^ = hydrotris(3,5-diisopropyl-1-pyrazolyl)borate]. At variance with other previous methods for catalytic copper activation of N–F bonds in C–H functionalization, which addressed C–C bond formation, this system induces the corresponding amination. Moreover, the application to the synthesis of piperidines is also noteworthy since most reported systems only provide pyrrolidines. A catalytic cycle through a Cu(I)/Cu(II) pathway was proposed on the basis of experimental and density functional theory (DFT) investigations. Herein, we present a complementary mechanistic study including the influence of the nature of the Tp^*x*^ ligand in the reaction outcome, the isolation of relevant fluorinated copper intermediates, and the effect of the halide in the reactant, among other variables, to complete the previously proposed mechanistic picture.
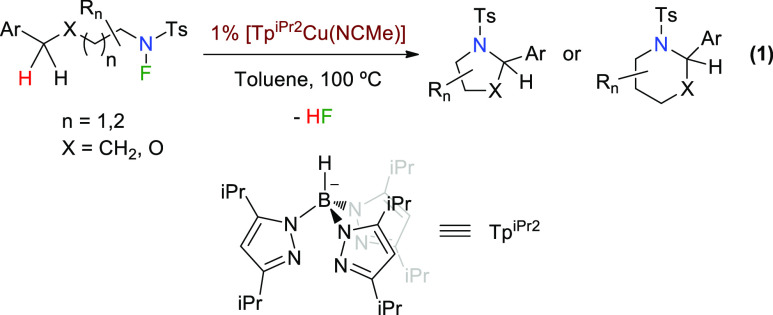
1

## Results and Discussion

### Copper Fluoride Intermediates

**Scheme 2:**
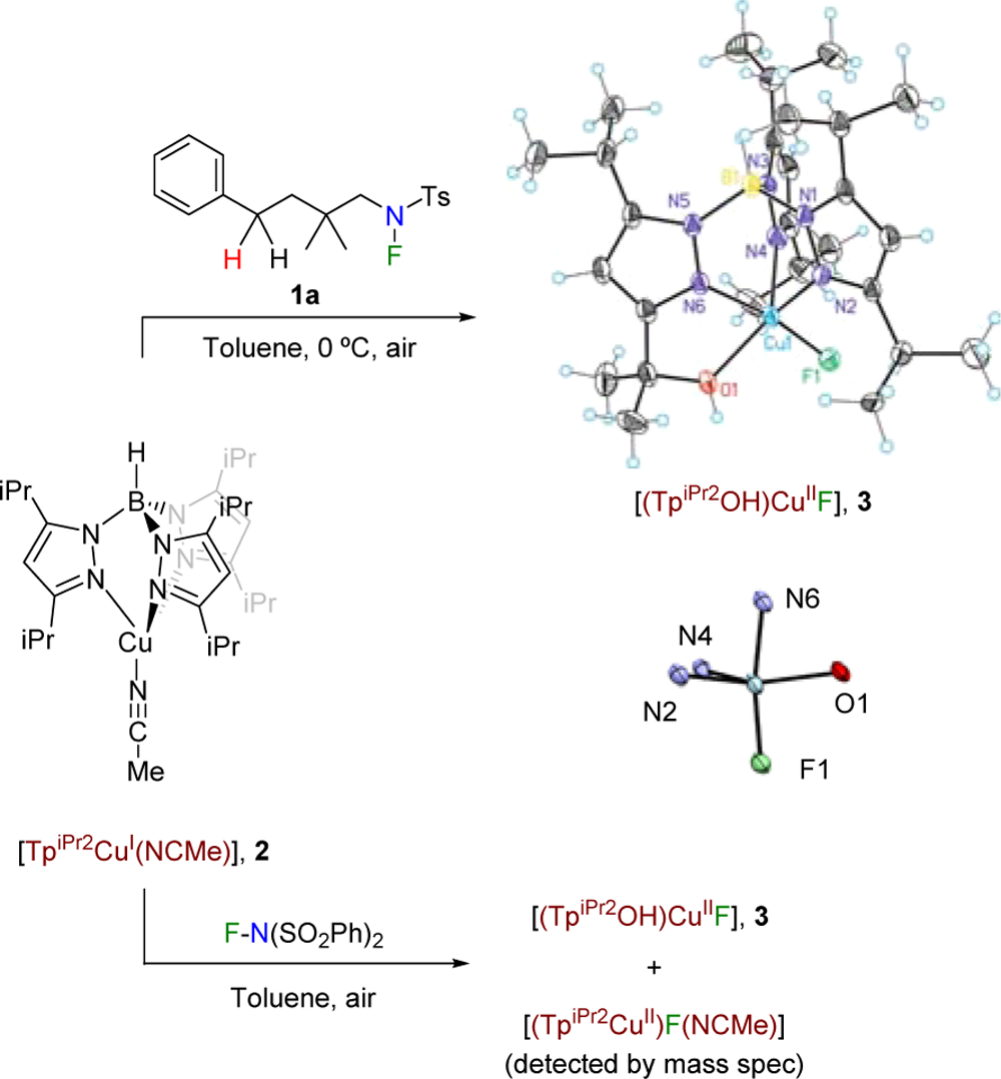
Observation of Cu–F-Containing Complexes

We started these investigations considering the possible participation of [Tp^*x*^Cu^II^F] species in the catalytic cycle responsible for the transformation shown in eq 1. Our previous work^10^ demonstrated that the electron paramagnetic resonance spectra of a mixture of **1a** (Scheme 2) and [Tp^iPr2^Cu(NCMe)] (**2**) showed the formation of a new copper(II) species, presumably as the result of the homolytic cleavage of the N–F bond. Unfortunately, efforts to isolate any copper complex from this reaction mixture were unsuccessful.

At variance with this, when the reaction mixture was exposed to air, crystalline material of a new complex was obtained, being structurally characterized as [(Tp^iPr2^-OH)CuF] (**3**, Scheme 2).^11^ This compound results from the aerobic oxidation of the tertiary C–H bond of one iPr group at the trispyrazolylborate ligand. A recent work from one of our laboratories has disclosed a similar oxidation reaction of a C(sp^3^)–H bond of a different Tp^*x*^ ligand, leading to trinuclear complexes.^12a^ Complex **3** has been independently synthetized from the direct reaction of Tp^iPr2^Cu(NCMe) and NFSI. Mass spectroscopy studies carried out with the latter reaction mixture showed the presence of a species of composition [Tp^iPr2^CuF(NCMe)] as a probable intermediate en route to complex **3**. We interpret these results as an indication of the generation of a Cu(II)–F bond from the interaction of the initial Cu(I) complex and the fluorinated substrate. Isolated complex **3** was tested as a catalyst for the cyclization reaction using **1a** as the substrate, showing a catalytic activity similar to that of other Cu(II) catalyst precursors described in our previous work but lower than that achieved with the parent Tp^iPr2^Cu(NCMe) catalyst. Moreover, kinetic studies also showed that the reaction rates with the latter are faster than that with complex **3** (see the Supporting Information); therefore, this is not an intermediate in the transformation studied.

Complex **3** has been structurally characterized by X-ray diffraction studies. The geometry around the copper center is trigonal bipyramidal (Scheme 2, inset), with a Cu–O distance of 2.086 Å, which is slightly larger than those reported for similar oxidation processes, affording trinuclear compounds [1.919 Å for Tp^Ms^_2_(O_2_)_3_Cu_3_^12a^ or 2.001 Å for Cu_3_(Br)(L1O)_3_(PF_6_)_2_)],^12b^ where the oxygen is bonded to two copper ions. The Cu–N distances are within the 1.90–2.13 Å interval, being similar to that of other Cu(II)-containing Tp^*x*^ ligands.^12a^

### Kinetic Isotope Effect Experiments

The use of deuterium-labeled substrates has provided valuable information. We had previously obtained the individual reaction rates for *N*-fluoro-sulfonamides **1b** and **1b–d2** with a fully deuterated benzylic position, showing a kinetic isotope effect (KIE) value of 1.7, consistent with the C–H bond cleavage as the turnover-limiting step. We have now obtained additional information from equations 2 and 3. Thus, when the monodeuterated *N*-fluoro-sulfonamide **1b–d1** was employed, a primary KIE *k*_H_/*k*_D_ = 3.3 was measured in a direct competition experiment (eq 2) using the Tp^iPr2^-containing catalyst. A similar experiment leading to piperidine formation via six-membered cyclization gave a *k*_H_/*k*_D_ value of 4.2 (see the Supporting Information). Furthermore, the corresponding intermolecular competition reaction between **1b** and **1b–d2** was also carried out and gave a *k*_H_/*k*_D_ value of 1.4 (eq 2). The related experiment performed with starting materials leading to piperidine products provided the same *k*_H_/*k*_D_ value of 1.4 (see the Supporting Information). These KIE values support, with no doubt, the C–H bond cleavage as the turnover-limiting step.^13^
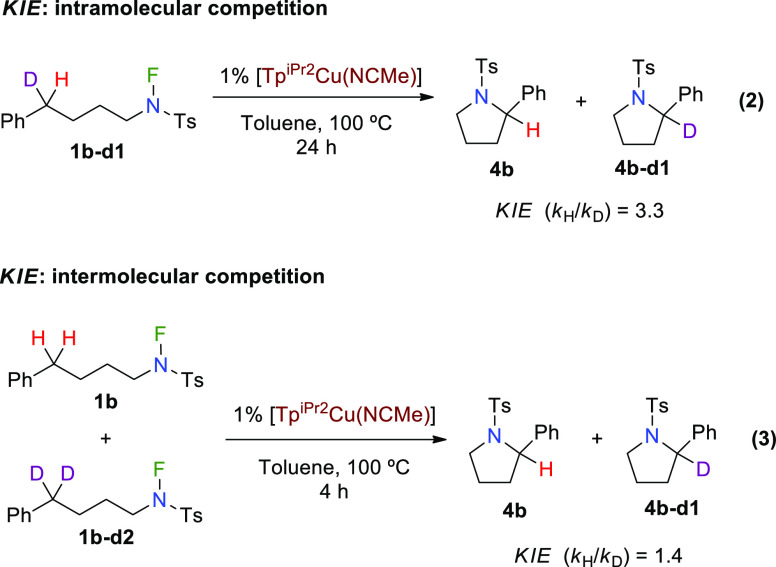
2

### Effect of the Tp^*x*^ Ligand

The availability of an array of R groups that can be installed in the pyrazolyl rings of Tp^*x*^ ligands allows the control of the steric and electronic properties of the metal complex. In our previous report, we observed a subtle steric influence of the ligand in the diastereoselectivity of product **4c** (eq 4). As a possible explanation for this behavior, we speculate that the steric hindrance exerted by the complex in the transition state of the cyclization step is not enough to greatly impact the reaction outcome.
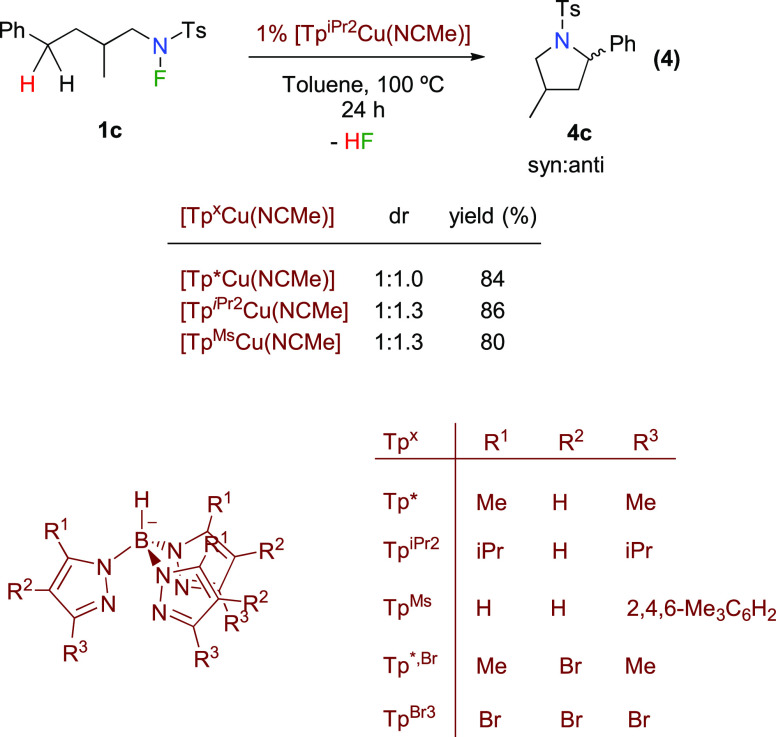
4

The effect of the ligand has now been expanded to the reaction yields. As shown in Table 1, when substrate **1a** was submitted to the catalytic conditions in the presence of copper catalysts containing four different Tp^*x*^ ligands, a variable amount of product **4a** was obtained. Some relevant information can be obtained from these experiments. There seems to be a correlation between the values of the ν(CO) frequencies in complexes Tp^*x*^Cu(CO) and the catalytic effectivity observed,^14^ although this issue will be further analyzed through DFT calculations.

**Table 1:**
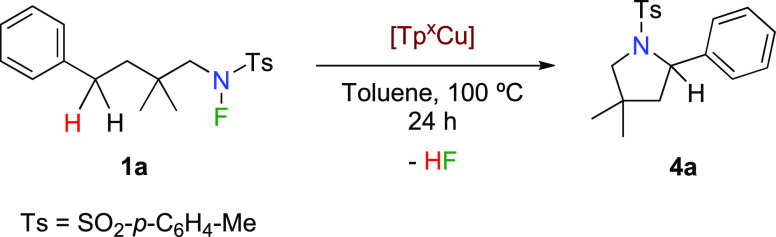
Copper-Catalyzed Intramolecular C–H Amination and Values for ν(CO) (cm^–1^) for [Tp^*x*^Cu(CO)]^a^

entry	Tp^*x*^	yield **4a** [%]^b^	ν(CO) (cm^–1^)
1	Tp^Br3^	60	2110
2	Tp*^,Br^	74	2073
3	Tp*	99	2060
4	Tp^*i*Pr2^	99	2056

a See the Supporting Information for full details. 0.1 mmol **1a** was employed.

b Yields were determined *via*^1^H NMR analysis versus diphenylmethane as the internal standard.

To shed light on the effect of the Tp^*x*^ ligand in this process, we further examined the dependence of the catalyst efficiency on the nature of the substituents at the Tp^*x*^ ligand through a series of DFT calculations. We used the B3LYP-D3 functional with a valence triple-ζ plus polarization and a diffusion basis set for calculations in a continuum toluene solvent. All energies supplied below correspond to free energies, and full computational details are supplied in the Supporting Information.

**Figure 1:**
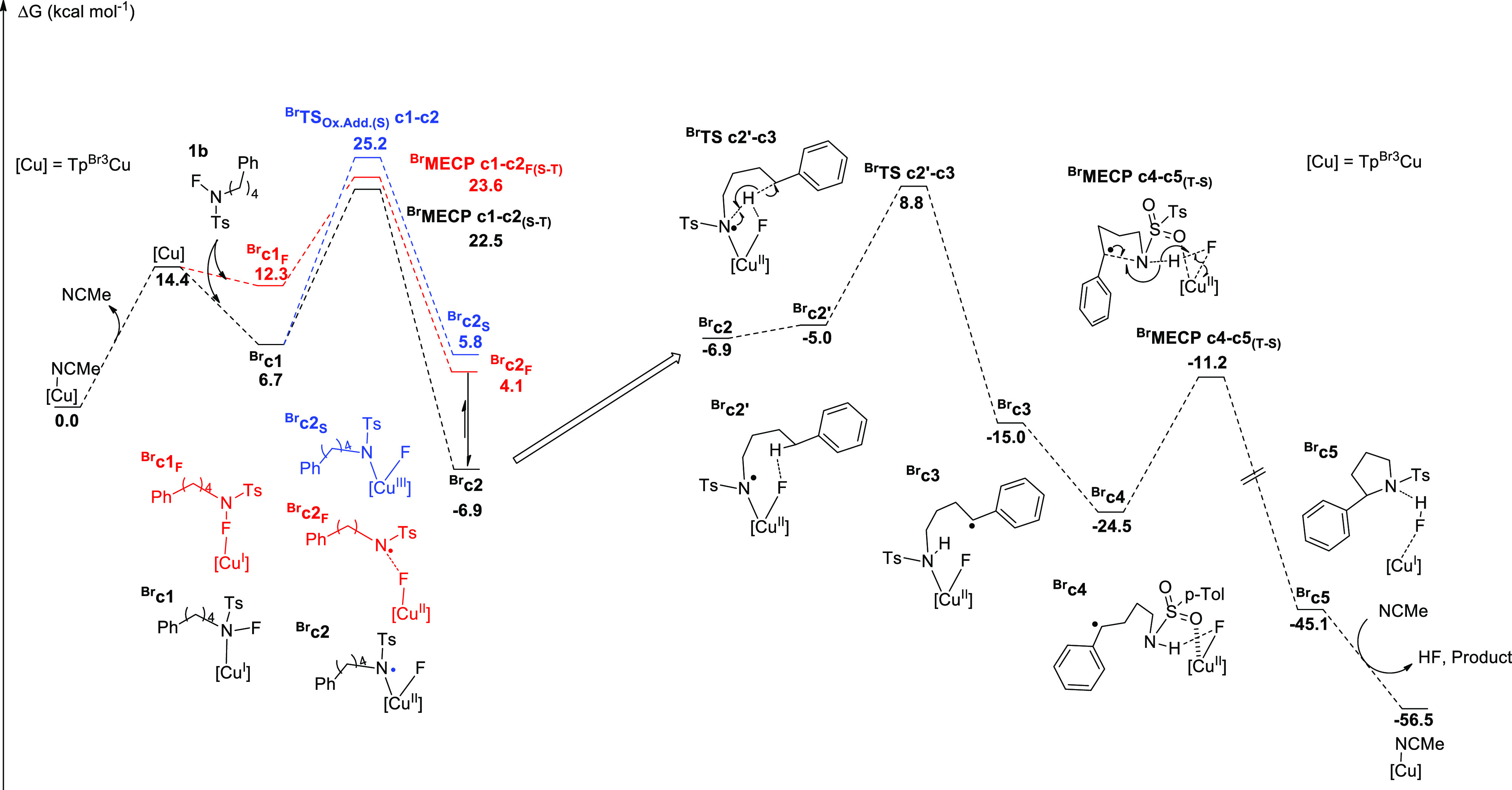
Free energy profile of the reaction with **1b** as the substrate and [Tp^Br3^Cu(NCMe)] as the catalyst. Energies are given in kilocalories per mole.

We chose Tp^Br3^ and Tp* as representative examples for the different behaviors (Table 1) and computed the free energy profiles for each of them. Figure 1 presents the free energy profile for the reaction between the complex containing Tp^Br3^ and **1b** as the substrate (as a simplified model of **1a**). The role of the ligand is apparent in Figure 1, specifically in the early part of the reaction. The corresponding profile for Tp* was already reported in our previous work.^10^ The profiles for the two systems are qualitatively similar and result in intermediates **c2** or ^**Br**^**c2**, where the N–F bond has been broken, and the spin state has changed from singlet to triplet, with the unpaired electrons on nitrogen and on copper, which thus becomes Cu(II). The highest energy point in this path is a minimum energy crossing point (MECP, where the transition from the singlet to the triplet spin state takes place). The need for a spin crossing in this cleavage deserves some comment. The N–F cleavage in a singlet spin state was found to proceed *via*^**Br**^**TS**_**Ox_Add(S)**_**c1-c2**, as shown in Figure 1, and has a higher energy. The N–F cleavage in a triplet spin state is not feasible because the vertical excitation of **c1** from singlet to triplet would be too costly. An additional alternative would be this step proceeding through an open-shell singlet state. The open-shell singlet energy of intermediate ^**Br**^**c2** should certainly be very similar to that of the triplet as there is a little overlap between the two open-shell orbitals. However, the open-shell singlet in ^**Br**^**c1** would be unlikely to converge as it would involve moving an electron from the σ orbital to the σ* orbital of the N–F bond. This highly asymmetric situation would complicate enormously the location of a transition state, and even if it were possible, it would not be very different in the structure/energy from the reported MECP.

The free energy profiles of the N–F cleavage step for Tp^Br3^ and Tp* are qualitatively similar, but they differ significantly on the energy of the key MECP. The resulting barrier for the Tp^Br3^Cu system is 22.5 kcal/mol above reactants through ^**Br**^**MECPc1-c2**_**(S-T)**_ (Figure 1). This is more than 10 kcal/mol above the value for the Tp*Cu system, which was 11.3 kcal/mol. Although a barrier of 22.5 kcal/mol is still affordable in the experimental conditions, it is close to the limit. In addition, it is worth taking into account that the spin transition may be further hindered by a low transition moment.^15^

**Figure 2:**
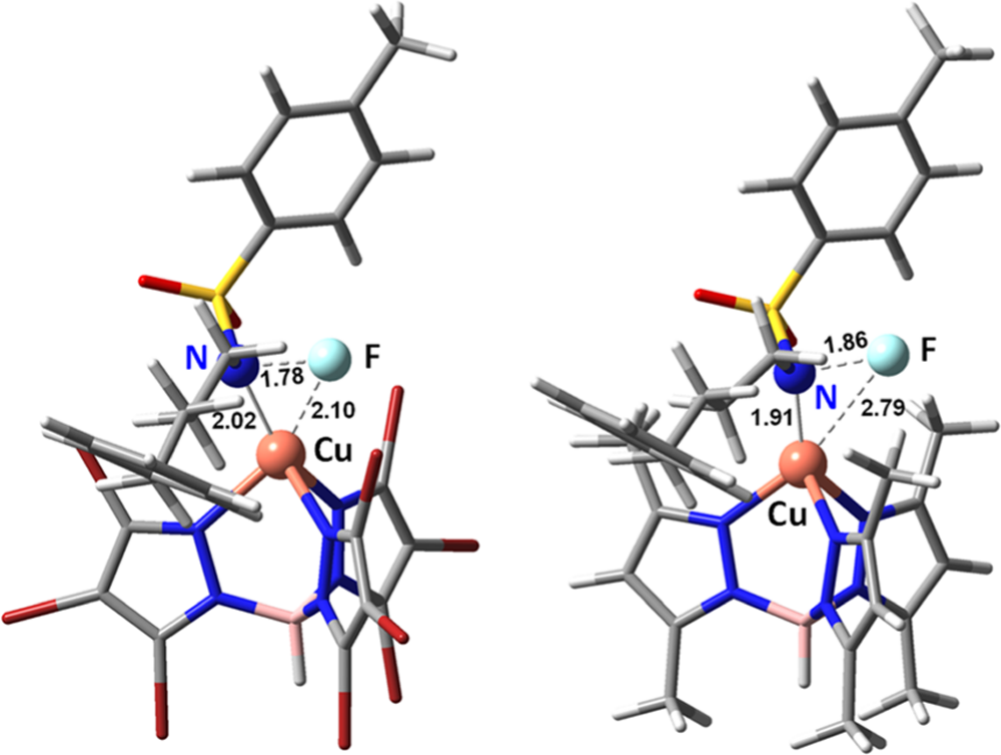
Highest energy point associated to the N–F cleavage for the Tp^Br3^Cu system (left) and the Tp*Cu system (right). Selected distances are given in angstrom.

The origin of the difference between the two systems can be further analyzed by comparing the geometries of the key MECPs, as shown in Figure 2. The origin of the difference seems to be steric rather than electronic. It is clear that in the higher energy Tp^Br3^ system, the fluorine atom is closer to both the nitrogen and copper centers. We consider that in the Tp* system, there is stabilization of the fluorine center due to the presence of favorable dispersion interactions with the methyl substituents in the tris(pyrazolyl)borate ligand, which in this way furnishes a stabilizing pocket for the atom coming into the copper coordination sphere. Such interactions are absent when the methyl groups are replaced by bromine substituents.

### Potential Reaction Pathways

**Scheme 3:**
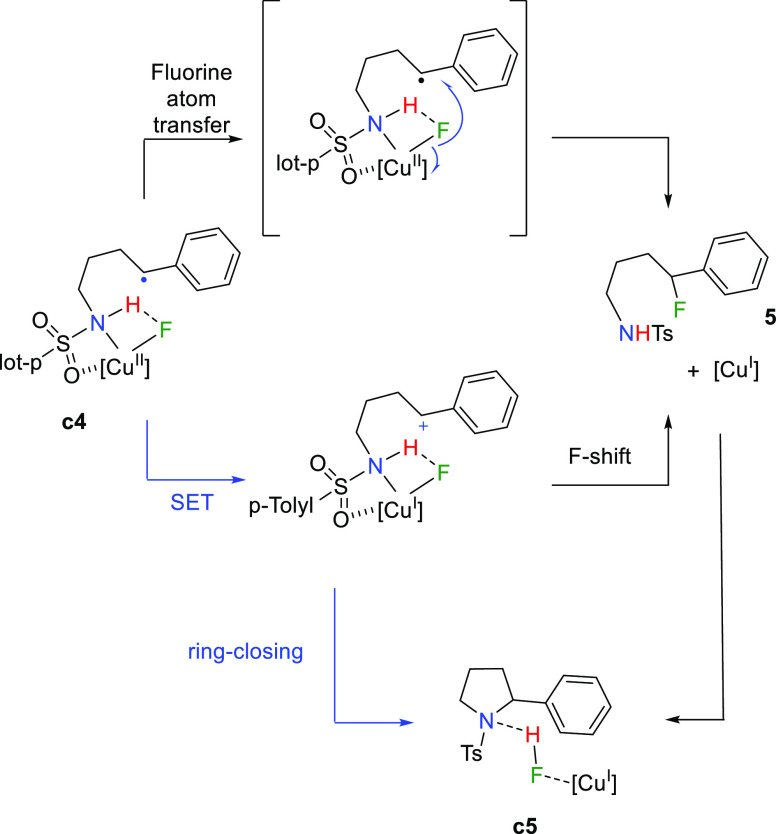
Potential Pathways for C–N Bond Formation from **c4**

In order to explore the final cyclization step in more detail, additional pathways were explored. As shown in Scheme 3, these may include a fluorine atom transfer from copper to the benzylic position, which formally reduces copper back to the initial oxidation state +I. Such a shift could involve a benzylic fluoride intermediate (**5**), which could also be formed from an intramolecular single-electron transfer (SET) between the copper(II) center in **c4** and the benzylic radical through a cationic benzylic intermediate. The formation of the cyclic product could be accessible from such a cation or the [Cu^I^]-**5** couple. The involvement of the benzylic cation proposed in Scheme 3 could not be substantiated via theoretical calculations. In fact, the reductive pathway from **c4** is energetically straightforward.

**Scheme 4:**
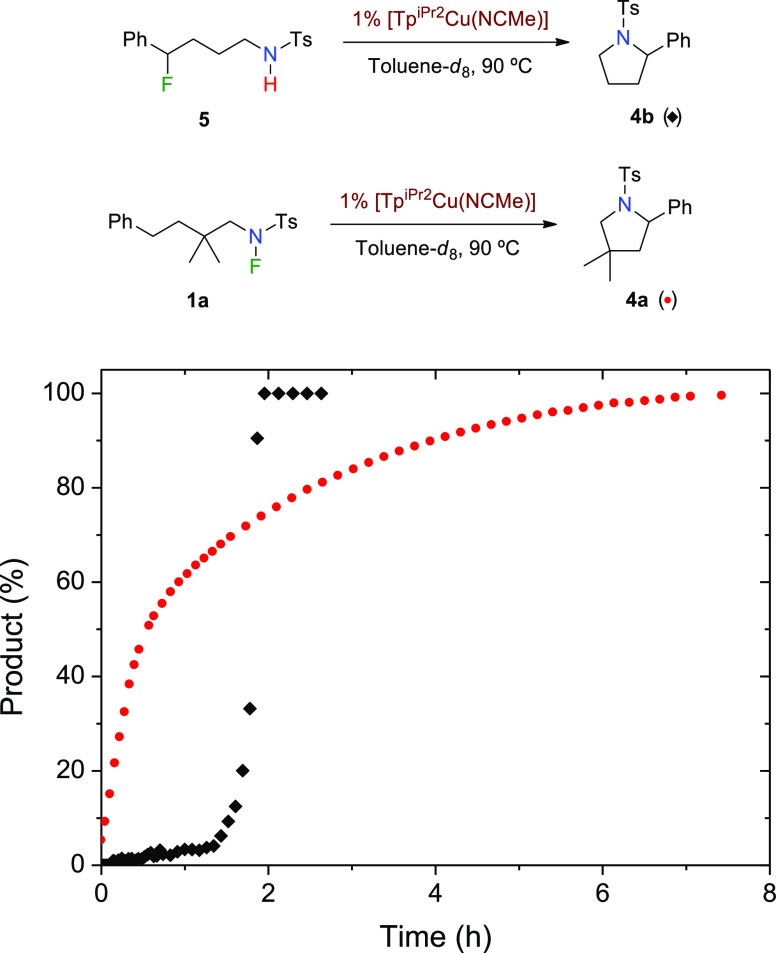
Kinetic Experiments with **1a** and **5** as Reactants

To explore the reactivity of the putative fluorinated intermediate **5**, we have synthesized it individually^16^ and exposed it to the copper catalyst under catalytic conditions (Scheme 4). While no reaction was observed at 25 °C, heating at 90 °C led to the conversion of **5** into **4b**, apparently supporting the former as an intermediate in the reaction mechanism. However, monitoring of appearance of **4b** in two twin experiments employing **1a** and **5** showed a completely distinct profile (Scheme 4). Thus, the conversion of **1a** into **4a** takes place in a smooth manner, whereas the use of **5** as the reactant requires a substantial induction period (Scheme 4). We hypothesize that this period may correspond to a slow process until a sufficient concentration of HF is reached. Importantly, the same performance was observed by employing **5** as a starting material but by adding Brönsted (HF) or Lewis (BF_3_) acids instead of the copper catalyst (see the Supporting Information). At variance with this, **1a** does not react with such Lewis acids *en route* to **4a**. The cleavage of the N–F and C–H bonds involved in the conversion from **1a** to **4a** occurs through a quite specific mechanism dependent on the presence of the copper catalyst, which cannot be replaced in this context by a Lewis acid. This distinct behavior demonstrates that **5** is not an intermediate in the catalytic formation of pyrrolidines from the N–F reactants. Therefore, the conversion of **c4** into **c5** takes place through a SET step, followed by ring closing and F–H formation (Scheme 3, blue path).

### Effect of the Halide Group

**Scheme 5:**
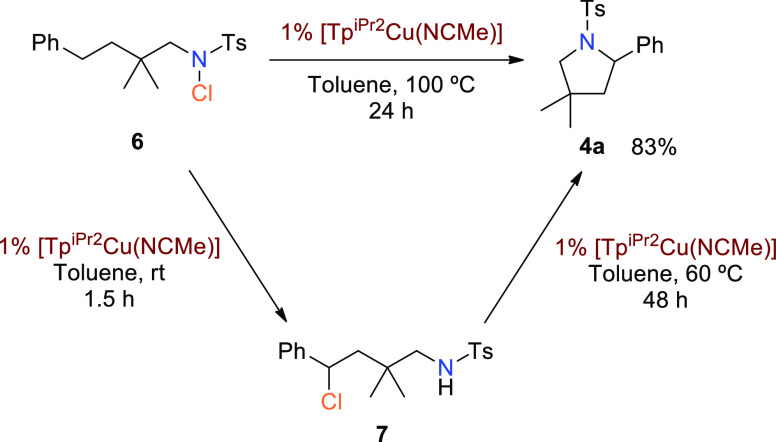
Reactivity of N–Cl and C–Cl Compounds **6** and **7**

We also wondered about the effect that the halide group might exert in the catalytic process. Toward this end, *N*-chlorinated compound **6** was prepared and submitted to catalytic conditions under the same conditions as those employed with the N–F reagent (Table 1, entry 4). The formation of pyrrolidine **4a** (Scheme 5) took place in 83% yield (determined using NMR), a lower value compared with the 99% yield for **4a**. Interestingly, NMR monitoring of the mixture of **6** with the copper catalyst at room temperature for 1.5 h showed the complete conversion into **7**, which further evolves to **4a** and other byproducts upon heating. It cannot be ruled out at this stage that **7** is formed *via* a copper-initiated radical chain reaction^17^ or through thermal Hofmann–Löffler pathways.

In contrast with the above reactivity of **6**, essentially no reaction was observed at 25 °C in the case of a mixture of **1a** with the copper catalyst since heating at 90 °C in toluene-*d*_8_ is needed to afford a clean and complete conversion into **4a** (Scheme 4). Therefore, experimental data assess N–F compounds as the optimum halogenated starting materials for the present C–H amination reaction under copper catalysis.

**Figure 3:**
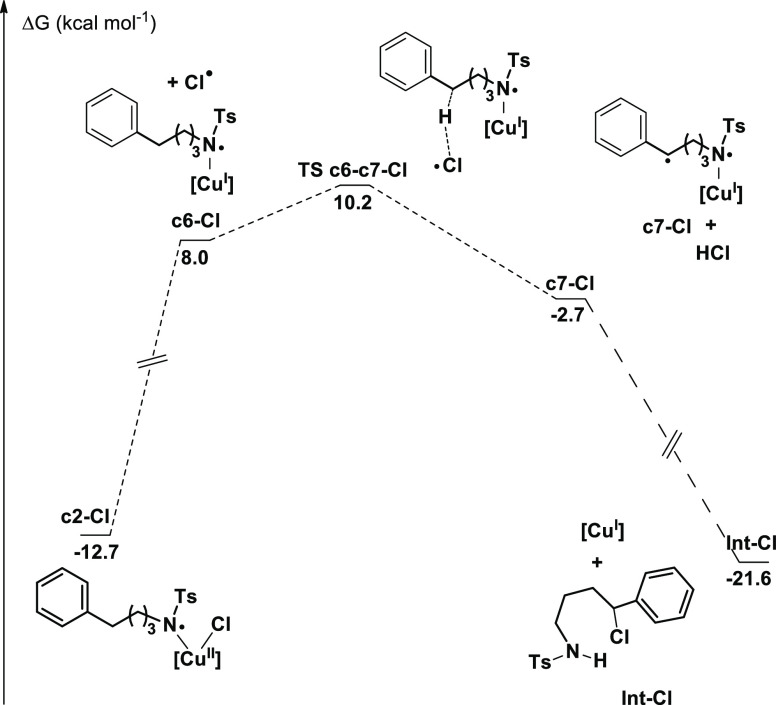
Free energy profile of the C–H activation leading to product **Int-Cl** ([Cu] = Tp*Cu).

The chlorine-based reaction was also examined from a computational point of view with the same method described above. The key difference in this case is associated to the evolution of intermediate **c2**, where the N–X bond has already been broken, and the spins are localized in the copper and nitrogen centers. Results for the N–F system have been presented in Figure 1 and those for N–Cl are shown in Figure 3. The free energy profiles for both processes present significant qualitative differences. We want to remark that the path for the fluorine system is not viable for the chlorine system as it was the starting point for our calculations. In the chlorine system, intermediate **c2-Cl** evolves through the loss of a chlorine radical, which then abstracts a hydrogen from the organic chain, resulting in intermediate **c7-Cl**. The highest point in this path **TS c6-c7-Cl** has a barrier 22.9 kcal/mol above that of **c2-Cl**, much higher than those observed for the N–F systems. There may be alternative paths from **c6-Cl**, but as the reported one in Figure 3 has a TS only 2.2 kcal/mol above this intermediate, we did not consider them. We computed a similar mechanism for the fluorine system, but the barrier was extremely high, with **c6** being 45.4 kcal/mol above **c2**. The much lower stability of the fluorine radical therefore plays a critical role in the compared reactivity of the systems and confirms the unique features of the systems containing the N–F bond.

## Conclusions

Mechanistic studies, both experimental and DFT calculations, on the Tp^*x*^Cu(I)-catalyzed intramolecular C–H amination using *N*-fluoro and *N*-chloro amides have been performed, adding information to previous contributions. The use of fluoride-containing substrates instead of N–Cl ones is largely preferred due to more favorable reaction pathways. Also, the alkyl substituents in the Tp^*x*^ ligand in the [Tp^*x*^CuL] precatalyst induce better conversions, a fact that could be related with an easier Cu(I) to Cu(II) oxidation reaction during the reaction pathway. Evidence for the intermediacy of Cu–F bond formation has been collected. The knowledge obtained from these studies sheds light to the design of a new, more active catalyst for these transformations.

## Supporting Information Available

All experimental procedures and characterization data for new compounds and computational data (PDF)
Cartesian coordinates of the optimized structures (XYZ)

## Supplementary Material

om2c00095_si_001.pdf

om2c00095_si_002.xyz

## Accession Codes

CCDC 2111408 contains the supplementary crystallographic data for this paper. These data can be obtained free of charge via www.ccdc.cam.ac.uk/data_request/cif, or by emailing data_request@ccdc.cam.ac.uk, or by contacting The Cambridge Crystallographic Data Centre, 12 Union Road, Cambridge CB2 1EZ, UK; fax: +44 1223 336033.
