# Supplementary material for: Mechanistic Studies on the Synthesis of Pyrrolidines and Piperidines via Copper-Catalyzed Intramolecular C–H Amination
Source: Organometallics. 2022 Apr 29;41(9):1099–105. doi: 10.1021/acs.organomet.2c00095 (PMC9092462; doi:10.1021/acs.organomet.2c00095)
Supplement: Supplementary file 1 — om2c00095_si_001.pdf [file om2c00095_si_001.pdf]

Supporting Information for

Mechanistic Studies in the Synthesis of Pyrrolidines and Piperidines by Copper-Catalyzed Intramolecular C-H Amination

José María Muñoz-Molina,[a,‡] Daniel Bafaluy,[b,‡] Ignacio Funes-Ardoiz,[b] Adiran de Aguirre,[b] Feliu Maseras,[b,c,*] Tomás R. Belderrain,[a,*] Pedro J. Pérez,[a,*] Kilian Muñiz[a,†]

[a]Laboratorio de Catálisis Homogénea, Unidad Asociada al CSIC, CIQSO-Centro de Investigación en Química Sostenible and Departamento de Química, Universidad de Huelva, 21007 Huelva, Spain

[b]Institute of Chemical Research of Catalonia, ICIQ, The Barcelona Institute of Science and Technology, Av. Països Catalans, 16, 43007 Tarragona, Spain

[c]Departament de Química, Universitat Autònoma de Barcelona, 08193 Bellaterra, Spain

[†]Deceased March 20, 2020.

I	General Information	S-3
II	General protocol (GP 1) for the synthesis of saturated *N*-fluorinated compounds	S-3
III	General protocol (GP 2) for the synthesis of saturated *N*-heterocycles	S-3
IV	General protocol (GP 3) for the synthesis of deuterated compounds	S-4
V	Synthesis of fluorinated compound **5** (GP 4)	S-5
VI	Synthesis of chlorinated compound **6** (GP 5)	S-6
VII	Synthesis of chlorinated compound **7** (GP 6)	S-7
VIII	Deuterium labeling studies	S-7
IX	Effect of the Tp^x ligand	S-8

X	EPR measurements	S-9
XI	Preparation and studies of complex **3**	S-9
XII	Kinetic experiments with **1a** and **5** as reactants. and additional control experiments	S-13
XIII	Studies on compound **6**	S-15
XIV	Analytical data of *N*-fluorinated compounds	S-16
XV	Analytical data of saturated *N*-heterocycles	S-21
XVI	NMR Spectra	S-23
XVII	References experimental part	S-47
XVIII	Computational details	S-48
	References for computational part	S-49

I. General Information

All solvents and reagents employed were purchased from Aldrich, Acros, TCI and Fluorochem. The complexes [Tp^{iPr2}Cu(NCMe)],[1] [Tp^{Br3}Cu(NCMe)],[2] [Tp^{*,Br}Cu(NCMe)],[3] [Tp*Cu(NCMe)],[4] and [TpMsCu(THF)][5] were prepared according to literature. Column chromatography was performed on silica gel (PanReac AppliChem, Silica Gel 60, 0.063-0.2 mm). NMR spectroscopy was performed on a Bruker Avance 300 MHz, 400 MHz or 500 MHz, respectively. The chemical shifts are given in ppm normalized to the shift of residual chloroform in the deuterated chloroform (δ_H = 7.26 ppm and δ_C = 77.16 ppm). The multiplicities are stated as follows: s = singlet, bs = broad singlet, d = doublet, t = triplet, q = quartet, m = multiplet. HRMS measurements were performed on a Kratos MS 50. Continuous wave (CW) EPR spectra were obtained at room temperature on a Bruker EMX Micro X-band spectrometer operating at 9.826e9 using a Bruker ER 1164 HS resonator. IR spectroscopy was performed using a Bruker Alpha instrument in the solid state. Melting points were determined employing a Büchi B-540 instrument.

II. General protocol (GP 1) for the synthesis of *N*-fluorinated compounds

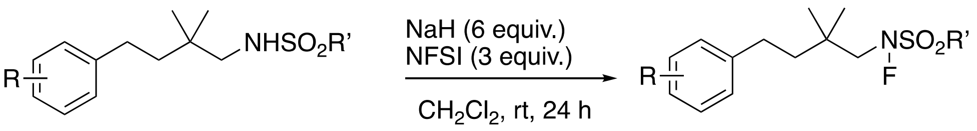

A solution of the corresponding sulfonamide[6,7,9] (0.8 mmol) in dry CH_2Cl_2 (7 mL) was added to a stirred mixture of NaH (4.8 mmol) in dry CH_2Cl_2 (7 mL) at 25 °C. After 30 min, NFSI (2.4 mmol) in CH_2Cl_2 (7 mL) was added dropwise at 25 °C. The reaction was stirred for 24 h and monitored by TLC. After 24 h, the reaction was quenched with NH_3 (2%)/NaOH (6.5%) solution (20 mL) at 0 °C. The reaction mixture was extracted with Et_2O (3 x 15 mL) and the combined organic layers were washed with NH_3/NaOH solution (3 x 15 mL), NaOH (5%) (3 x 15 mL) and HCl (5%) (3 x 15 mL), dried over $MgSO_4$, filtered and the solvent was removed under reduced pressure. The residue was purified by column chromatography on silica gel using hexane/ethyl acetate.

III. General protocol (GP 2) for the synthesis of saturated *N*-heterocycles

A flame-dried schlenk tube was charged with the corresponding *N*-fluorotosylamide (0.1 mmol), [Tp^{iPr2}Cu(NCMe)] (1 mol%) and dry toluene under an atmosphere of argon. The reaction mixture was stirred at 100 °C. After 24 h, the reaction mixture was cooled down to room temperature and the solvent was removed under reduced pressure. The thus obtained crude product was further purified by column chromatography on silica gel using hexane/ethyl acetate.

IV. General protocol (GP 3) for the synthesis of deuterated compounds

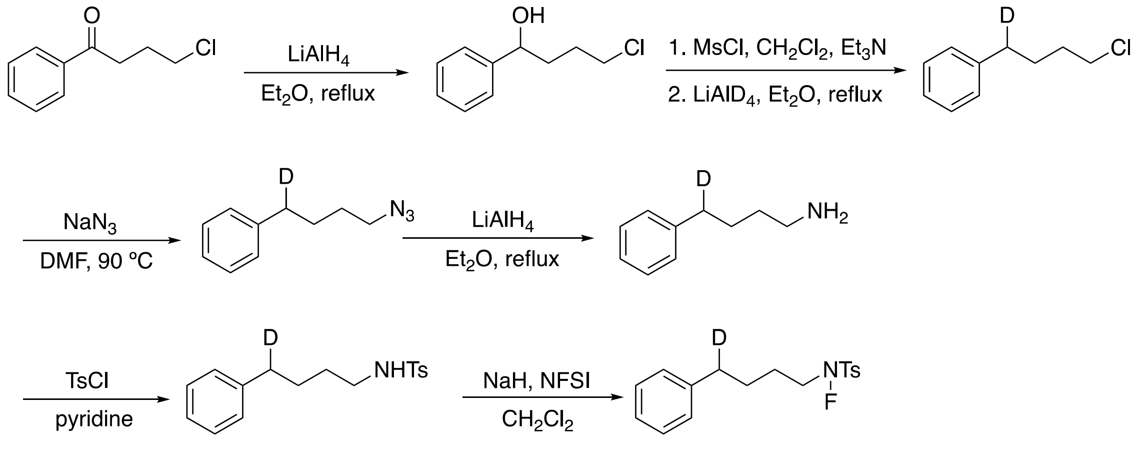

Deuterated compounds were synthesized by the above outlined synthesis using 4-chlorobutyrophenone as commercially available starting material.

Note: For the *gem*-di-deuterated compound, the first step was performed with $LiAlD_4$ instead of $LiAlH_4$.

Note: For the corresponding compounds for 6-membered ring formation step 4 was performed with KCN instead of NaN_3.

Step 1:

4-Chlorobutyrophenone (0.55 g, 3.0 mmol, 1 eq.) was dissolved in 10 mL of dry diethyl ether and carefully added to a suspension of $LiAlH_4$ (0.11 g, 3.0 mmol, 1 eq.) in 20 mL diethyl ether at 0 °C. The mixture was refluxed for 2 h after which the reaction was cooled to 0 °C and quenched by careful addition of a 2 M aqueous NaOH solution. The mixture was filtered over Na_2SO_4 and the solvent was removed under reduced pressure. The crude product was further purified by column chromatography (9:1, hexane/ethyl acetate).

Step 2:

4-Chloro-1-phenylbutan-1-ol (0.35 g, 1.9 mmol, 1 eq.) was dissolved in dry dichloromethane and cooled to 0 °C. Triethylamine (0.39 g, 3.8 mmol, 2 eq.) was added and the mixture was stirred for 10 min at 0 °C. Mesyl chloride (0.24 g, 2.1, 1.1 eq.) was added dropwise at 0 °C and the reaction mixture was stirred for 30 min at 0 °C after which the reaction was quenched by addition of a sat. aqueous $NaHCO_3$. The layers were separated and the aqueous phase was extracted with dichloromethane. The combined organic phases were dried over Na_2SO_4, filtered and the solvent was removed under reduced pressure to yield the desired mesylate which was used without purification for the next step.

Note: Due to rapid decomposition of the mesylate, the next step has to be done immediately after mesylation.

Step 3:

The crude mesylate from step 2 was dissolved in dry diethyl ether and the solution was cooled to 0 °C. Subsequently, $LiAlD_4$ (0.08 g, 1.9 mmol, 1 eq.) was carefully added at 0 °C after which the reaction mixture was refluxed for 2 h. The reaction mixture was cooled to 0 °C and quenched by careful addition of a 2 M aqueous NaOH solution. The mixture was filtered over Na_2SO_4 and the solvent was removed under reduced pressure. The crude product was further purified by column chromatography (hexane).

Step 4:

(4-Chlorobutyl-1-*d*)benzene (0.07 g, 0.41 mmol, 1 eq.) was dissolved in dry DMF and NaN_3 (0.04 g, 0.62 mmol, 1.5 eq.) was added. The reaction mixture was stirred overnight at 90 °C. Then water was added and the aqueous layer was extracted with ethyl acetate. The combined organic layers were dried over Na_2SO_4, filtered and the solvent was removed under reduced pressure. The crude product was directly used for the next step.

Step 5:

The crude product from step 4 was dissolved in dry diethyl ether, the solution was cooled to 0 °C and $LiAlH_4$ (0.047 g, 1.2 mmol, 3 eq.) was added carefully at that temperature. The reaction mixture was refluxed for 2h after which the mixture was cooled again to 0 °C and quenched by careful addition of a 2 M aqueous NaOH solution. The mixture was filtered over Na_2SO_4 and the solvent was removed under reduced pressure. The crude product was directly used for the next step.

Step 6:

The crude product from step 5 was dissolved in pyridine and cooled to 0 °C. Tosyl chloride (0.094 g, 0.49 mmol, 1.2 eq.) was added at once, the reaction mixture was allowed to warm to room temperature and stirred overnight at room temperature. The reaction mixture was diluted with dichloromethane and washed with 1 M HCl. The organic phase was dried over Na_2SO_4, filtered and the solvent was removed under reduced pressure. The crude product was further purified by column chromatography (9:1, hexane/ethyl acetate).

Step 7:

For the last step **GP2** was followed.

V. Synthesis of fluorinated compound 5 (GP 4)

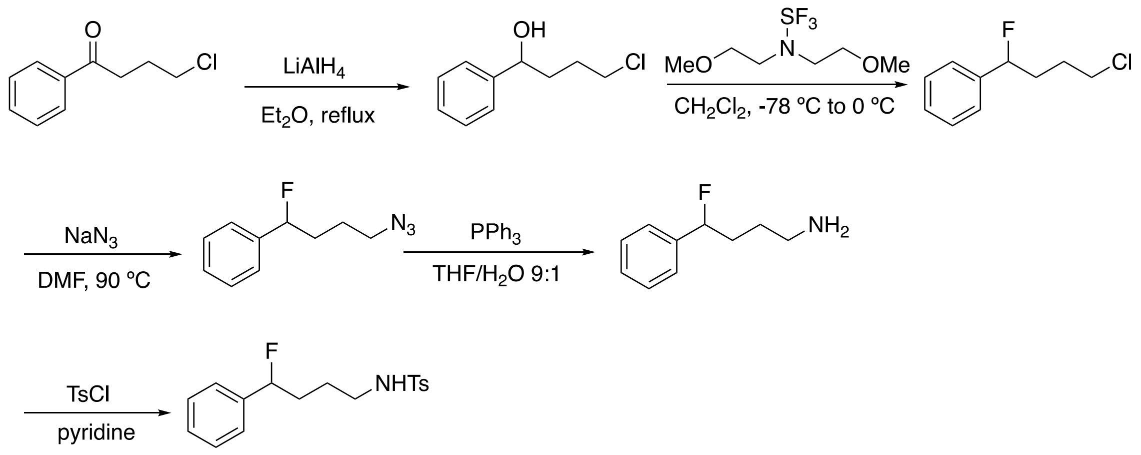

Step 1:

4-Chlorobutyrophenone (0.800 g, 4.3 mmol, 1 eq.) was dissolved in 10 mL of dry diethyl ether and carefully added to a suspension of $LiAlH_4$ (0.332 g, 8.6 mmol, 2 eq.) in 15 mL diethyl ether at 0 °C. The mixture was refluxed for 2 h after which the reaction was cooled to 0 °C and quenched by careful addition of a 2 M aqueous NaOH solution. The mixture was filtered over Na_2SO_4 and the solvent was removed under reduced pressure. The crude product was further purified by column chromatography (9:1, hexane/ethyl acetate).

Step 2:

4-chloro-1-phenylbutan-1-ol (0.457 g, 2.47 mmol, 1 eq.) was dissolved in 10 mL of dry dichloromethane and cooled down to -78 °C. Commercially available solution of Deoxofluor in toluene (1.31 mL, 2.97 mmol, 1.2 eq.) was added dropwise and the mixture was stirred at the same temperature for 30 minutes. The reaction was allowed to warm up to 0 °C and was stirred for extra 2 h. The reaction was quenched with saturated aqueous $NaHCO_3$ solution and extracted with dichloromethane. The combined organic phases were dried over Na_2SO_4 and the solvent was removed under reduced pressure. The crude product was further purified by column chromatography (hexane).

Step 3:

(4-chloro-1-fluorobutyl)benzene (0.326 g, 1.78 mmol, 1 eq.) was dissolved in dry DMF and NaN_3 (0.170 g, 2.62 mmol, 1.5 eq.) was added. The reaction mixture was stirred overnight at 90 °C. Then water was added and the aqueous layer was extracted with ethyl acetate. The combined organic layers were dried over Na_2SO_4, filtered and the solvent was removed under reduced pressure. The crude product was directly used for the next step.

Step 4:

(4-azido-1-fluorobutyl)benzene (0.152, 0.79 mmol, 1 eq.) was dissolved in 1 mL of a 10:1 mixture of THF and H_2O. Triphenylphosphine (0.310 g, 1.2 mmol, 1.5 eq.) was added and the reaction was stirred overnight at room temperature. The reaction mixture was diluted with ethyl acetate and extracted with water. The organic phase was dried over Na_2SO_4, filtered and the solvent was removed under reduced pressure. The crude product was further purified by column chromatography (9:1, CH_2Cl_2/MeOH).

Step 5:

4-fluoro-4-phenylbutan-1-amine (0.090 g, 0.54 mmol, 1 eq.) was dissolved in pyridine and cooled to 0 °C. Tosyl chloride (0.124 g, 0.65 mmol, 1.2 eq.) was added at once, the reaction mixture was allowed to warm to room temperature and stirred overnight at room temperature. The reaction mixture was diluted with dichloromethane and washed with 1 M HCl. The organic phase was dried over Na_2SO_4, filtered and the solvent was removed under reduced pressure. The crude product was further purified by column chromatography (9:1, hexane/ethyl acetate).

VI. Synthesis of chlorinated compound 6 (GP 5)

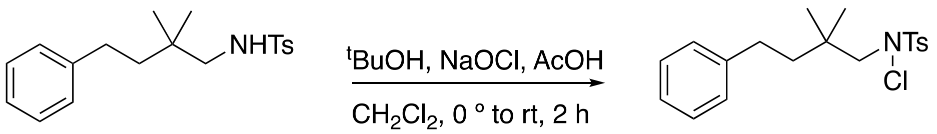

Note: To avoid decomposition of the *in situ* formed *tert*-butyl hypochlorite and/or of the product, the fume hood lights were switched off.

N-(2,2-dimethyl-4-phenylbutyl)-4-methylbenzenesulfonamide (0.520 g, 1.57 mmol, 1 eq.) was dissolved in 10 mL of dichloromethane and cooled down to 0 °C. Glacial acetic acid (0.359 mL, 6.28 mmol, 4 eq.), *tert*-butanol (0.601 mL, 6.28 mmol, 4 eq.) and commercially available NaOCl 14% aqueous solution (2.96 mL, 6.28 mmol, 4 eq.) were subsequentially added. After stirring at 0 °C for 30 minutes, the reaction was allowed to warm up to room temperature and was stirred for an extra 1.5 hours. The reaction was diluted with dichloromethane and extracted with

water. The organic phase was dried over Na_2SO_4, filtered and the solvent was removed under reduced pressure. The crude product was further purified by column chromatography (95:5, hexane/ethyl acetate).

VII. Synthesis of chlorinated compound 7 (GP 6)

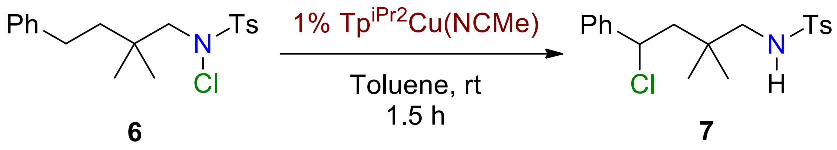

Under an atmosphere of N_2, a glass ampoule was charged in turn with **6** (0.1 mmol), Tp^{iPr2}Cu(NCMe) (0.001 mmol, 1%), and toluene (3.0 mL). The ampoule was sealed with a Teflon screw cap, and then the reaction mixture was stirred at 25 ºC. After 1.5 h, the volatiles were removed under reduced pressure. The crude product was further purified by column chromatography (9:1, hexane/ethyl acetate).

VIII. Deuterium labeling studies

The experiment for the intramolecular KIE was carried out following the general protocol 2 (**GP2**) using toluene-d^8 as solvent. The progress of the reaction was followed by ^{1}H-NMR at 100 °C. Integration of the ^{1}H NMR spectrum of the product mixture revealed an isotope effect k_H/k_D = 3.3.

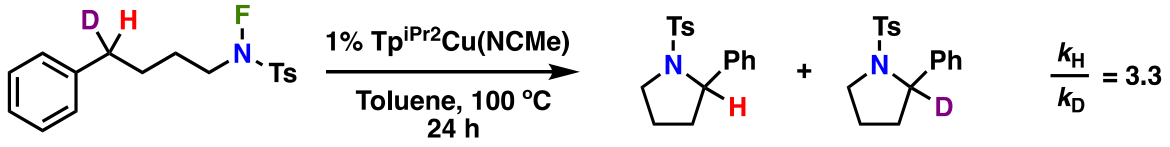

The experiment for the intermolecular competition KIE was carried out following the general protocol 1 (**GP1**) stopping the reaction after 4 h. Integration of the ^{1}H NMR spectrum of the product mixture revealed an isotope effect k_H/k_D = 1.4.

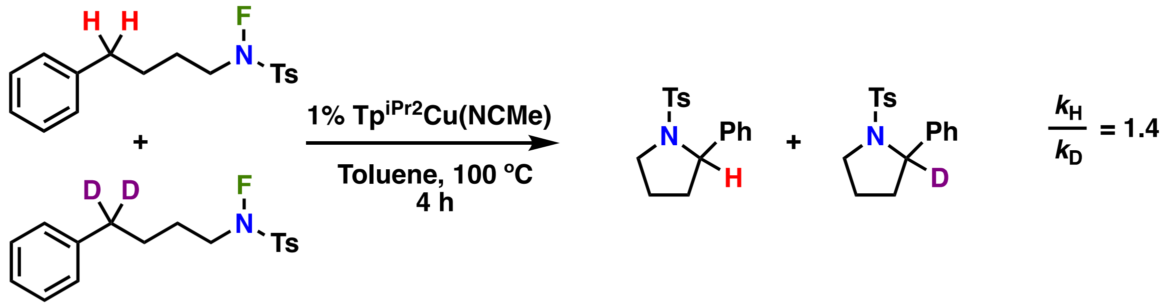

The experiment for the intramolecular KIE in the piperidine formation series was carried out following the general protocol 2 (**GP2**). Integration of the ^{1}H NMR spectrum of the product mixture revealed an isotope effect k_H/k_D = 4.2.

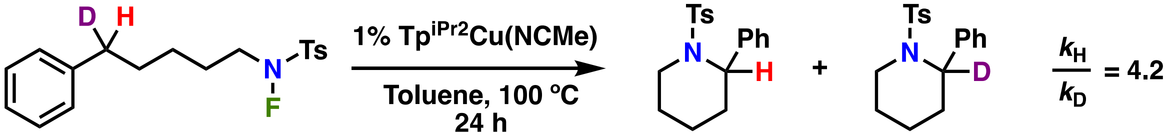

The experiment for the intermolecular KIE in the piperidine formation series was carried out following the general protocol 2 (**GP2**) stopping the reaction after 4 h. Integration of the ^{1}H NMR spectrum of the product mixture revealed an isotope effect k_H/k_D = 1.35.

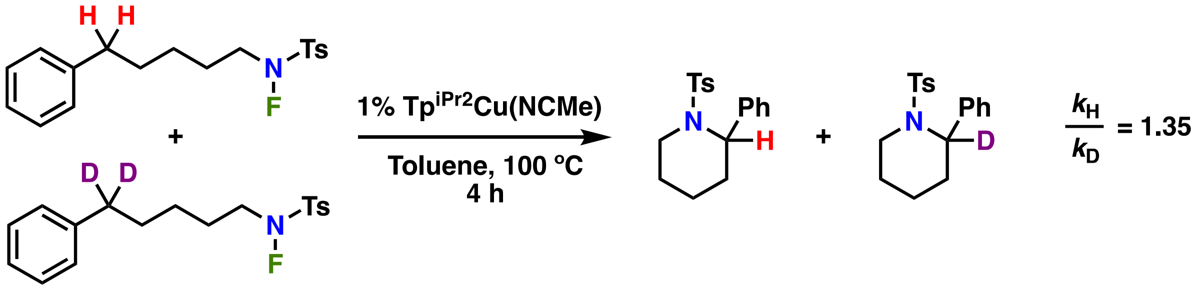

IX. Effect of the Tp^{x} Ligand

Under an atmosphere of N_2, a glass ampoule was charged successively with *N*-fluorinated amine (0.1 mmol), [Tp^{x}Cu(NCMe)] (0.001 mmol, 1%), and toluene (1.5 mL). The ampoule was sealed with a Teflon screw cap, and then the reaction mixture was stirred at 100 °C. After 24 h, the reaction was cooled down to room temperature and the volatiles were removed under reduced pressure. Diphenylmethane (17 µL) was added as standard, and the reaction was analyzed by ^{1}H NMR ($CDCl_3$). The corresponding individual results are depicted in the main manuscript.

X. EPR measurements

The spectral data (see below) was collected at 298 K with the following spectrometer settings: g-factor = 2.000000, microwave power = 2.05 mW; center field = 3200.00 G, sweep width = 6000 G, sweep time = 60 s, modulation frequency = 100 KHz, modulation amplitude = 4 G, power attenuation = 20 dB, time constant = 0.01 ms, conversion time = 4 ms, gain = 20 dB.

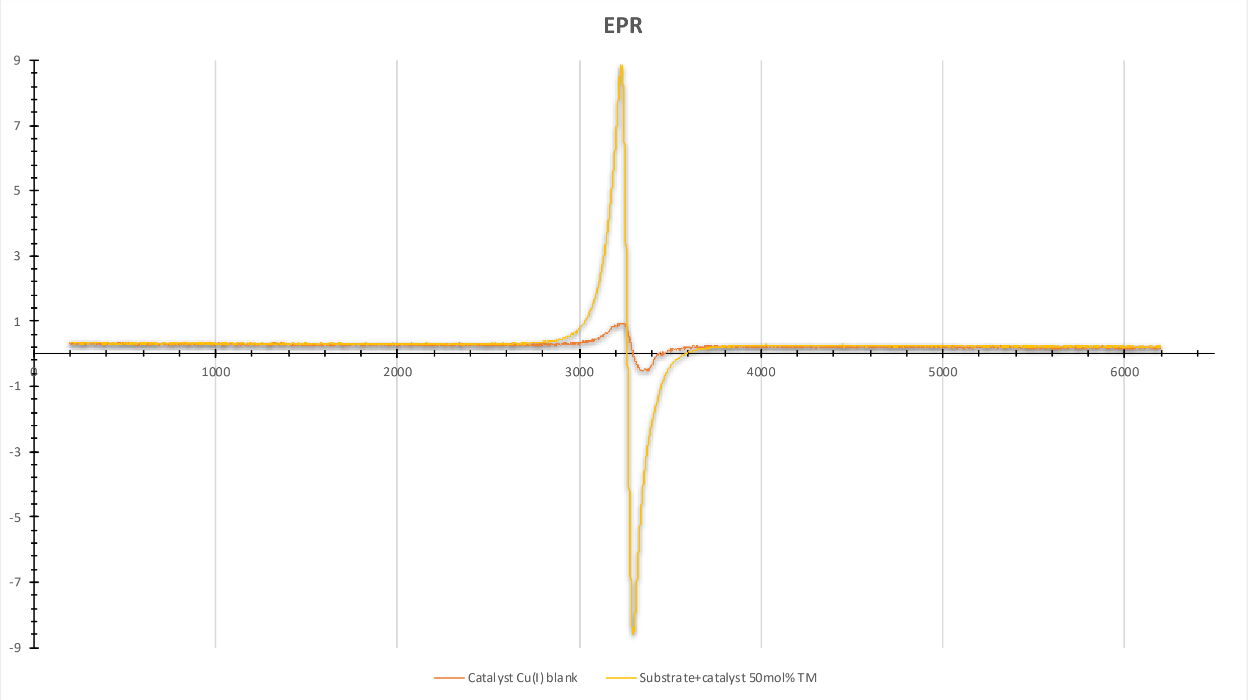

Figure S1. EPR spectra.

A blanck solution of [Tp^{iPr2}Cu(NCMe)] 0.5M in dry toluene was first measured as blank solution (orange line). A small Cu(II) signal was observed due to aerobic oxidation from the molecular oxygen present in the solvent. A second equimolar solution [Tp^{iPr2}Cu(NCMe)] and **1k** 0.5M in dry toluene was measured (yellow line). A sharp Cu(II) signal appears confirming that the N-fluorinated substrate is capable to oxidize the catalyst.

XI. Preparation and studies of complex 3

Complex **3** was first obtained from an equimolar mixture of substrate **1b** with [Tp^{iPr2}Cu(NCMe)] in toluene under air after crystallization at 0 °C. A separate synthesis was designed as follows. In a capped vial, [Tp^{iPr2}Cu(NCMe)] (15mg) was dissolved in toluene (1 mL). The resulting solution

was stored partially uncapped at 4-5ºC for 5 days. After such time, green crystals of complex **3** were collected, separated upon toluene decantation and weight under air. Yield = 10%.

X-Ray Analytical Data for Compound 3

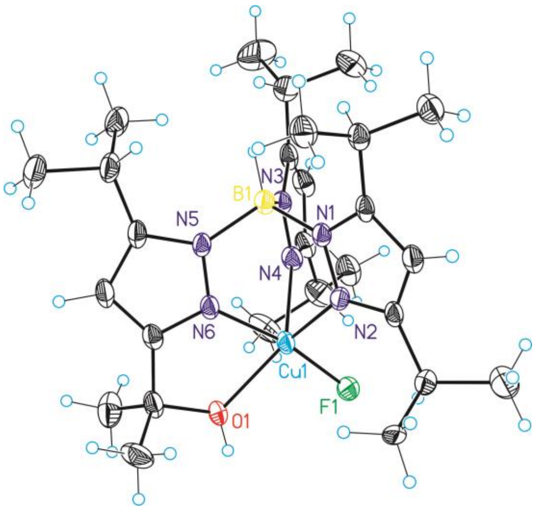

Figure S2. ORTEP view of compound 3

Table S1. Crystal data and structure refinement for 3.

Identification code	**3**	
Empirical formula	C34 H54 B Cu F N6 O	
Formula weight	656.18	
Temperature	100(2) K	
Wavelength	0.71073 Å	
Crystal system	Monoclinic	
Space group	P2(1)/n	
Unit cell dimensions	a = 11.5174(7)Å	a= 90°.
	b = 13.6246(8)Å	b = 92.828(2)°.
	c = 22.9510(15)Å	g = 90°.
Volume	3597.1(4) Å^3	
Z	4	
Density (calculated)	1.212 Mg/m^3	
Absorption coefficient	0.646 mm^{-1}	
F(000)	1404	
Crystal size	0.02 x 0.02 x 0.02 mm^3	

Theta range for data collection	1.739 to 27.570°.
Index ranges	-14<=h<=14,-16<=k<=17,-29<=l<=29
Reflections collected	34712
Independent reflections	8292[R(int) = 0.0567]
Completeness to theta =27.570°	99.7%
Absorption correction	Multi-scan
Max. and min. transmission	0.987 and 0.93
Refinement method	Full-matrix least-squares on F^2
Data / restraints / parameters	8292/ 142/ 484
Goodness-of-fit on F^2	1.030
Final R indices [I>2sigma(I)]	R1 = 0.0497, wR2 = 0.1042
R indices (all data)	R1 = 0.0898, wR2 = 0.1180
Largest diff. peak and hole	0.706 and -0.394 e.Å^{-3}

Mass spectrometry measurement of the mixture of [Tp^{iPr2}Cu(NCMe)] with NFSI under air showed the presence of complex **3** [Tp^{iPr2}OHCuF(NCMe)] (m/z=604.3) and its precursor [Tp^{iPr2}CuF(NCMe)] (m/z=588.34).

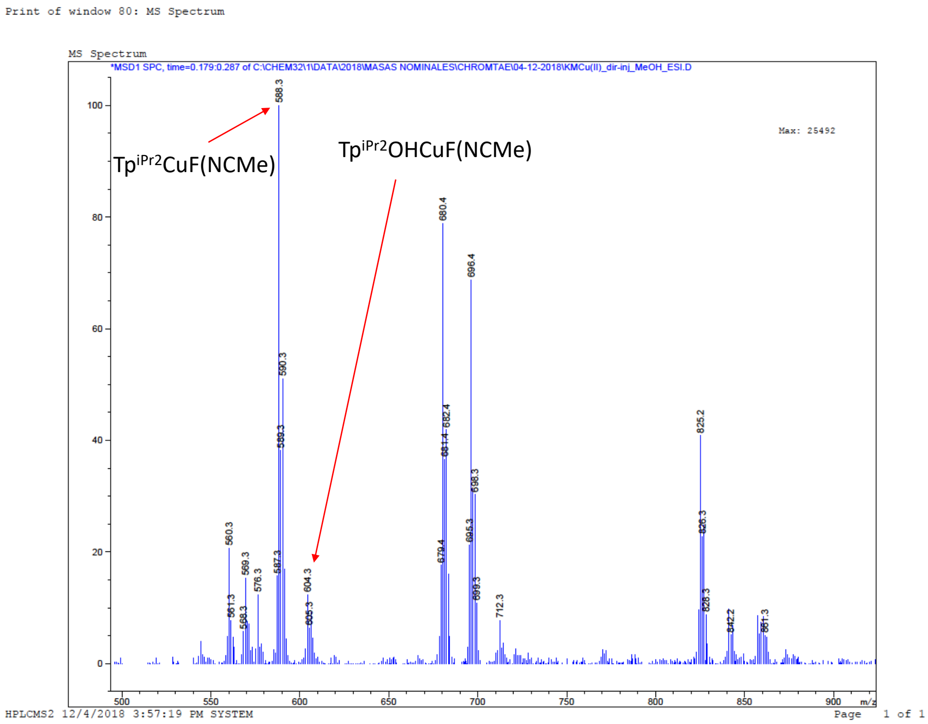

Figure S3. ESI-MS of the above experiment.

Kinetic studies with **3**:

[Tp^{iPr2}Cu(NCMe)] (16 mg, 0.028 mmol) and NFSI (8.8 mg, 0.028 mmol) were dissolved in 1 mL of toluene-*d*^{8}. The resulting green solution presented a fine solid in suspension which was filtered off. 21 μL of the filtrate were added to a previously prepared solution of **1b** (18mg, 0.0589 mmol) in 0.88 mL of toluene-*d*^{8}. The progress of the reaction was followed by ^{1}H-NMR at 100 °C.

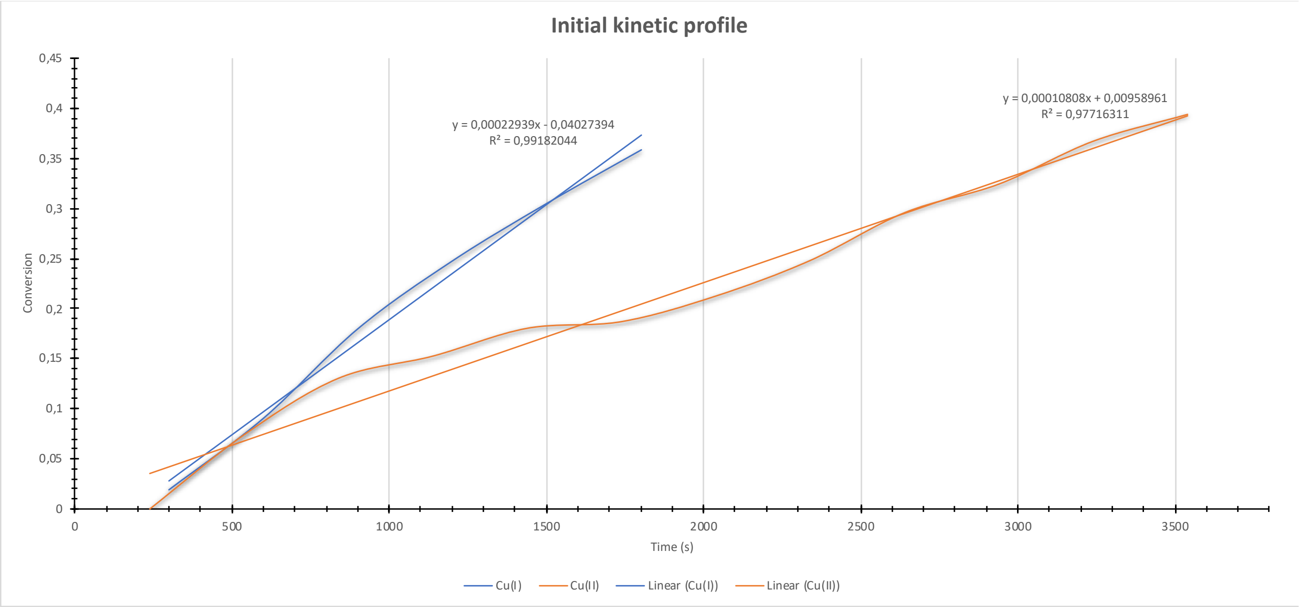

Figure S4. Kinetic studies with complex **3**

Compound **3** is capable to promote the cyclization (orange line). However, its activity (kinetic competence) is approximately half that the one of the standard catalyst [Tp^{iPr2}Cu(NCMe)] (blue line). This observation is consistent with compound **3** being an off-cycle species.

XII. Kinetic experiments with 1a and 5 as reactants and additional control experiments

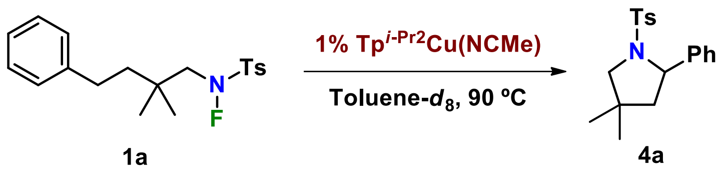

In a glovebox, 10.5 mg (0.03 mmol) of **1a**, 35 μL of a stock solution of the complex Tp^{iPr2}Cu(NCMe) (0.0003 mmol) in toluene-d_8, and 465 μL of toluene-d_8 were added to a NMR pressure tube and sealed with a Teflon screw cap. The reaction was then monitored by ^{1}H NMR spectroscopy. The graphical representation of **4a** (%) *vs* time (h) is shown below.

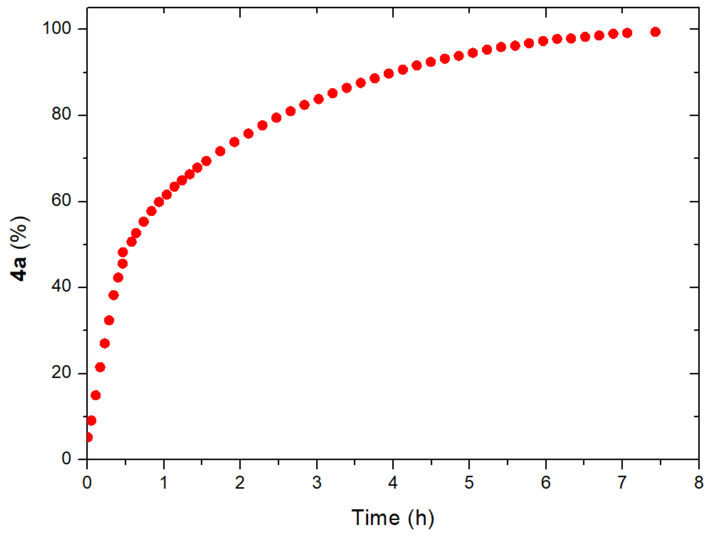

Figure S5. Kinetic monitoring of this experiment

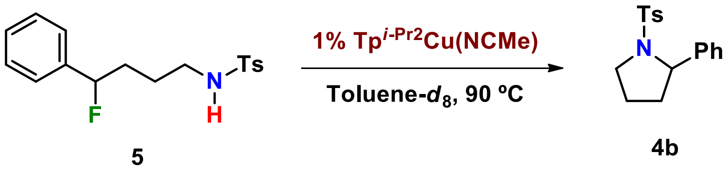

Under an atmosphere of N_2, 9.6 mg (0.03 mmol) of **5**, 35 μL of a stock solution of the complex Tp^{iPr2}Cu(NCMe) (0.0003 mmol) in toluene-d_8, and 465 μL of toluene-d_8 were added to a NMR pressure tube and sealed with a Teflon screw cap. The reaction was then monitored by ^{1}H NMR spectroscopy. The graphical representation of **4b** (%) *vs* time (h) is shown below.

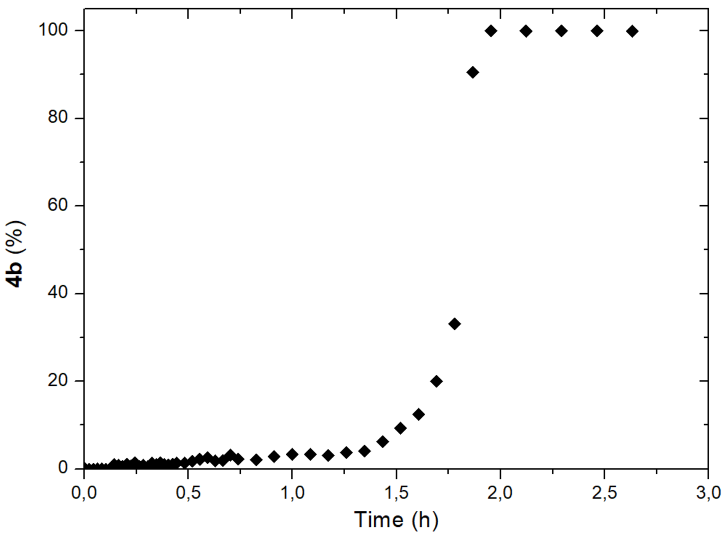

Figure S6. Kinetic monitoring of this experiment

Additional control experiments:

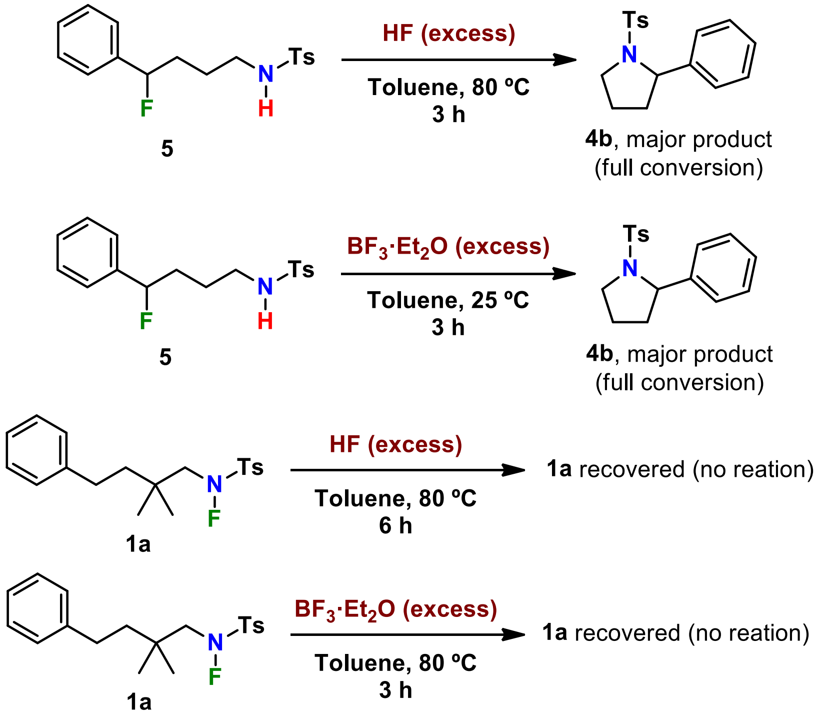

XIII. Studies on compound 6

Reaction monitoring of **6**:

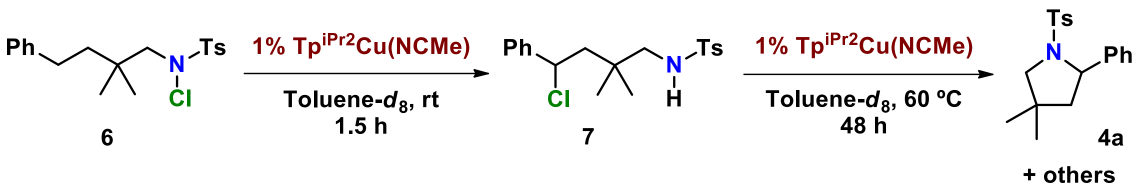

In a glovebox, 6.2 mg (0.017 mmol) of **6**, 20 µL of a stock solution of the complex $Tp^{iPr2}Cu(NCMe)$ (0.00017 mmol) in toluene-d_8, and 480 µL of toluene-d_8 were added to a NMR pressure tube and sealed with a Teflon screw cap. The reaction was then monitored by ^1H NMR spectroscopy.

XIV. Analytical data of *N*-fluorinated compounds

_N_-(2,2-Dimethyl-4-phenylbutyl)-_N_-fluoro-4-methylbenzenesulfonamide (1a)

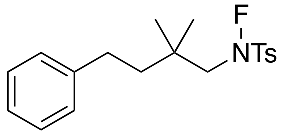

1a was obtained as a white solid (65%) by applying **GP 1**.
^{1}H NMR (400 MHz, CDCl$_3$): δ = 7.88-7.84 (m, 2H), 7.46-7.42 (m, 2H), 7.33-7.28 (m, 2H), 7.20-7.15 (m, 3H), 3.12 (d, *J* = 44.4 Hz, 2H), 2.63-2.55 (m, 2H), 2.52 (s, 3H), 1.73-1.63 (m, 2H), 1.08 (s, 6H).
^{13}C{^{1}H} NMR (100 MHz, CDCl$_3$): δ = 146.3, 142.7, 130.1, 129.9, 129.6, 128.5, 128.5, 125.9, 62.9 (d, *J* = 10.6 Hz), 42.4, 34.6, 30.4, 25.8, 21.9.
^{19}F NMR (375 MHz, CDCl$_3$): δ = -36.38.
IR ν (cm^{-1}): 3028, 2958, 1954.
mp: 72-73 °C.
HRMS: Mass calculated for $C_{19}H_{24}FNNaO_2S$: 372.1404, found: 372.1402.

_N_-Fluoro-4-methyl-_N_-(4-phenylbutyl)benzenesulfonamide (1b)

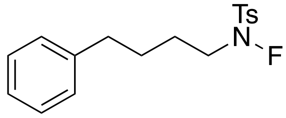

1b was obtained as a white solid (59%) by applying **GP 1**.
^{1}H NMR (400 MHz, CDCl$_3$): δ = 7.81 (d, *J* = 8.3 Hz, 2H), 7.40 (d, *J* = 8.1 Hz, 2H), 7.30-7.25 (m, 2H), 7.21-7.14 (m, 3H), 3.30-3.14 (m, 2H), 2.67-2.61 (m, 2H), 2.48 (s, 3H), 1.79-1.71 (m, 4H).
^{13}C{^{1}H} NMR (100 MHz, CDCl$_3$): δ = 146.3, 141.9, 130.1, 130.1, 129.1, 128.5, 126.0, 53.6 (d, 12.4 Hz), 35.4, 28.4, 26.0, 21.9.
^{19}F NMR (375 MHz, CDCl$_3$): δ = -49.8.
IR ν (cm^{-1}): 3027, 2917, 2862, 1593.
mp: 67-69 °C.
HRMS: Mass calculated for $C_{17}H_{20}FNNaO_2S$: 344.1091, found: 344.1088.

_N_-fluoro-4-methyl-_N_-(2-methyl-4-phenylbutyl)benzenesulfonamide (1c)

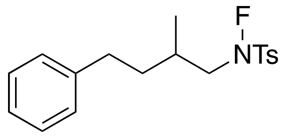

1c was obtained as a yellow oil (62%) by applying **GP 1**.

^{1}H NMR (400 MHz, CDCl$_3$): δ = 7.81 (d, *J* = 8.4 Hz, 2H), 7.40 (d, *J* = 8.2 Hz, 2H), 7.30-7.24 (m, 2H), 7.21-7.13 (m, 3H), 3.10 (dt, *J* = 42.1 Hz, 6.4, 2H), 2.73-2.64 (m, 1H), 2.61-2.53 (m, 1H), 2.49 (s, 3H), 1.99-1.89 (m, 1H), 1.86-1.75 (m, 1H), 1.58-1.46 (m, 1H), 1.07 (d, *J* = 6.7 Hz, 3H).
^{13}C{^{1}H} NMR (100 MHz, CDCl$_3$): δ = 146.3, 142.1, 130.1, 128.5, 128.4, 126.0, 59.4 (d, *J* = 12.0 Hz), 36.2, 33.1, 30.8, 21.9, 17.8.
^{19}F NMR (375 MHz, CDCl$_3$): δ = -45.87.
IR ν (cm^{-1}): 2965, 2926 1596.
HRMS: Mass calculated for $C_{18}H_{22}FNNaO_2S$: 358.1247, found: 358.1242.

_N_-fluoro-4-methyl-_N_-(5-phenylpentyl)benzenesulfonamide (1d)

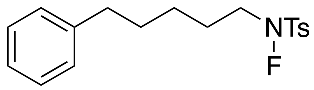

1d was obtained as a yellow oil (83%) by applying **GP 1**.
^{1}H NMR (300 MHz, CDCl$_3$): δ = 7.82 (d, *J* = 8.3 Hz, 2H), 7.40 (d, *J* = 7.9 Hz, 2H), 7.31-7.24 (m, 2H), 7.21-7.13 (m, 3H), 3.20 (dt, *J* = 40.7, 7.0 Hz, 2H), 2.61 (t, *J* = 7.6 Hz, 2H), 2.48 (s, 3H), 1.80-1.58 (m, 4H), 1.51-1.39 (m, 2H).
^{13}C{^{1}H} NMR (100 MHz, CDCl$_3$): δ = 146.3, 142.4, 130.1, 130.1, 129.1, 128.5, 128.5, 125.9, 53.7 (d, *J* = 10.6 Hz), 35.8, 31.0, 26.3, 21.9.
^{19}F NMR (375 MHz, CDCl$_3$): δ = -49.54.
IR ν (cm^{-1}): 2928, 2858, 1596.
HRMS: Mass calculated for $C_{18}H_{22}FNNaO_2S$: 358.1247, found: 358.1241.

_N_-Fluoro-4-methyl-_N_-(4-phenylbutyl-4-_d_)benzenesulfonamide (1b-d1)

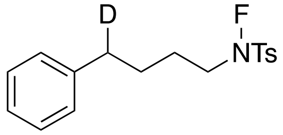

1b-d1 was obtained as a white solid (60%) by applying **GP 3**.
^{1}H NMR (400 MHz, CDCl$_3$): δ = 7.82 (d, *J* = 8.7 Hz, 2H), 7.40 (d, *J* = 8.8 Hz, 2H), 7.29-7.25 (m, 2H), 7.21-7.14 (m, 3H), 3.31-3.14 (m, 2H), 2.65-2.59 (m, 1H), 2.48 (s, 3H), 1.79-1.72 (m, 4H).
^{13}C{^{1}H} NMR (100 MHz, CDCl$_3$): δ = 146.3, 141.8, 130.1, 130.1, 128.5, 126.0, 53.6 (d, *J* = 12.4 Hz), 35.6-34.7 (m), 28.4, 26.0, 21.9.
^{19}F NMR (375 MHz, CDCl$_3$): δ = -49.79.
IR ν (cm^{-1}): 2917, 2862, 1593.
mp: 58-59 °C.

HRMS: Mass calculated for $C_{17}H_{19}DFNNaO_2S$: 345.1154, found: 345.1156.

***N*-fluoro-4-methyl-*N*-(4-phenylbutyl-4,4-*d*$_2$)benzenesulfonamide (1b-d2)**

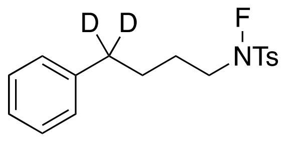

1b-d2 was obtained as a red oil (50%) by applying **GP 3**.
^{1}H NMR (400 MHz, CDCl$_3$): δ = 7.85-7.80 (m, 2H), 7.44-7.39 (m, 2H), 7.32-7.26 (m, 2H), 7.22-7.13 (m, 3H), 3.31-3.16 (m, 2H), 2.49 (s, 3H), 1.80-1.71 (m, 4H).
^{13}C{^{1}H} NMR (100 MHz, CDCl$_3$): δ = 146.3, 141.7, 130.0, 130.0, 128.4, 126.9, 53.7 (d, *J* = 12.4 Hz), 35.2-34.1 (m), 28.2, 25.9, 21.8.
^{19}F NMR (375 MHz, CDCl$_3$): δ = -49.77.
IR ν (cm^{-1}): 2961, 2951, 2939, 2910, 2873,1593.
mp: 68-69 °C.
HRMS: Mass calculated for $C_{17}H_{18}D_2FNNaO_2S$: 346.1222, found: 346.1218.

***N*-fluoro-4-methyl-*N*-(5-phenylpentyl-5-*d*)benzenesulfonamide (1d-d1)**

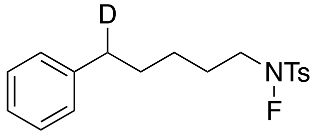

1d-d1 was obtained as a colorless oil (41%) by applying **GP 3**.
^{1}H NMR (400 MHz, CDCl$_3$): δ = 7.85-7.79 (m, 2H), 7.44-7.38 (m, 2H), 7.30-7.25 (m, 2H), 7.21-7.13 (m, 3H), 3.20 (dt, *J* = 40.6, 7.0 Hz, 2H), 2.59 (t, *J* = 7.7 Hz, 1H), 2.48 (s, 3H), 1.74 (p, *J* = 7.2 Hz, 2H), 1.64 (q, *J* = 7.6 Hz, 2H), 1.48-1.40 (m, 2H).
^{13}C{^{1}H} NMR (100 MHz, CDCl$_3$): δ = 146.3, 142.4, 130.1, 130.1, 128.5, 128.4, 125.9, 53.7 (d, *J* = 12.5 Hz), 35.7-35.1 (m), 31.0, 26.4, 26.3, 21.9.
^{19}F NMR (375 MHz, CDCl$_3$): δ = -49.91.
IR ν (cm^{-1}): 3026, 2926, 2860, 1596.
HRMS: Mass calculated for $C_{18}H_{21}DFNNaO_2S$: 359.1310, found: 359.1310.

***N*-fluoro-4-methyl-*N*-(5-phenylpentyl-5,5-*d*$_2$)benzenesulfonamide (1d-d2)**

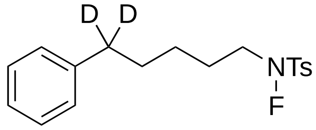

1d-d2 was obtained as a colorless oil (55%) by applying **GP 3**.

^{1}**H NMR (400 MHz, CDCl$_3$):** δ = 7.82 (d, *J* = 8.3 Hz, 2H), 7.43-7.38 (m, 2H), 7.31-7.25 (m, 2H), 7.21-7.14 (m, 3H), 3.21 (dt, *J* = 40.7, 7.0 Hz, 2H), 2.48 (s, 3H), 1.80-1.70 (m, 2H), 1.67-1.60 (m, 2H), 1.49-1.39 (m, 2H).
^{13}**C{^{1}H} NMR (100 MHz, CDCl$_3$):** δ = 146.3, 142.3, 130.1, 130.1, 129.0, 128.5, 128.4, 125.9, 53.7 (d, *J* = 12.4 Hz), 35.5-34.6 (m), 30.9, 26.3, 26.3, 21.9.
^{19}**F NMR (375 MHz, CDCl$_3$):** δ = -49.89.
IR ν (cm^{-1}): 3024, 2925, 2859, 1596.
HRMS: Mass calculated for $C_{18}H_{20}D_2FNNaO_2S$: 360.1373, found: 360.1385.

***N*-(4-fluoro-4-phenylbutyl)-4-methylbenzenesulfonamide (5)**

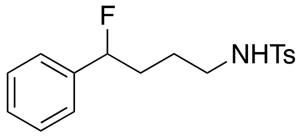

5 was obtained as a yellow solid (79%) by applying **GP 4**.
Yellow solid
^{1}**H NMR (400 MHz, CDCl$_3$):** δ = 7.77-7.71 (m, 2H), 7.38-7.23 (m, 7H), 5.38 (ddd, *J* = 47.8, 8.1, 4.5 Hz, 1H), 4.75 (t, *J* = 6.3 Hz, 1H), 3.03-2.93 (m, 2H), 2.42 (s, 3H), 1.99-1.75 (m, 2H), 1.71-1.52 (m, 2H).
^{13}**C{^{1}H} NMR (75 MHz, CDCl$_3$):** δ = 143.6, 140.1, 139.8, 137.0, 129.8, 128.6, 128.4, 128.4, 127.2, 125.5, 125.4, 94.0 (d, *J* = 170.9 Hz), 42.9, 34.1 (d, *J* = 23.9 Hz), 25.4 (d, *J* = 3.8 Hz), 21.6.
^{19}**F NMR (375 MHz, CDCl$_3$):** δ = -175.90.
IR ν (cm^{-1}): 3279, 2926,1598.
mp: 71-72 °C.
HRMS: Mass calculated for $C_{17}H_{20}FNNaO_2S$: 344.1091, found: 344.1081.

***N*-chloro-*N*-(2,2-dimethyl-4-phenylbutyl)-4-methylbenzenesulfonamide (6)**

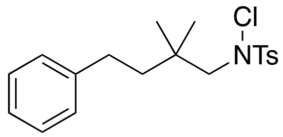

6 was obtained as a colorless oil (50%) by applying **GP 5**.
^{1}**H NMR (400 MHz, CDCl$_3$):** δ = 7.89-7.81 (m, 2H), 7.43-7.36 (m, 2H), 7.32-7.25 (m, 2H), 7.23-7.15 (m, 3H), 3.24 (s, 2H), 2.67-2.58 (m, 2H), 2.48 (s, 3H), 1.73-1.64 (m, 2H), 1.10 (s, 6H).
^{13}**C{^{1}H} NMR (100 MHz, CDCl$_3$):** δ = 145.3, 142.8, 131.0, 129.8, 129.5, 128.5, 128.4, 125.8, 67.0, 42.1, 35.7, 30.4, 27.5, 25.7, 21.8.
IR ν (cm^{-1}): 2961, 2930, 1597.
HRMS: Mass calculated for $C_{19}H_{24}ClNNaO_2S$: 388.1108, found: 388.1098.

***N*-(4-chloro-2,2-dimethyl-4-phenylbutyl)-4-methylbenzenesulfonamide (7)**

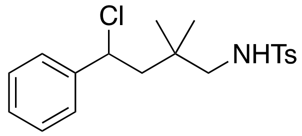

7 was obtained as a white solid as described in section **XIV**.

^{1}H NMR (500 MHz, CD_2Cl_2): δ = 7.60-7.54 (m, 2H), 7.34-7.21 (m, 7H), 4.61 (dd, *J* = 9.5, 7.2 Hz, 1H), 3.36 (s, 2H), 2.41 (s, 3H), 2.04-1.93 (m, 1H), 1.74-1.63 (m, 1H), 1.03 (s, 3H), 0.63 (s, 3H). NH is not observed.

^{13}C{^{1}H} NMR (125 MHz, CD_2Cl_2): δ = 144.1, 144.0, 135.8, 130.0, 128.8, 128.0, 127.5, 127.0, 64.4, 62.5, 52.1, 38.5, 26.3, 25.8, 21.8.

IR ν (cm^{-1}): 2962, 2923, 2898, 2879, 1599.

mp: 82-83 °C.

HRMS: Mass calculated for $C_{19}H_{24}ClNNaO_2S$: 388.1108, found: 388.1111.

XV. Analytical data of saturated *N*-heterocycles

4,4-Dimethyl-2-phenyl-1-tosylpyrrolidine (4a)

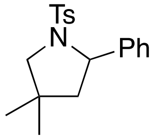

4a was obtained as a white solid (82%) by applying **GP 2**. The NMR data match those reported in literature.[5]

^{1}H NMR (400 MHz, CDCl$_3$): δ = 7.55-7.51 (m, 2H), 7.27-7.25 (m, 4H), 7.24-7.19 (m, 3H), 4.71 (dd, *J* = 9.4, 7.3 Hz, 1H), 3.45 (dd, *J* = 10.4, 1.5 Hz, 1H), 3.34 (d, *J* = 10.4 Hz, 1H), 2.40 (s, 3H), 2.02 (ddd, *J* = 12.8, 7.3, 1.5 Hz, 1H), 1.73 (dd, *J* = 12.8, 9.4 Hz, 1H), 1.05 (s, 3H), 0.77 (s, 3H).

^{13}C{^{1}H} NMR (100 MHz, CDCl$_3$): δ = 143.1, 143.0, 136.0, 129.4, 128.4, 127.5, 127.2, 126.6, 64.0, 62.0, 51.7, 38.3, 26.2, 25.8, 21.6.

2-Phenyl-1-tosylpyrrolidine (4b)

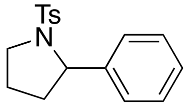

4b was obtained as a white solid (71%) by applying **GP 2**. The NMR data match those reported in literature.[5]

^{1}H NMR (300 MHz, CDCl$_3$): δ = 7.68 (d, *J* = 8.3 Hz, 2H), 7.34-7.20 (m, 7H), 4.80 (dd, *J* = 7.8, 3.7 Hz, 1H), 3.68-3.58 (m, 1H), 3.49-3.38 (m, 1H), 2.43 (s, 3H), 2.07-1.93 (m, 1H), 1.91-1.76 (m, 2H), 1.74-1.59 (m, 1H).

^{13}C{^{1}H} NMR (75 MHz, CDCl$_3$): δ = 143.4, 143.2, 135.3, 129.7, 128.4, 127.6, 127.1, 126.3, 63.4, 49.5, 35.9, 24.1, 21.6.

4-methyl-2-phenyl-1-tosylpyrrolidine (4c)

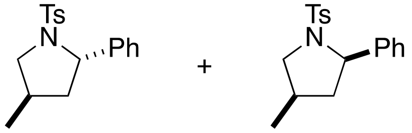

4c was obtained as a colorless oil (84%) in a 1:1 diastereomeric mixture by applying **GP 2**. The NMR data match those reported in literature (trans diastereoisomer described).[5]

^{1}H NMR (400 MHz, CDCl$_3$): δ = 7.62-7.58 (m, 2H), 7.32-7.27 (m, 6H), 7.25-7.19 (m, 1H), 4.64 (dd, *J* = 9.5, 7.2 Hz, 1H), 3.84 (ddd, *J* = 11.2, 7.3, 1.4 Hz, 1H), 3.09 (t, *J* = 10.8 Hz, 1H), 2.43 (s, 3H), 2.40-2.29 (m, 1H), 1.93-1.79 (m, 1H), 1.64-1.40 (m, 1H), 0.95 (d, *J* = 6.5 Hz, 3H). **^{13}C{^{1}H} NMR (100 MHz, CDCl$_3$)**: δ = 143.4, 143.3, 135.8, 129.6, 128.5, 127.7, 127.2, 126.5, 64.8, 56.8, 45.8, 33.5, 21.7, 16.6.

2-phenyl-1-tosylpiperidine (4d)

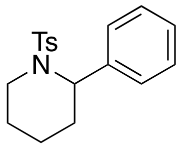

4d was obtained as a white solid (38%) by applying **GP 2**. The NMR data match those reported in literature.[8]

^{1}H NMR (400 MHz, $CDCl_3$): δ = 7.78-7.73 (m, 2H), 7.37-7.27 (m, 7H), 5.27 (d, J = 5.1 Hz, 1H), 3.87-3.80 (m, 1H), 3.07-2.96 (m, 1H), 2.44 (s, 3H), 2.24-2.17 (m, 1H), 1.72-1.58 (m, 1H), 1.50-1.23 (m, 4H).

^{13}C{^{1}H} NMR (100 MHz, $CDCl_3$): δ = 143.1, 139.0, 138.9, 129.8, 128.7, 127.2, 127.1, 126.9, 55.4, 42.0, 27.4, 24.4, 21.7, 19.1.

XVI. NMR Spectra

Figure S7 .^{1}H NMR spectrum of **1a** (400 MHz, $CDCl_3$)

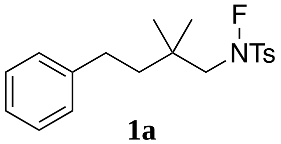

1a

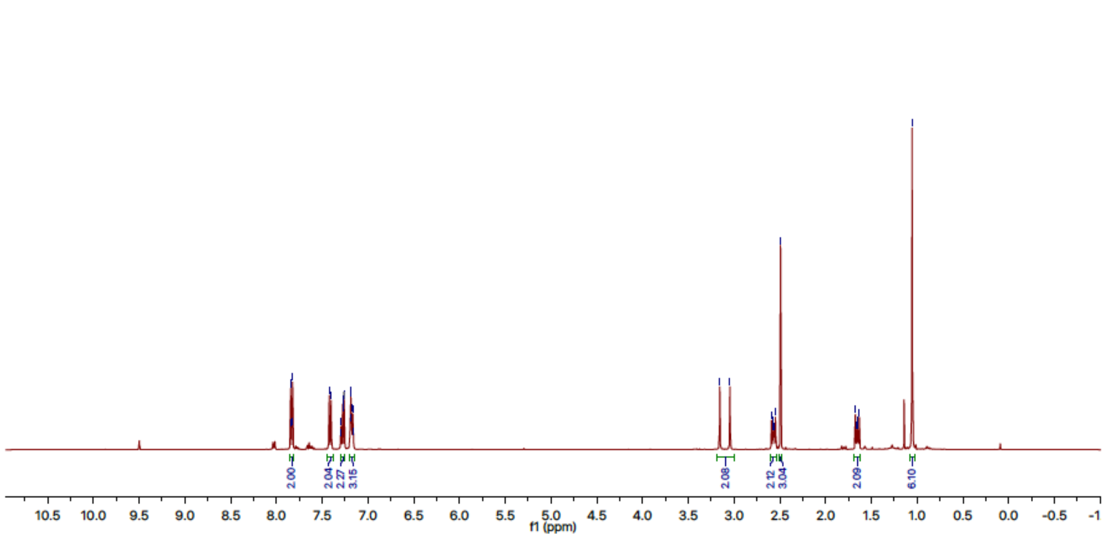

Figure S8. $^{13}C\{^{1}H\}$ NMR spectrum of **1a** (100 MHz, $CDCl_3$)

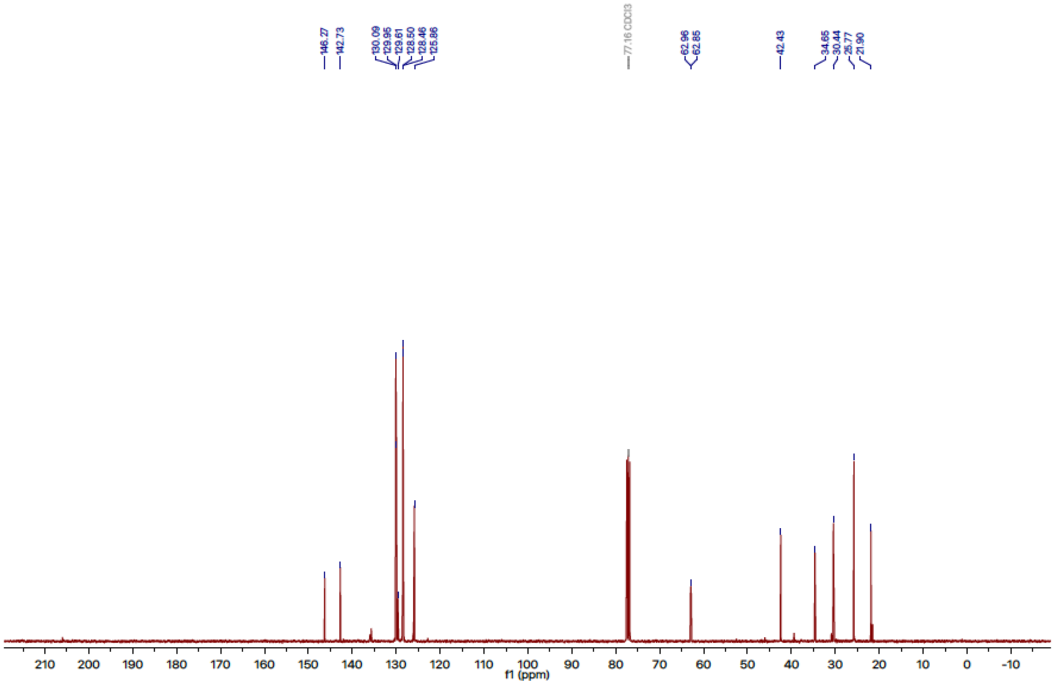

Figure S9. ^{19}F NMR spectrum of **1a** (375 MHz, $CDCl_3$)

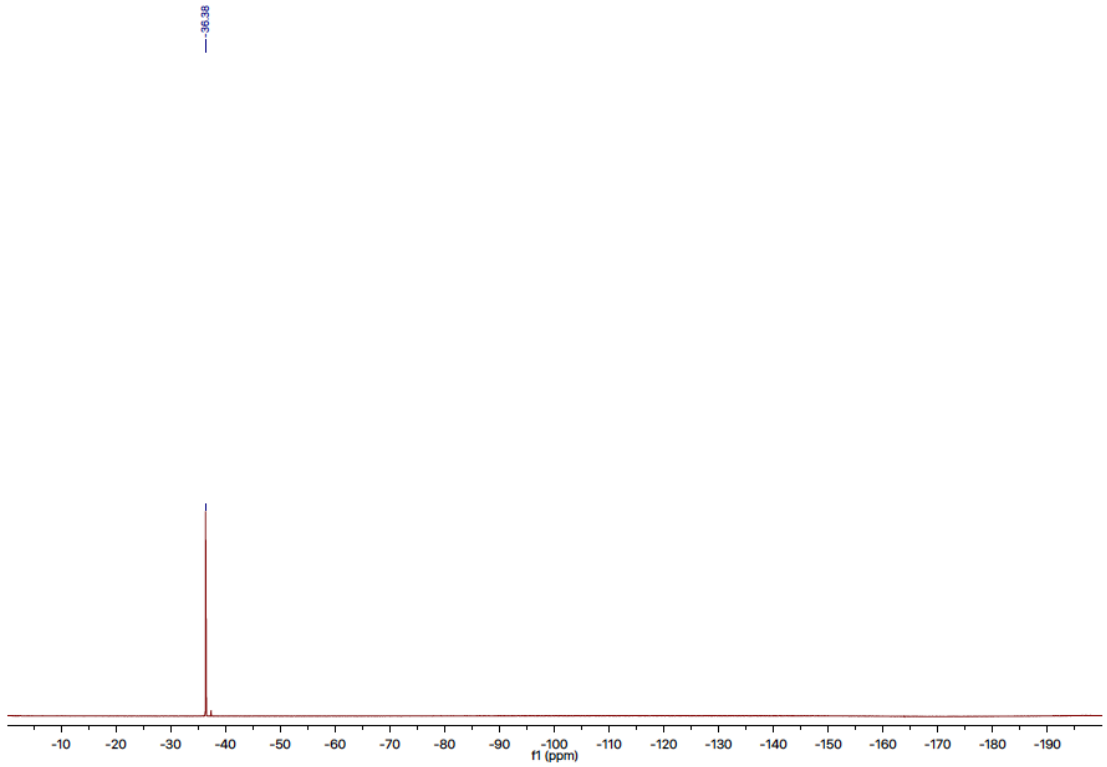

Figure S10. ^{1}H NMR spectrum of **1b** (400 MHz, $CDCl_3$)

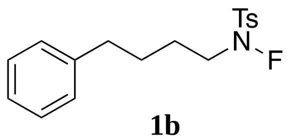

1b

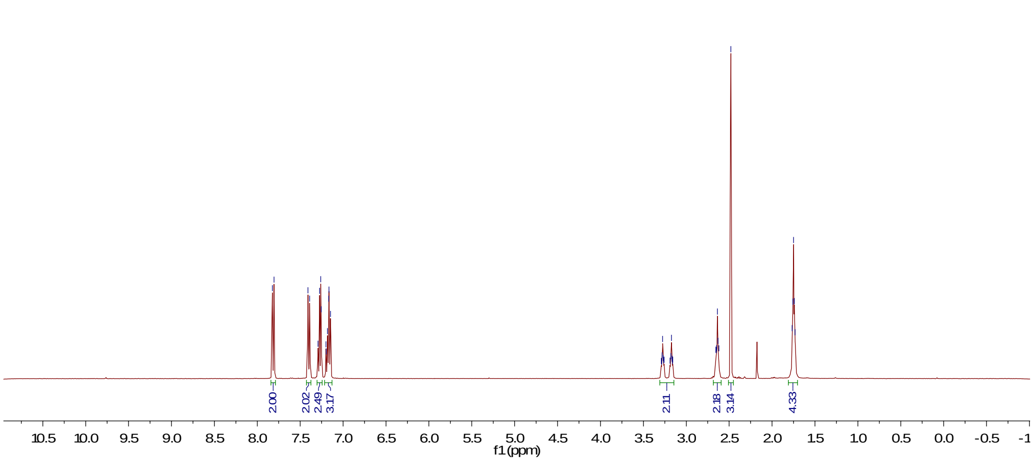

Figure S11. $^{13}C\{^{1}H\}$ NMR spectrum of **1b** (100 MHz, $CDCl_3$)

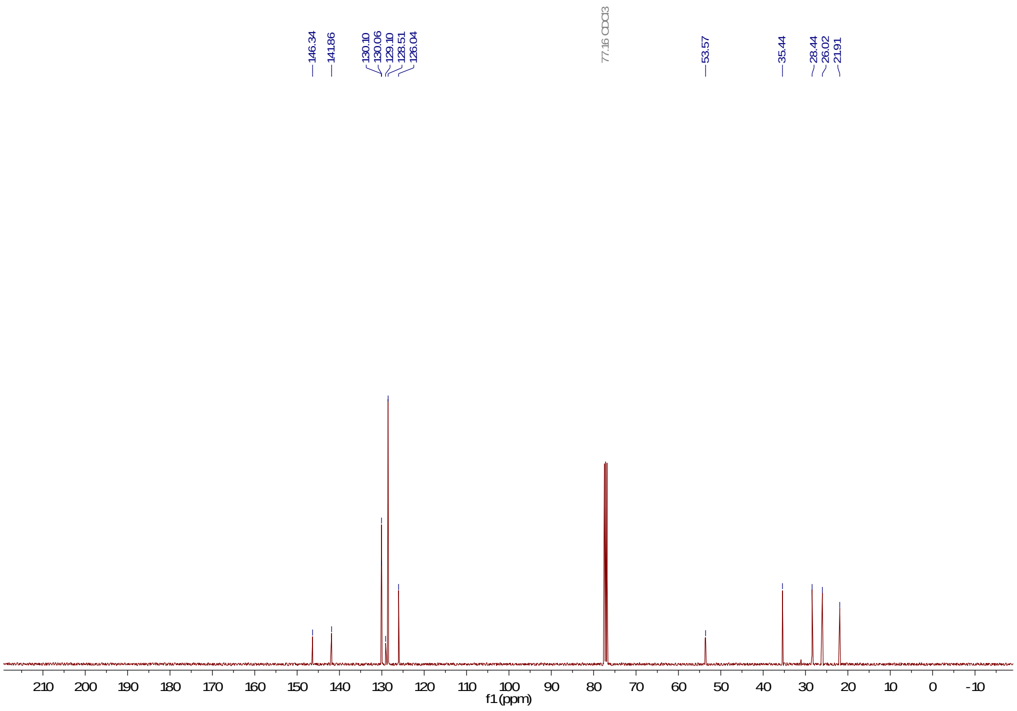

Figure S12. ^{19}F NMR spectrum of **1b** (375 MHz, $CDCl_3$)

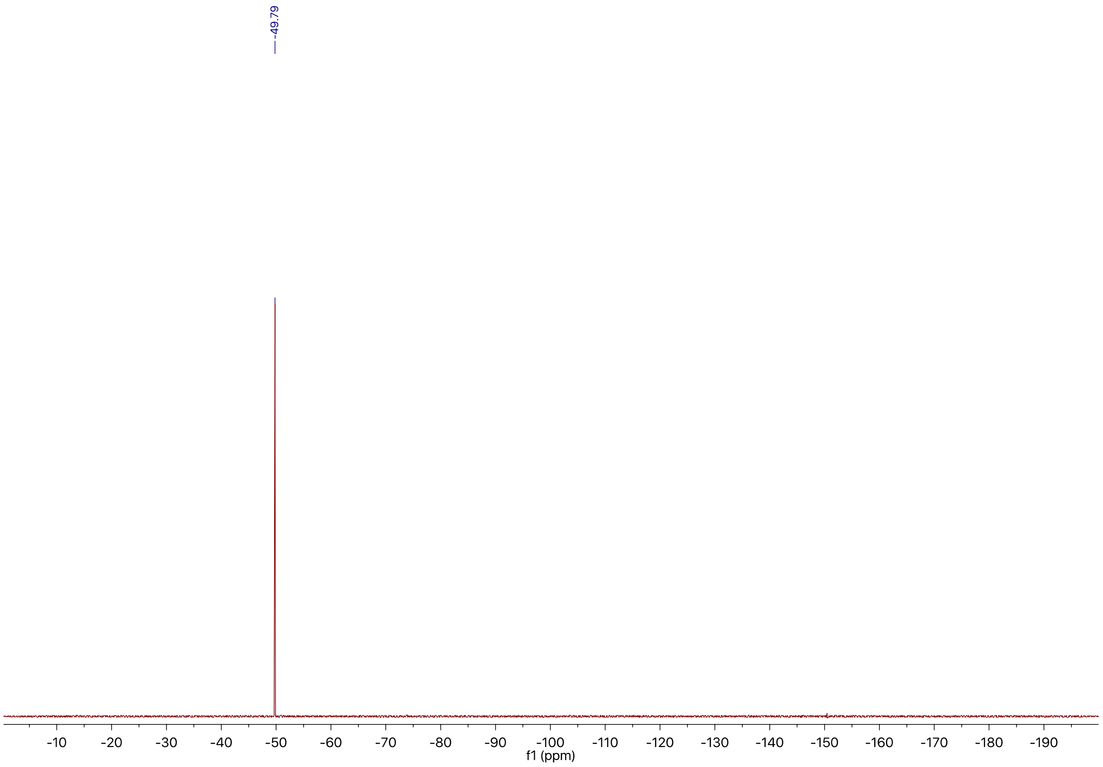

Figure S13. ^{1}H NMR spectrum of **1c** (400 MHz, $CDCl_3$)

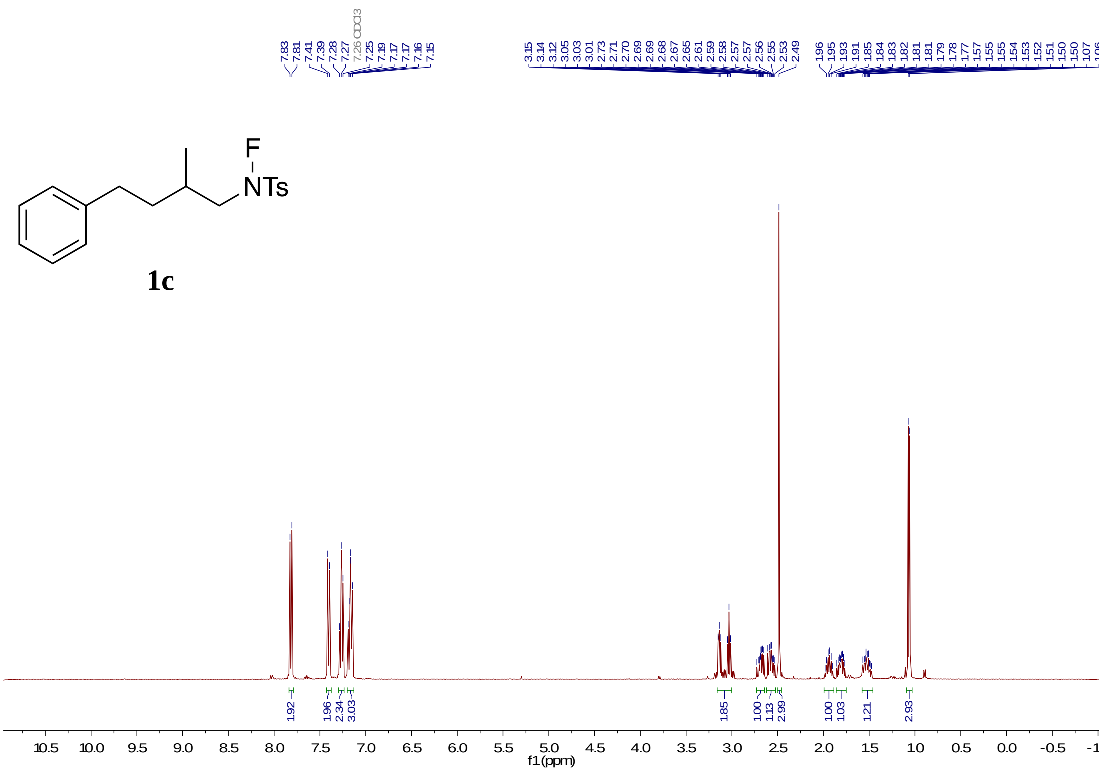

Figure S14. $^{13}C\{^{1}H\}$ NMR spectrum of **1c** (100 MHz, $CDCl_3$)

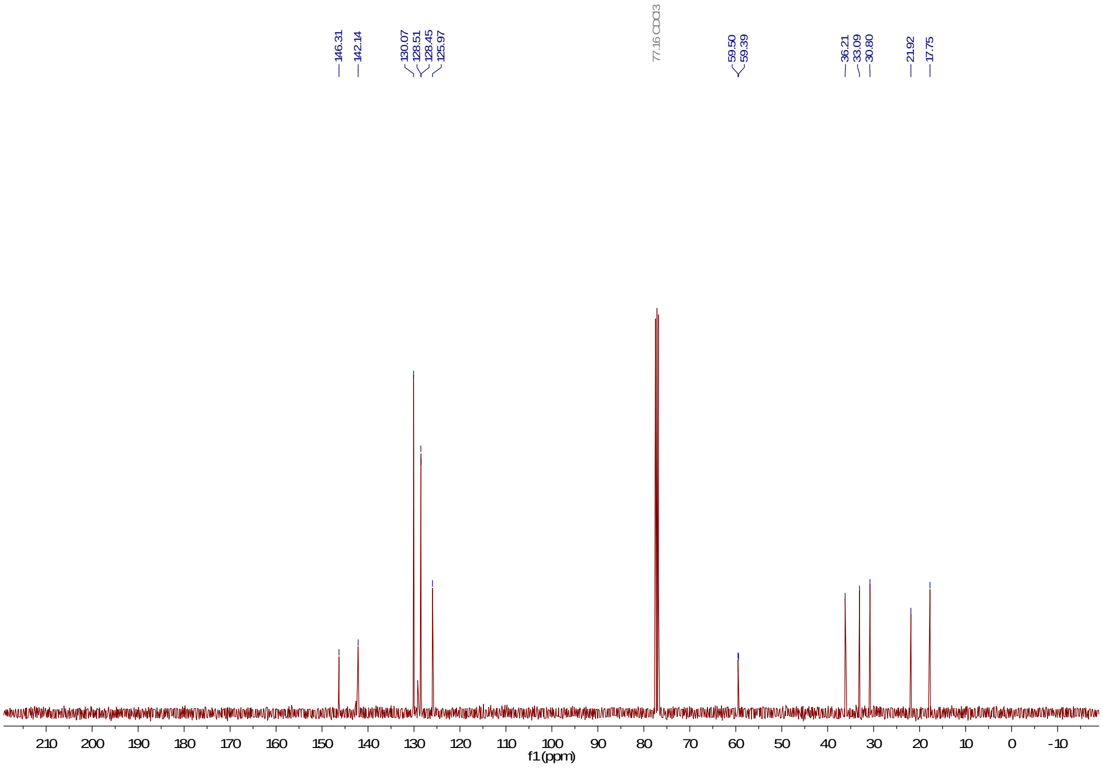

Figure S15. ^{19}F NMR spectrum of **1c** (375 MHz, $CDCl_3$)

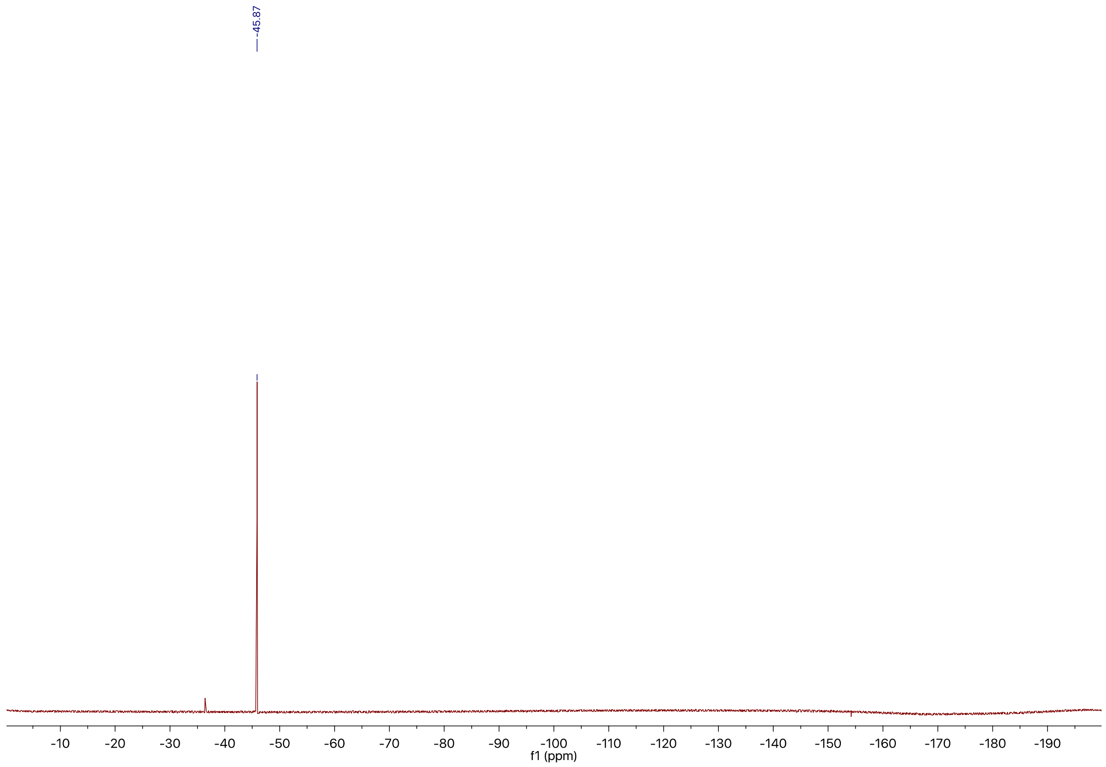

Figure S16. ^{1}H NMR spectrum of **1d** (300 MHz, $CDCl_3$)

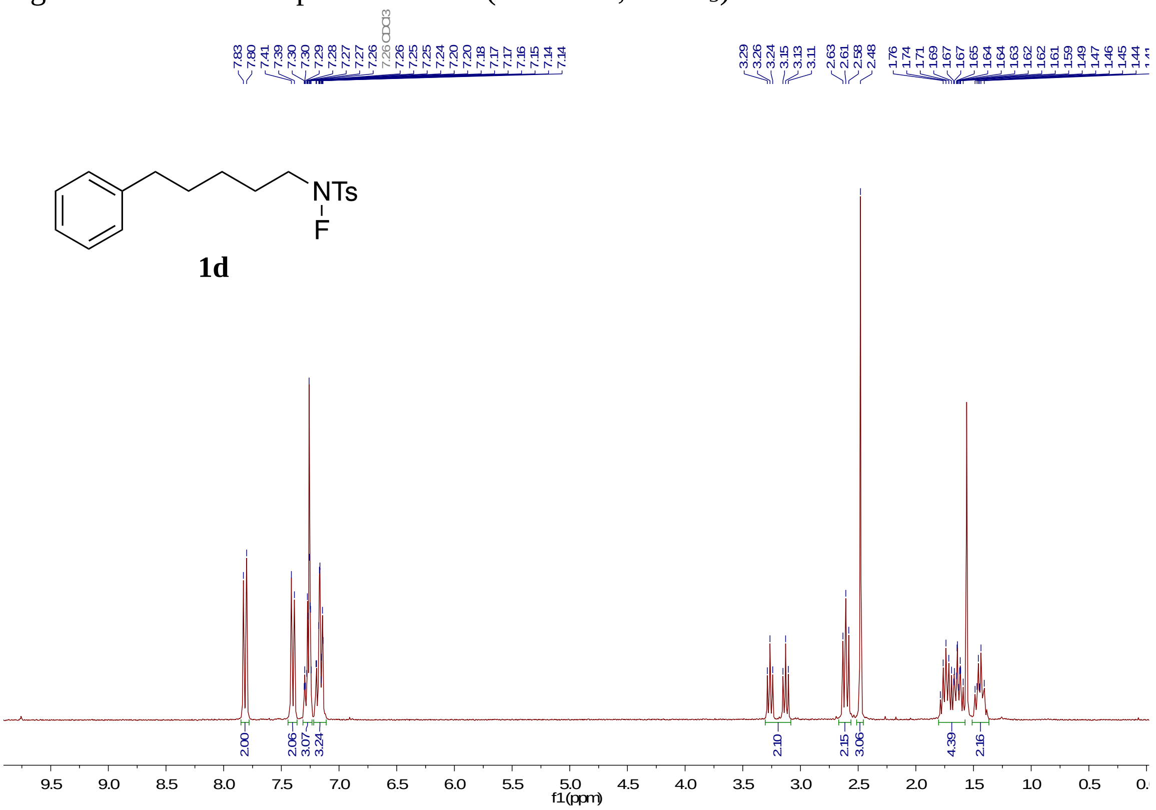

Figure S17. $^{13}C\{^{1}H\}$ NMR spectrum of **1d** (100 MHz, $CDCl_3$)

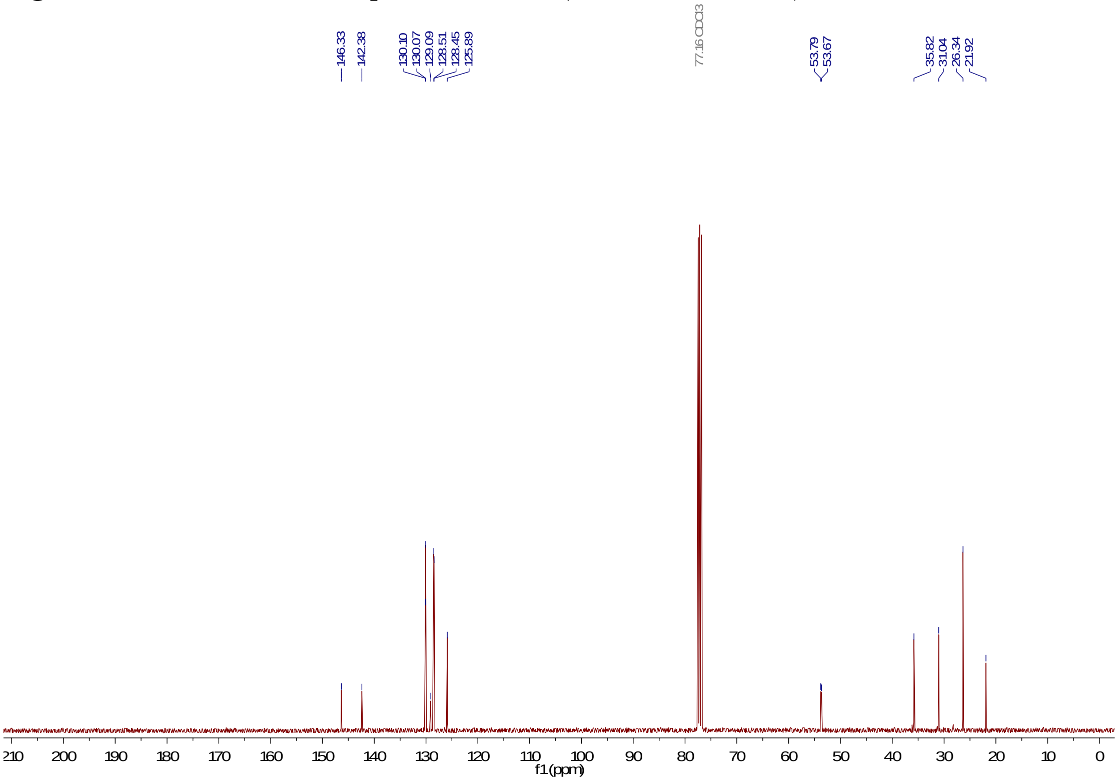

Figure S18. ^{19}F NMR spectrum of **1d** (375 MHz, $CDCl_3$)

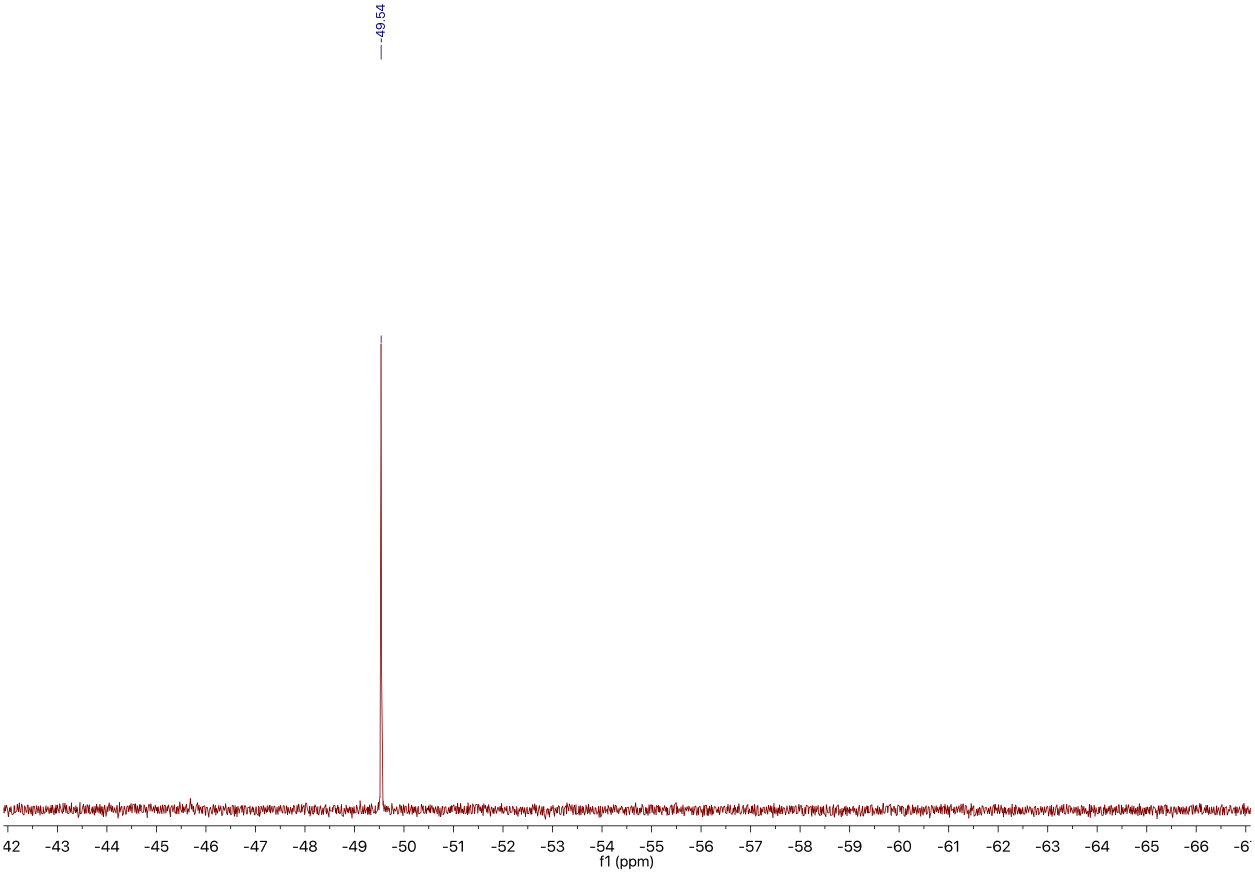

Figure S19. ^{1}H NMR spectrum of **1b-d1** (400 MHz, $CDCl_3$)

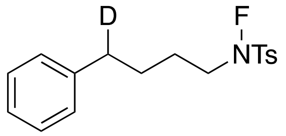

1b-d1

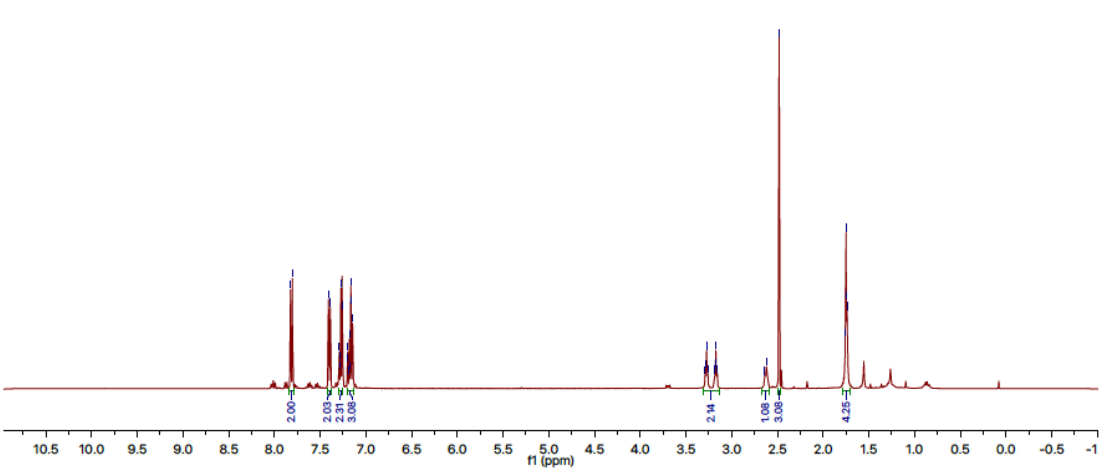

Figure S20. $^{13}C\{^{1}H\}$ NMR spectrum of **1b-d1** (100 MHz, $CDCl_3$)

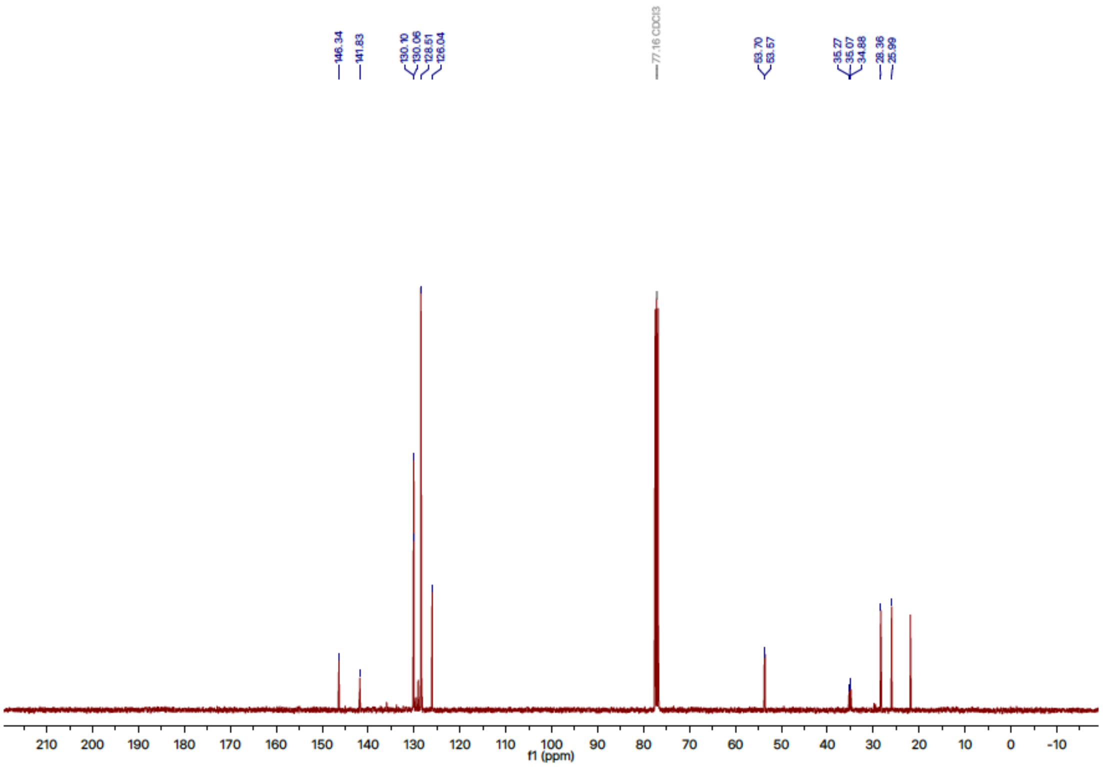

Figure S21. ^{19}F NMR spectrum of **1b-d1** (375 MHz, $CDCl_3$)

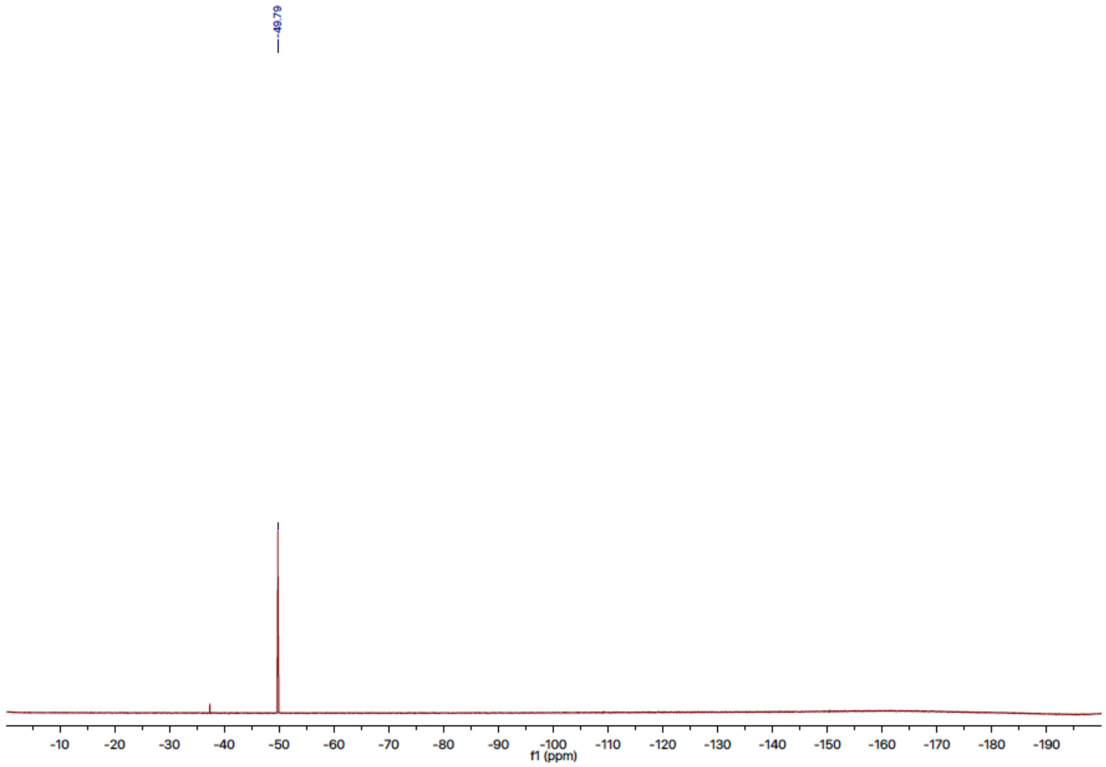

Figure S22. ^{1}H NMR spectrum of **1b-d2** (400 MHz, $CDCl_3$)

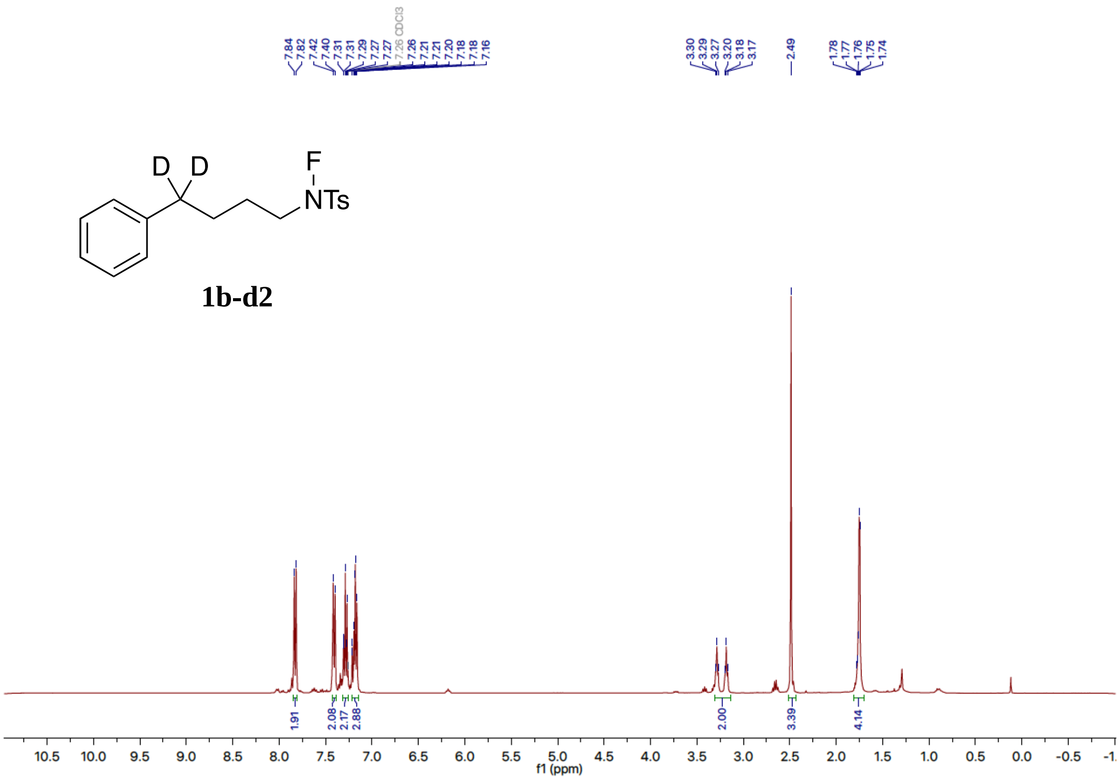

Figure S23. $^{13}C\{^{1}H\}$ NMR spectrum of **1b-d2** (100 MHz, $CDCl_3$)

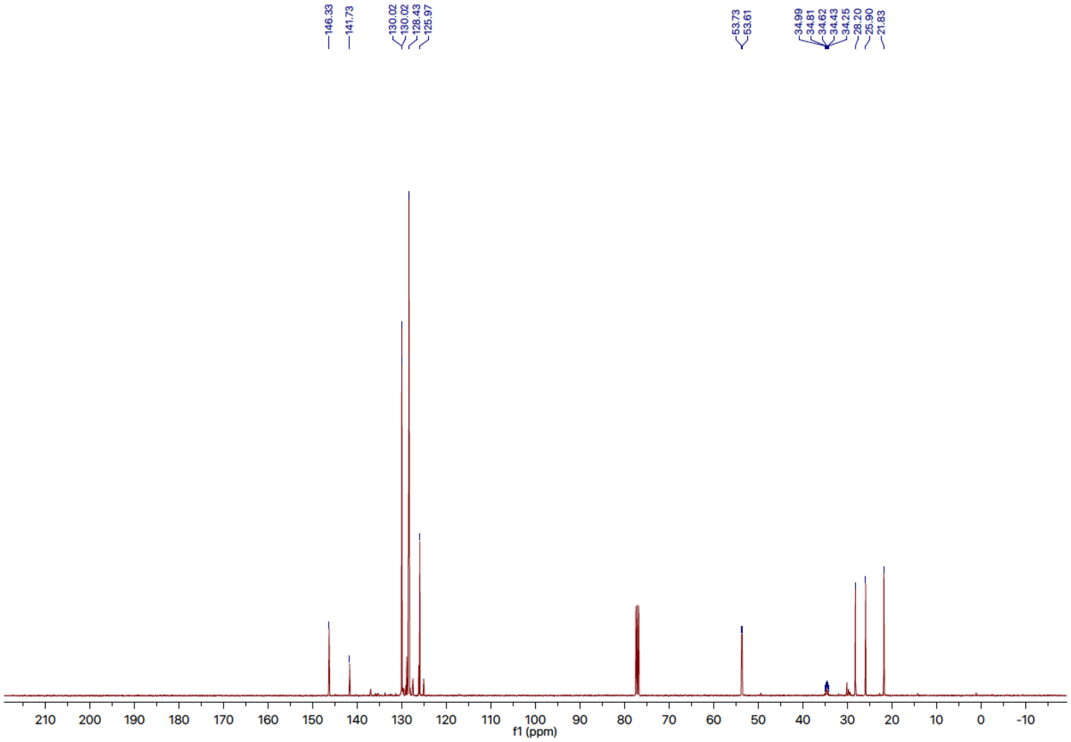

Figure S24. ^{19}F NMR spectrum of **1b-d2** (375 MHz, $CDCl_3$)

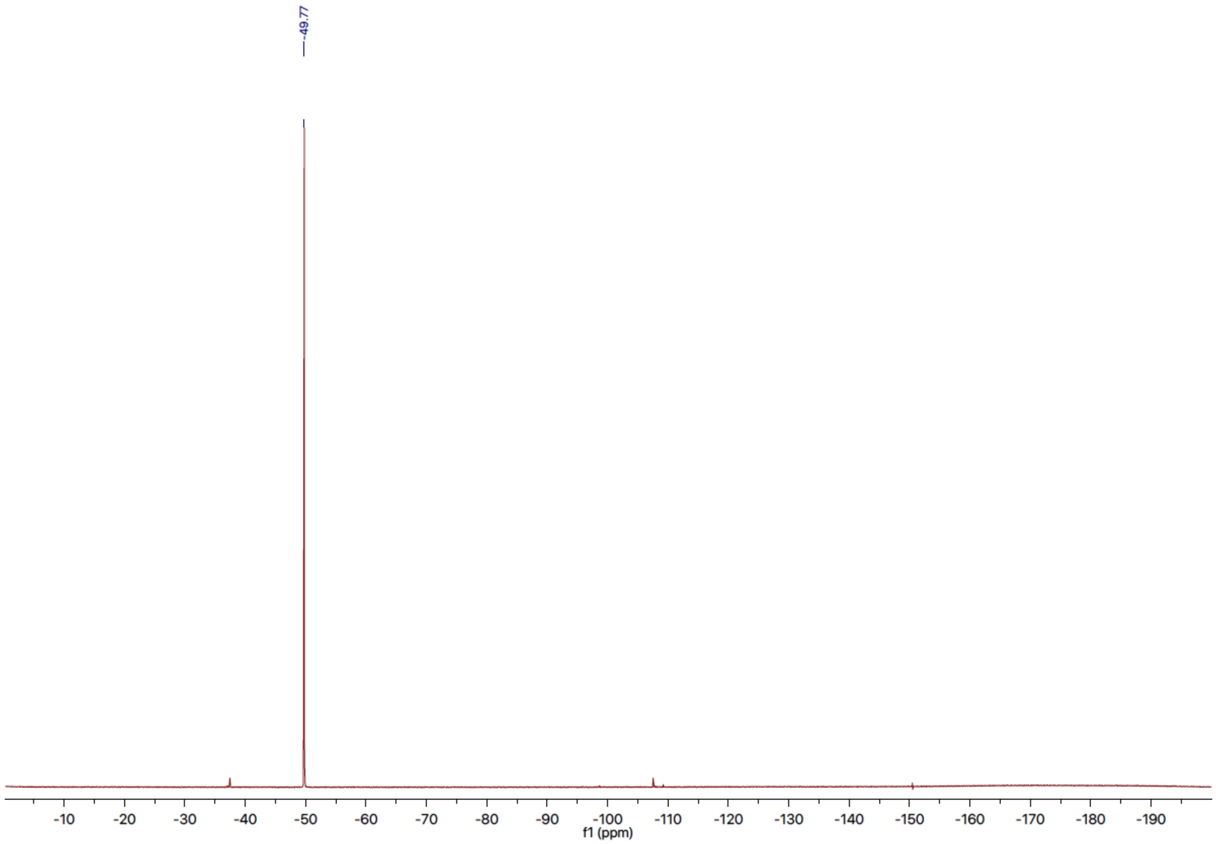

Figure S25. ^{1}H NMR spectrum of **1d-d1** (400 MHz, $CDCl_3$)

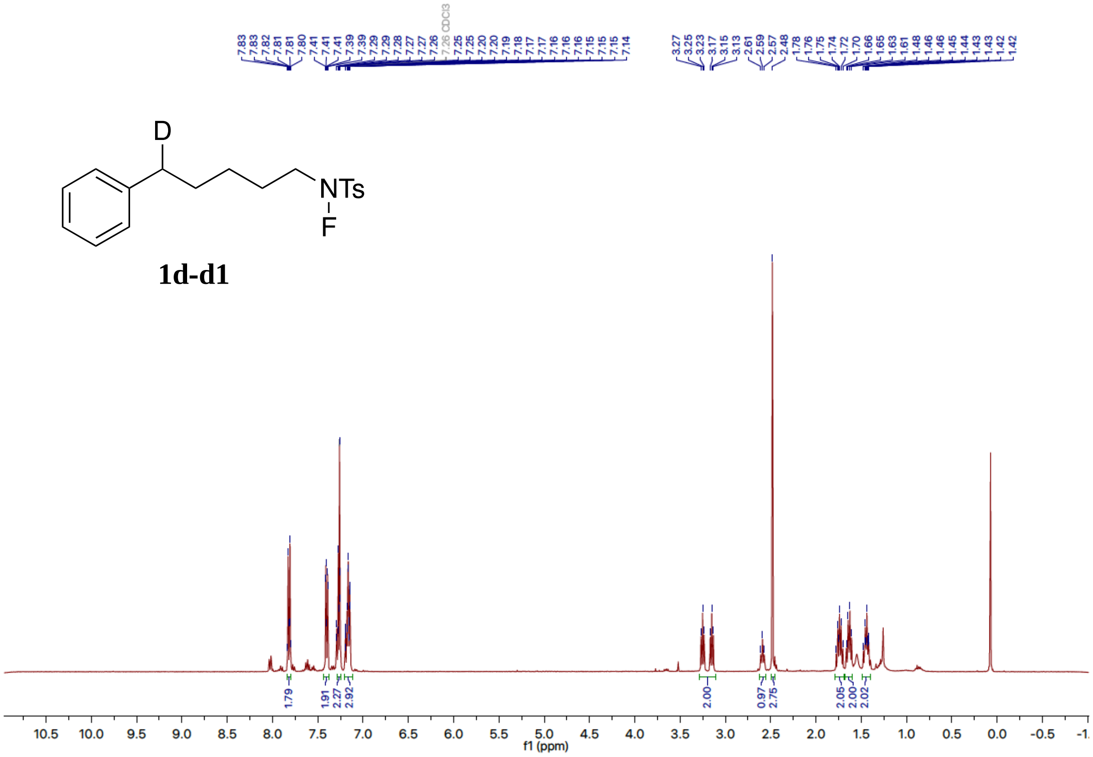

Figure S26. ^{13}C{^{1}H} NMR spectrum of **1d-d1** (100 MHz, $CDCl_3$)

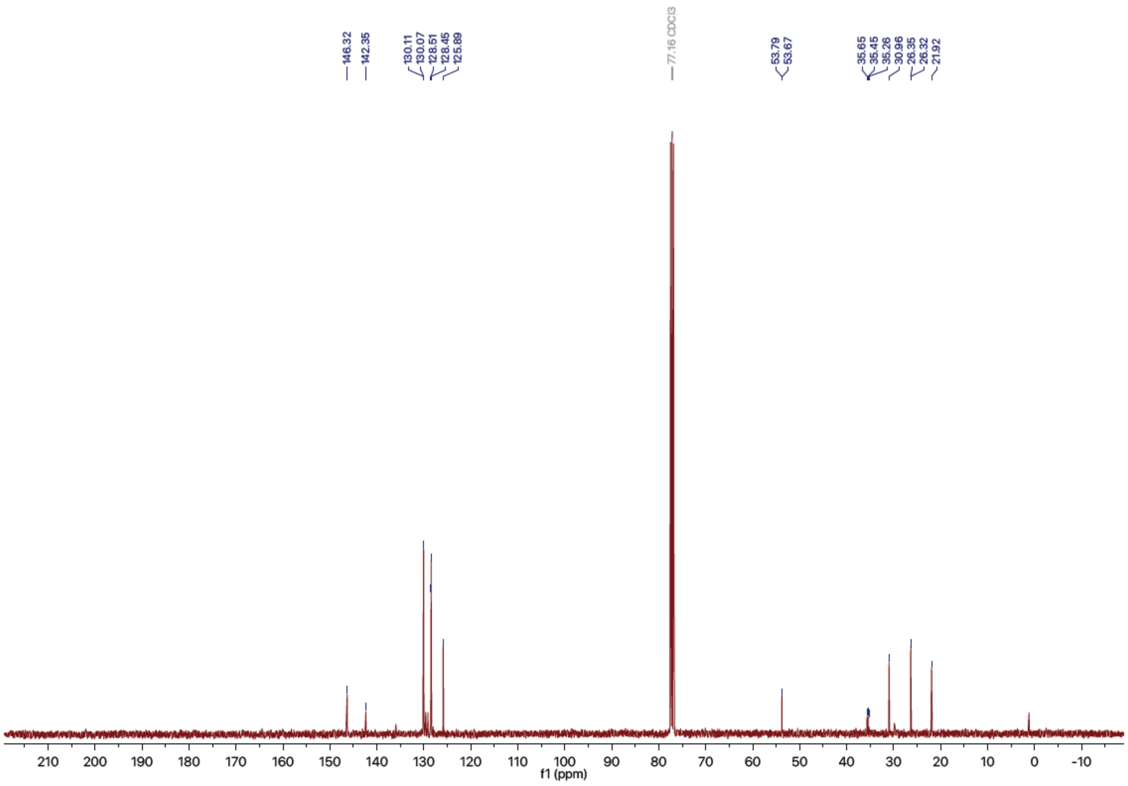

Figure S27. ^{19}F NMR spectrum of **1d-d1** (375 MHz, $CDCl_3$)

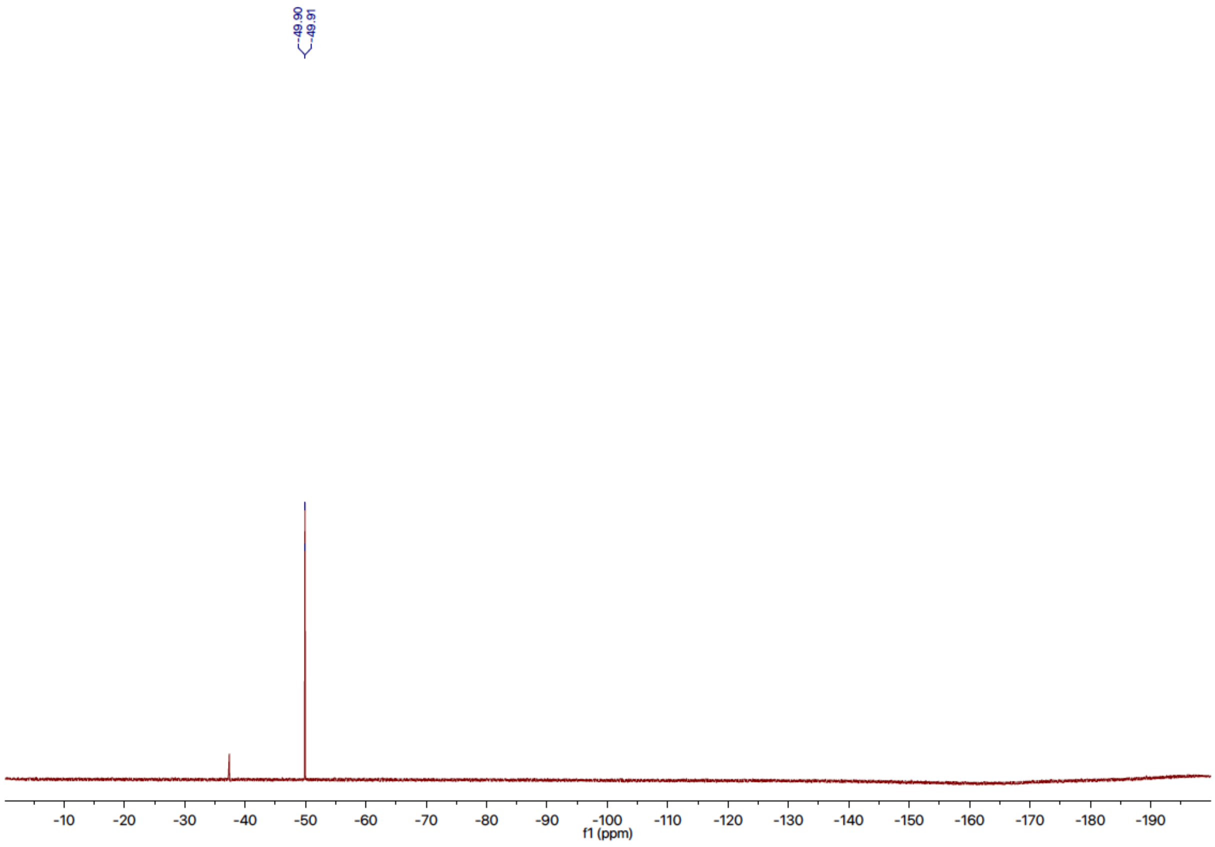

Figure S28. ^{1}H NMR spectrum of **1d-d2** (400 MHz, $CDCl_3$)

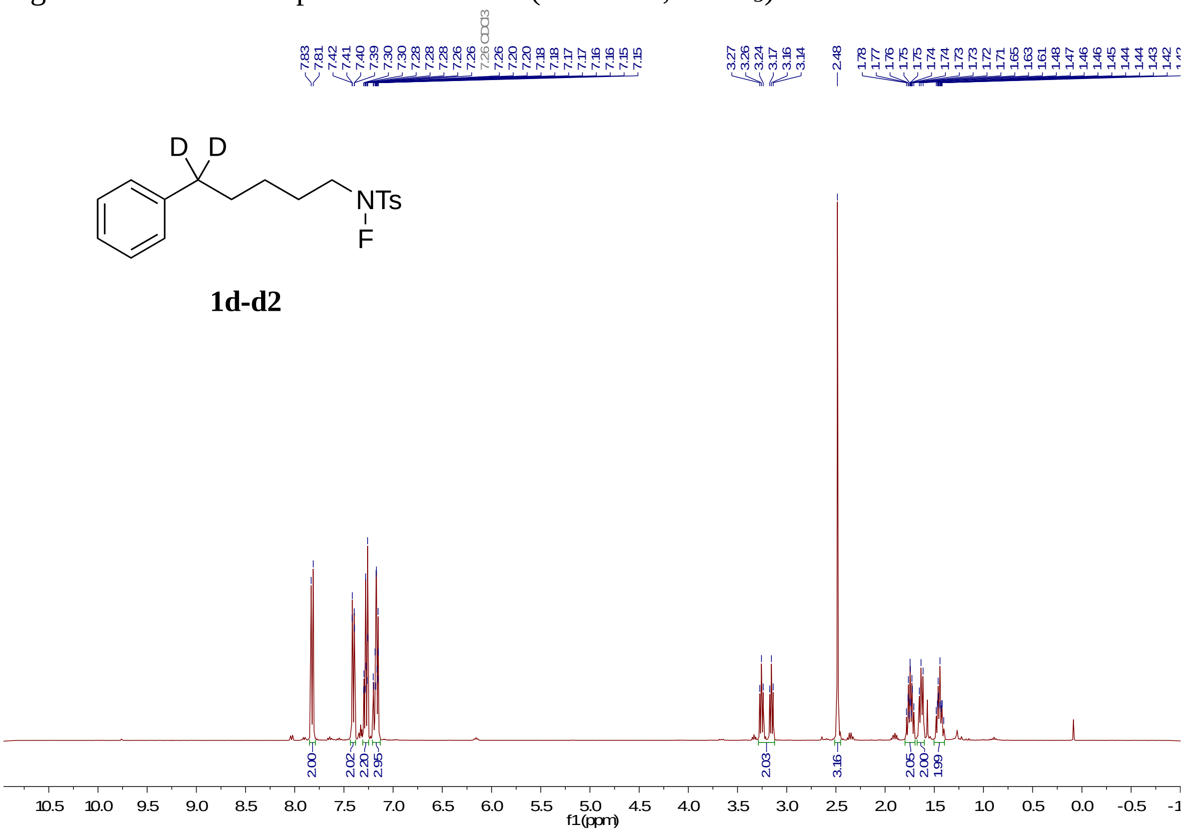

Figure S29. $^{13}C\{^{1}H\}$ NMR spectrum of **1d-d2** (100 MHz, $CDCl_3$)

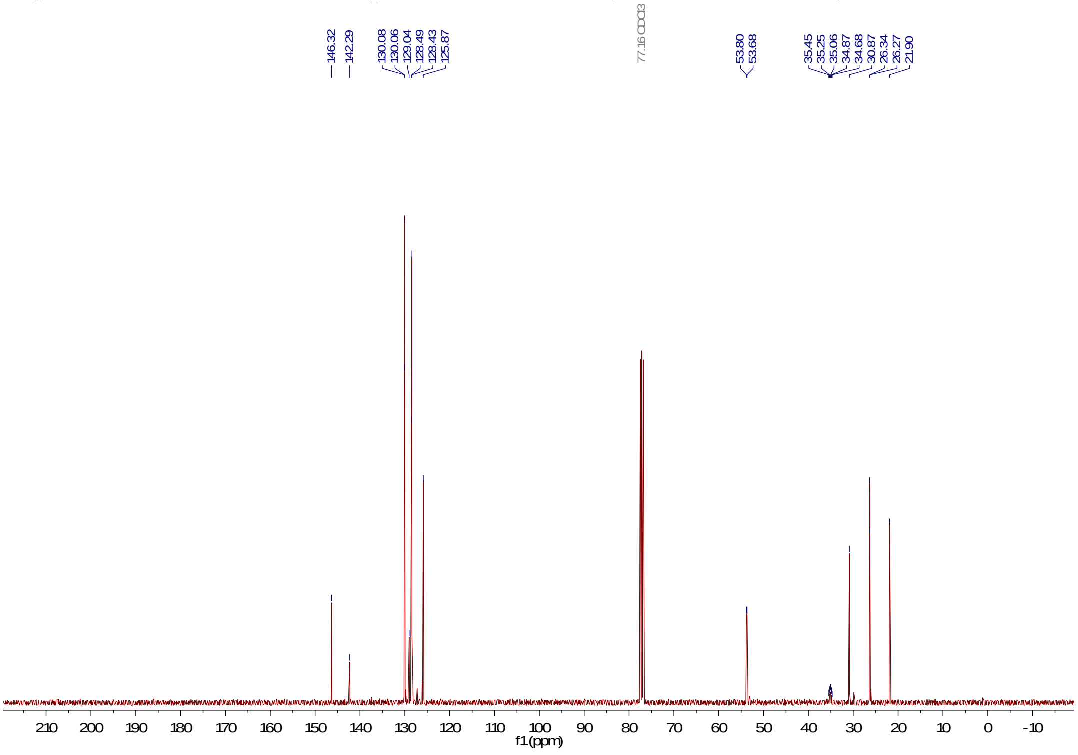

Figure S30. ^{19}F NMR spectrum of **1d-d2** (375 MHz, $CDCl_3$)

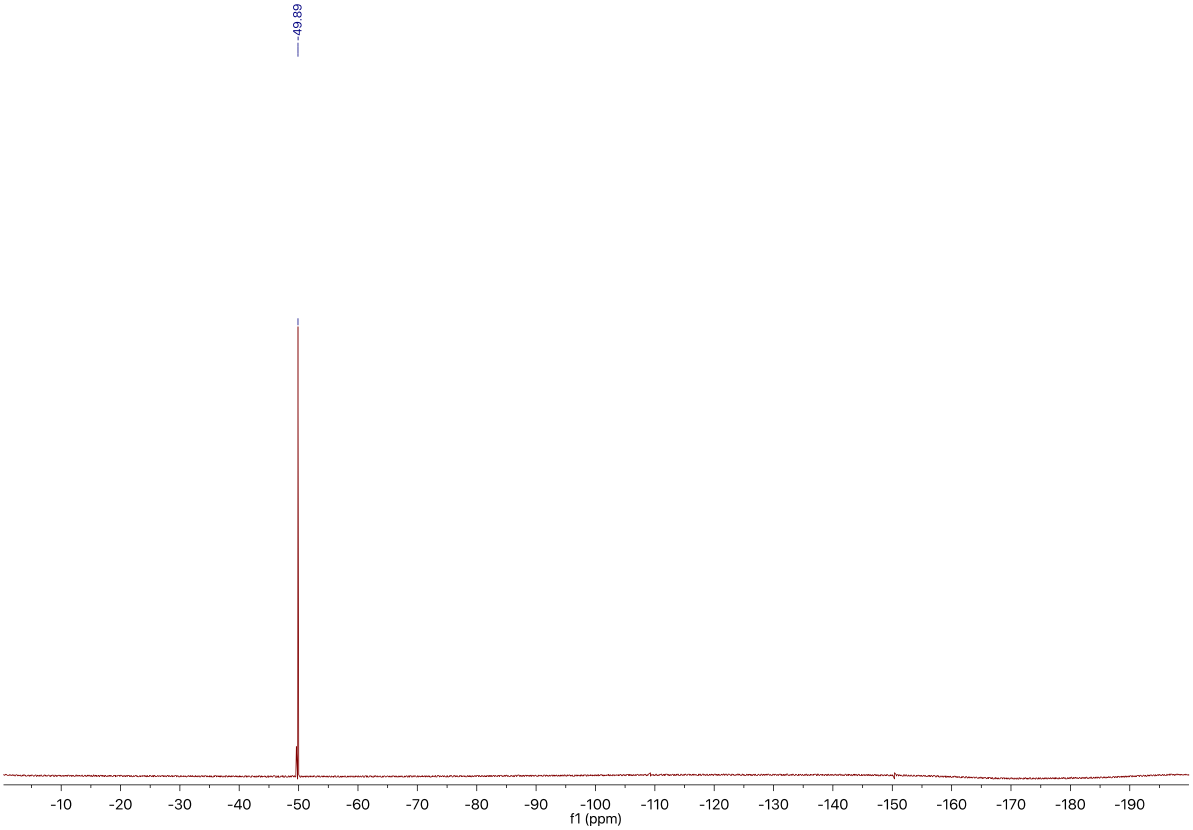

Figure S31. ^{1}H NMR spectrum of **5** (400 MHz, $CDCl_3$)

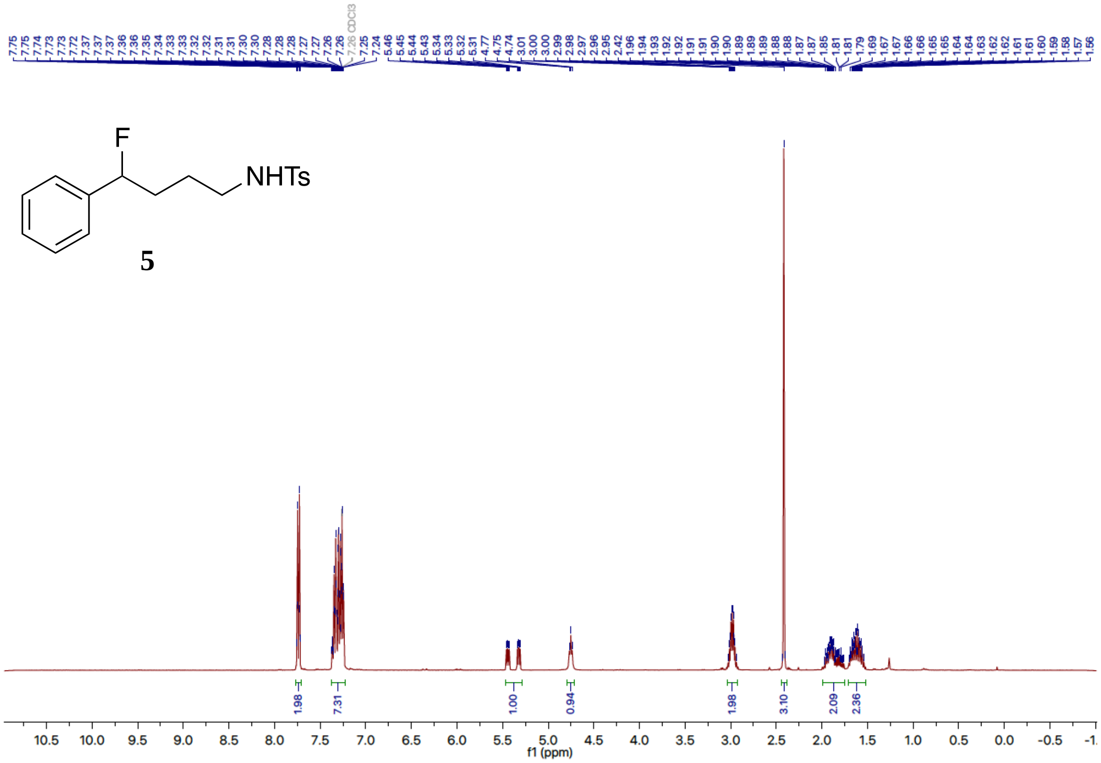

Figure S32. $^{13}C\{^{1}H\}$ NMR spectrum of **5** (75 MHz, $CDCl_3$)

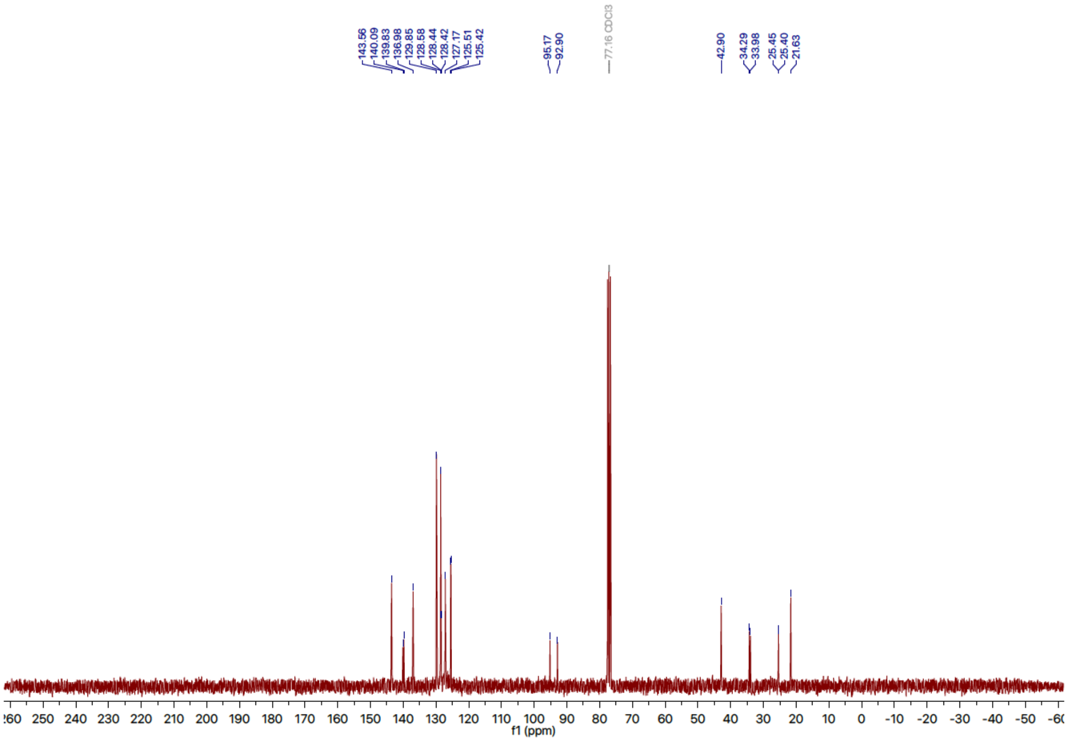

Figure S33. ^{19}F NMR spectrum of **5** (375 MHz, $CDCl_3$)

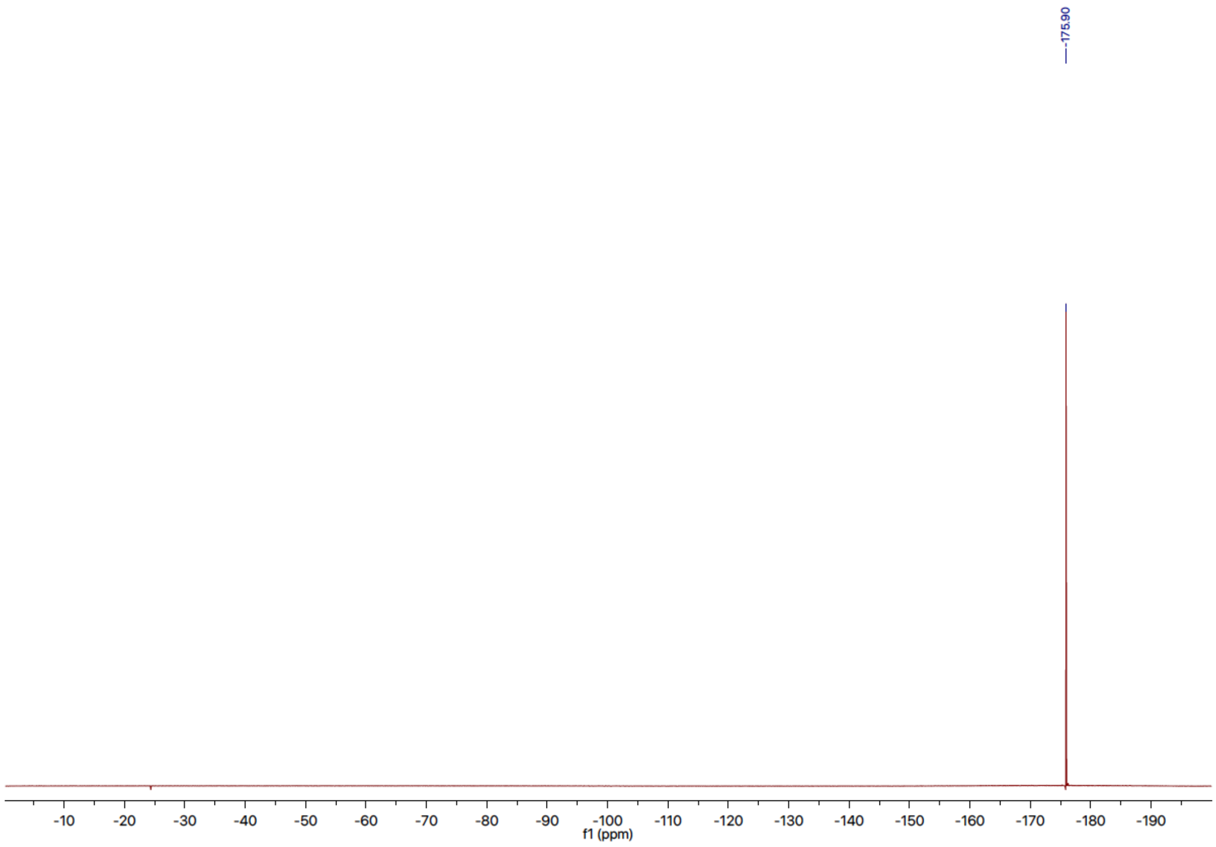

Figure S34. ^{1}H NMR spectrum of **6** (400 MHz, $CDCl_3$)

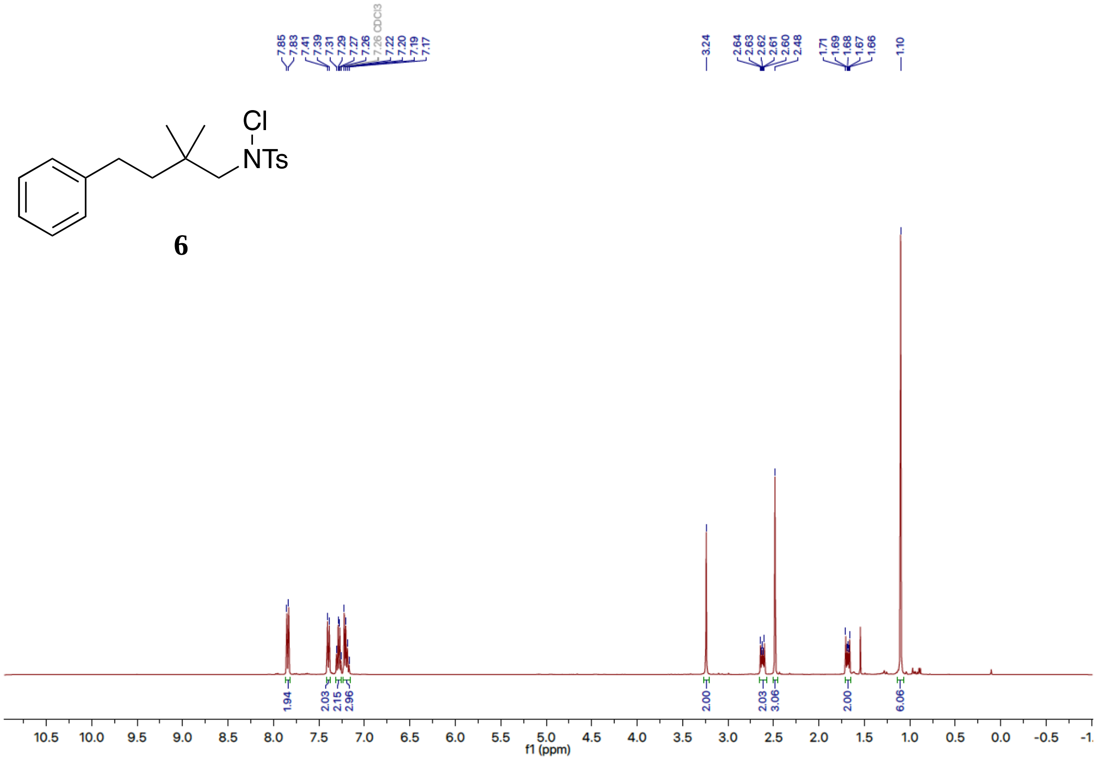

Figure S35. $^{13}C\{^{1}H\}$ NMR spectrum of **6** (100 MHz, $CDCl_3$)

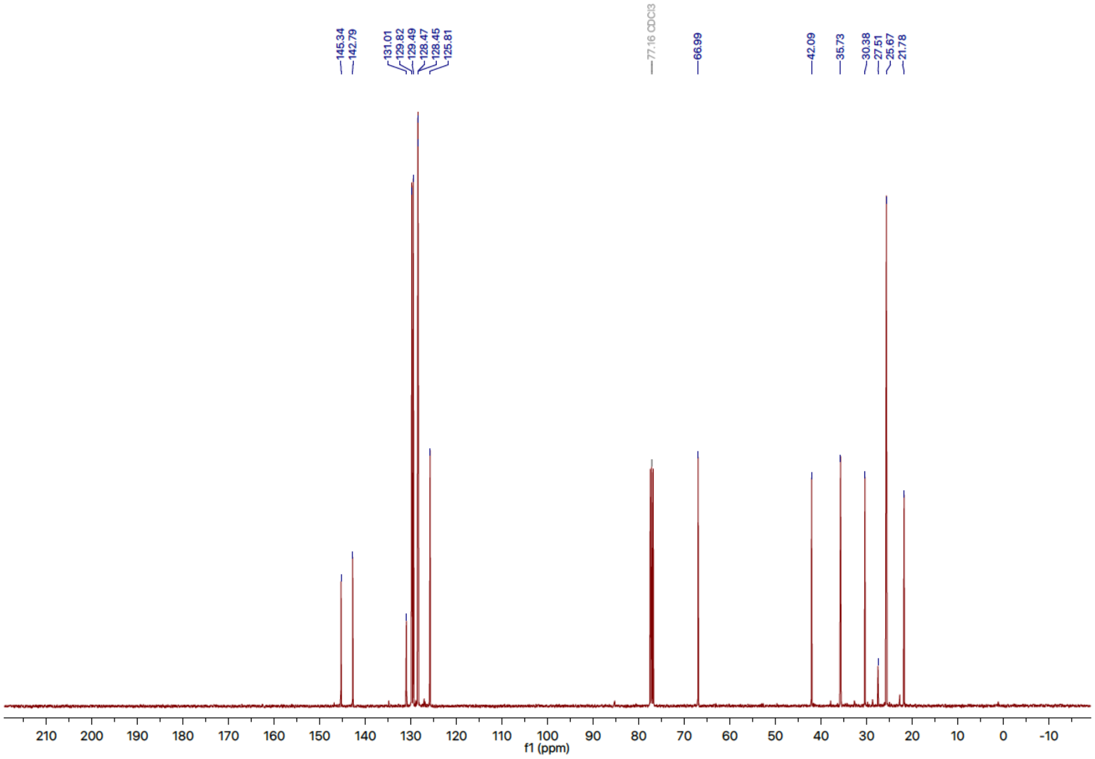

Figure S36. ^{1}H NMR spectrum of **7** (500 MHz, $CDCl_3$)

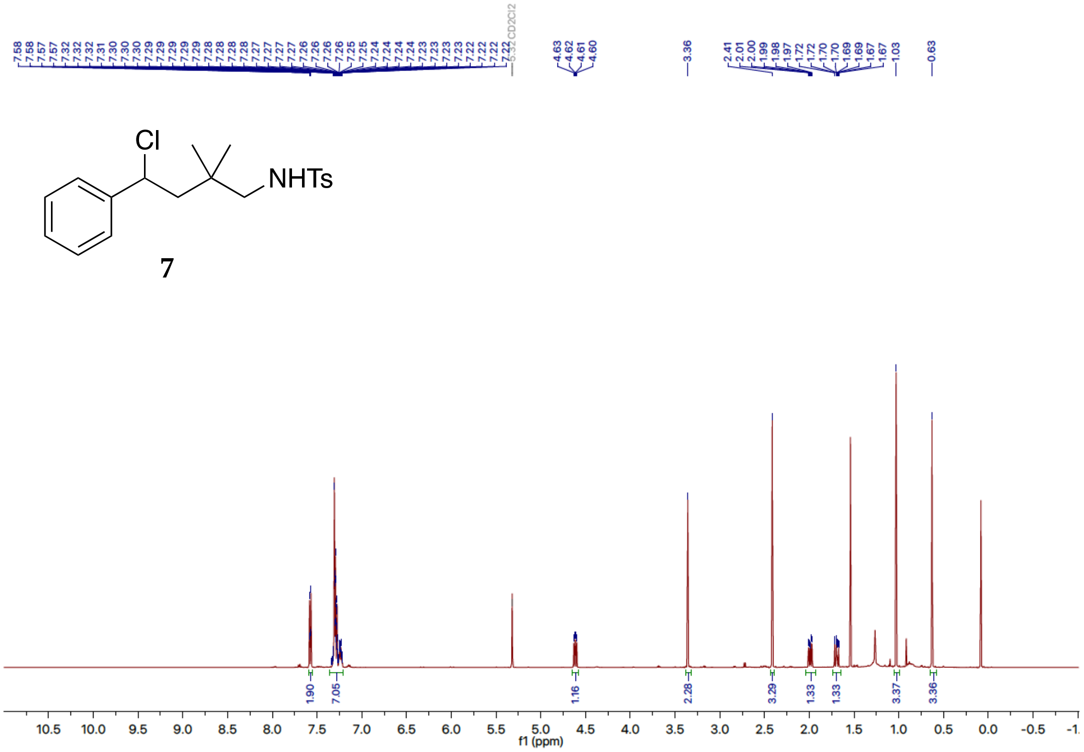

Figure S37. $^{13}C\{^{1}H\}$ NMR spectrum of **7** (125 MHz, $CDCl_3$)

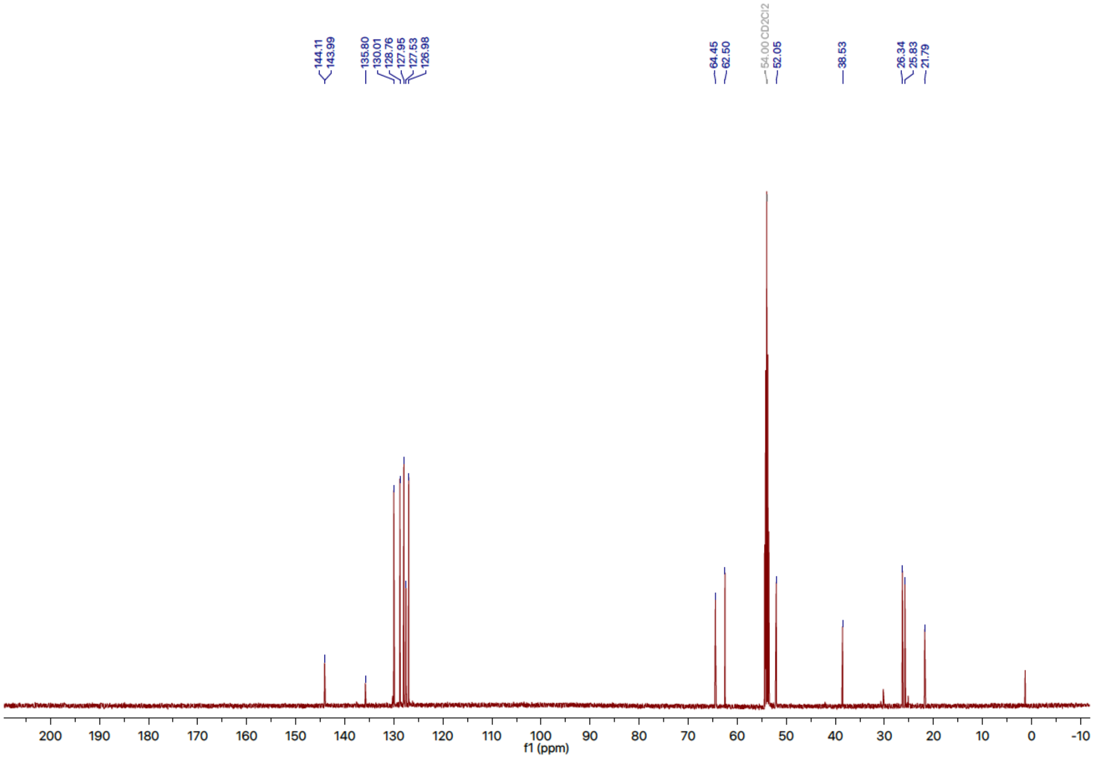

Figure S38. ^{1}H NMR spectrum of **4a** (400 MHz, $CDCl_3$)

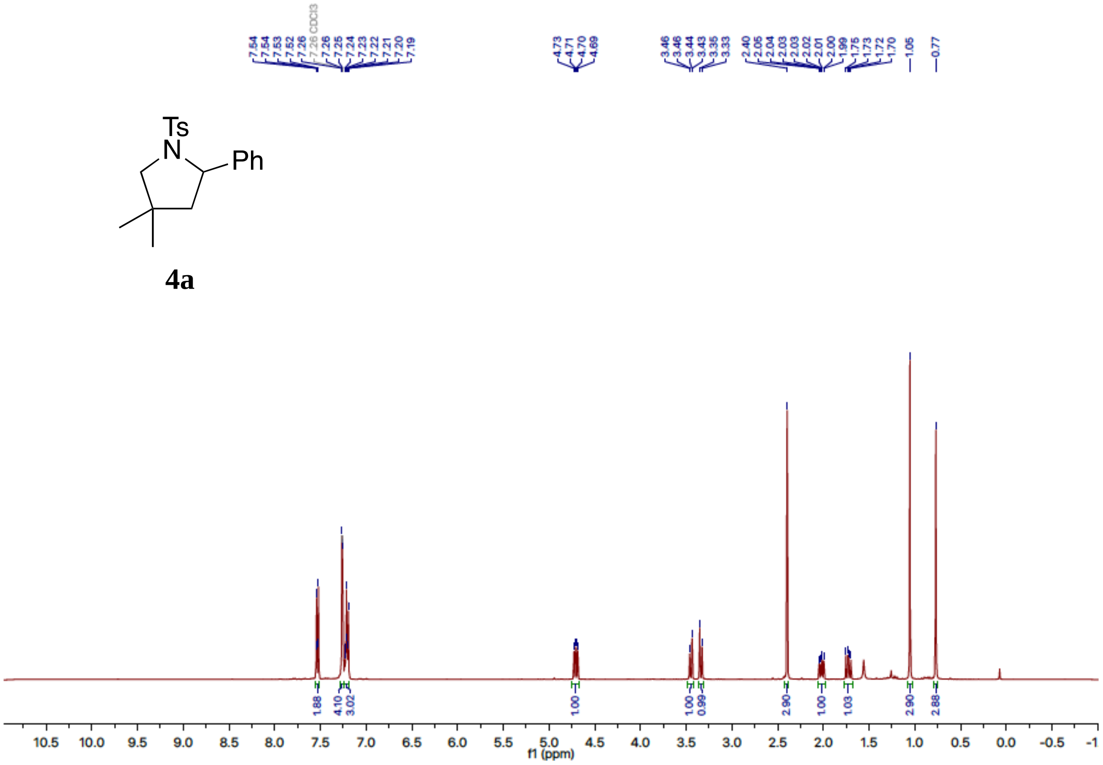

Figure S39. $^{13}C\{^{1}H\}$ NMR spectrum of **4a** (100 MHz, $CDCl_3$)

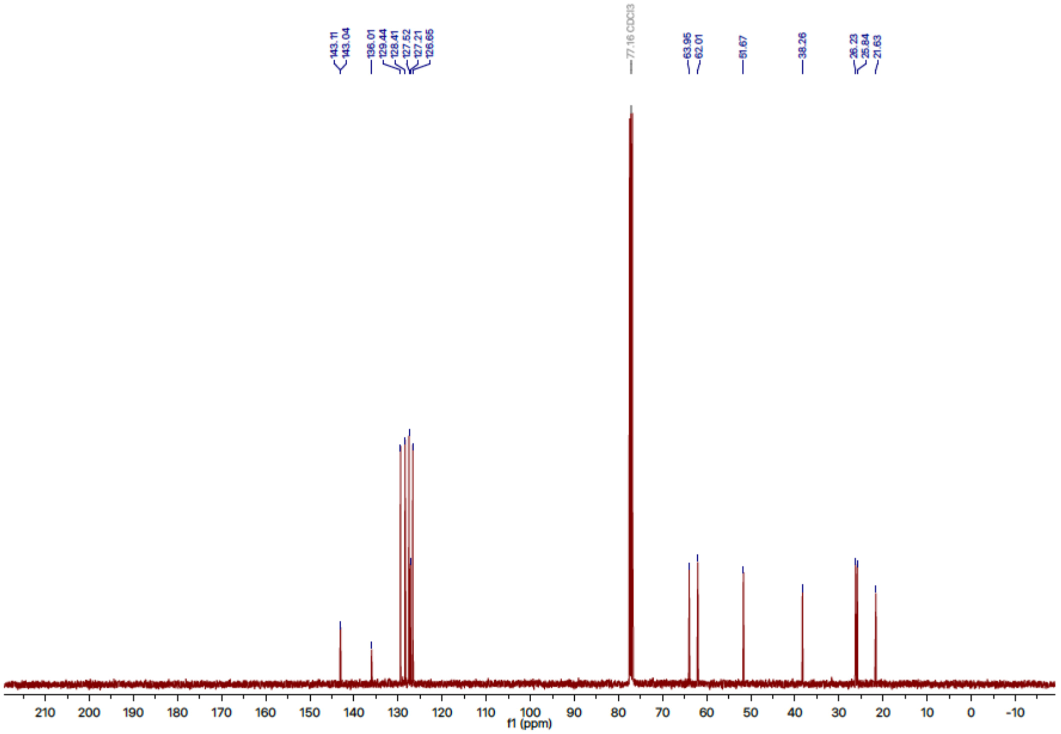

Figure S40. ^{1}H NMR spectrum of **4b** (300 MHz, $CDCl_3$)

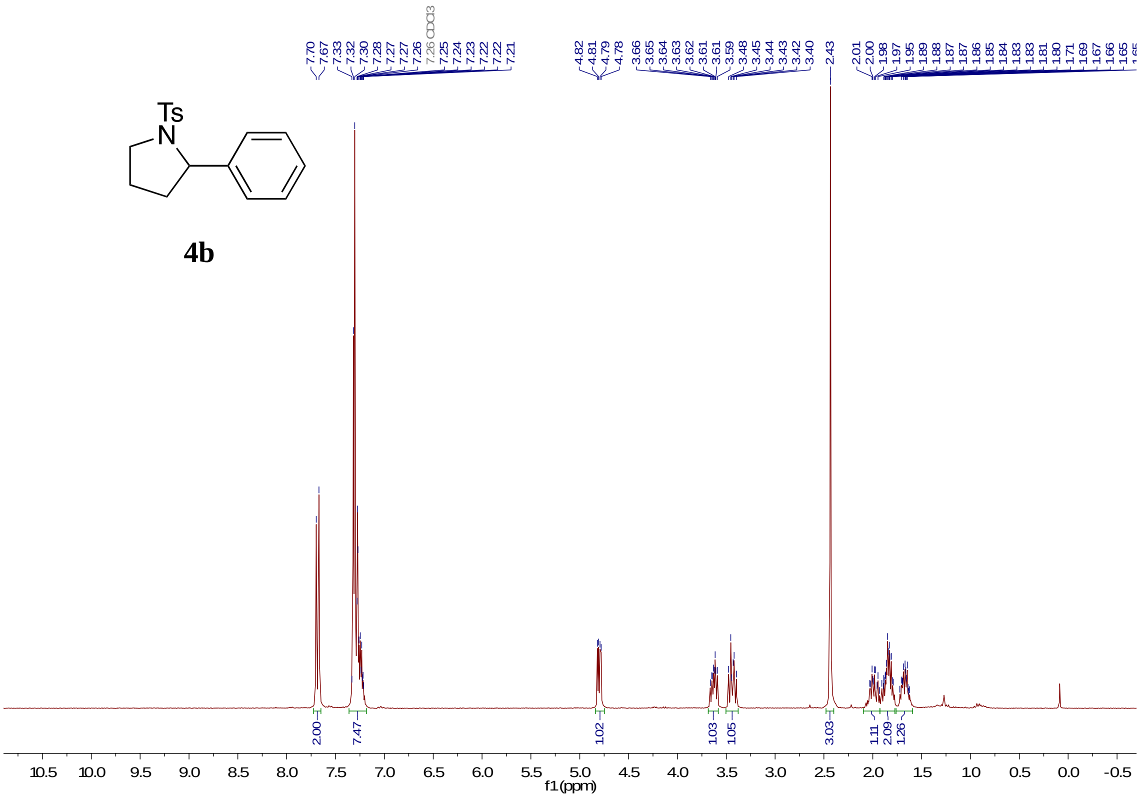

Figure S41. $^{13}C\{^{1}H\}$ NMR spectrum of **4b** (75 MHz, $CDCl_3$)

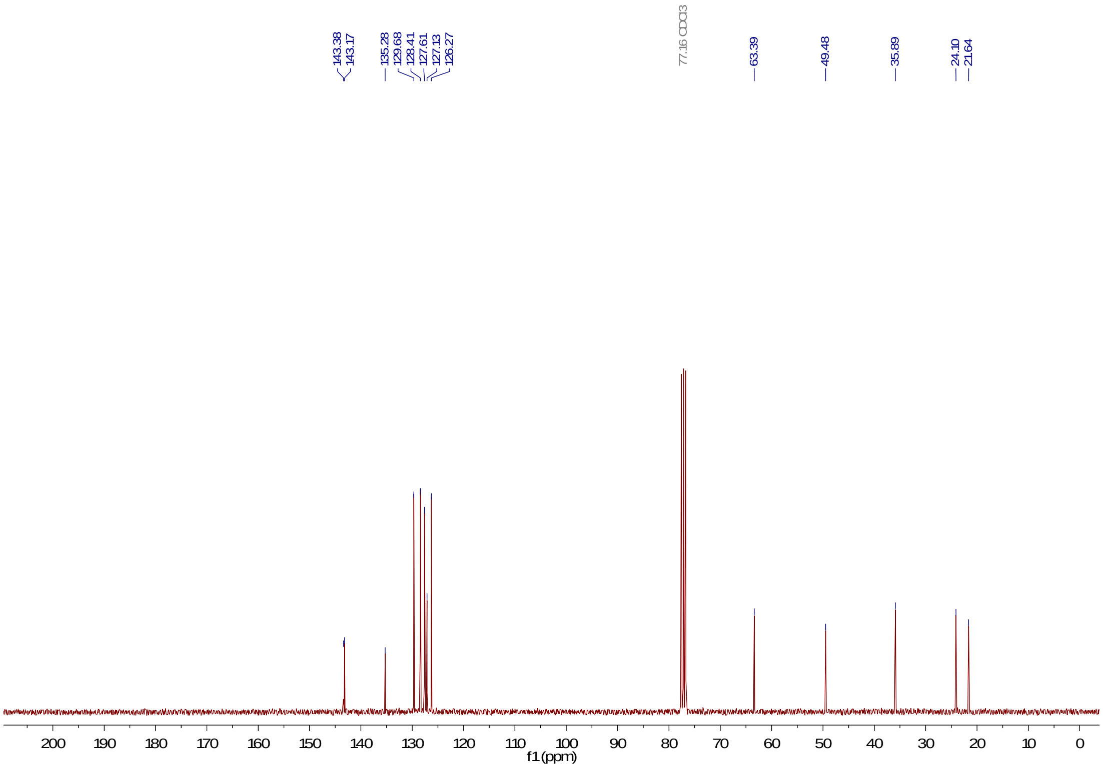

Figure S42. ^{1}H NMR spectrum of **4c** (400 MHz, $CDCl_3$)

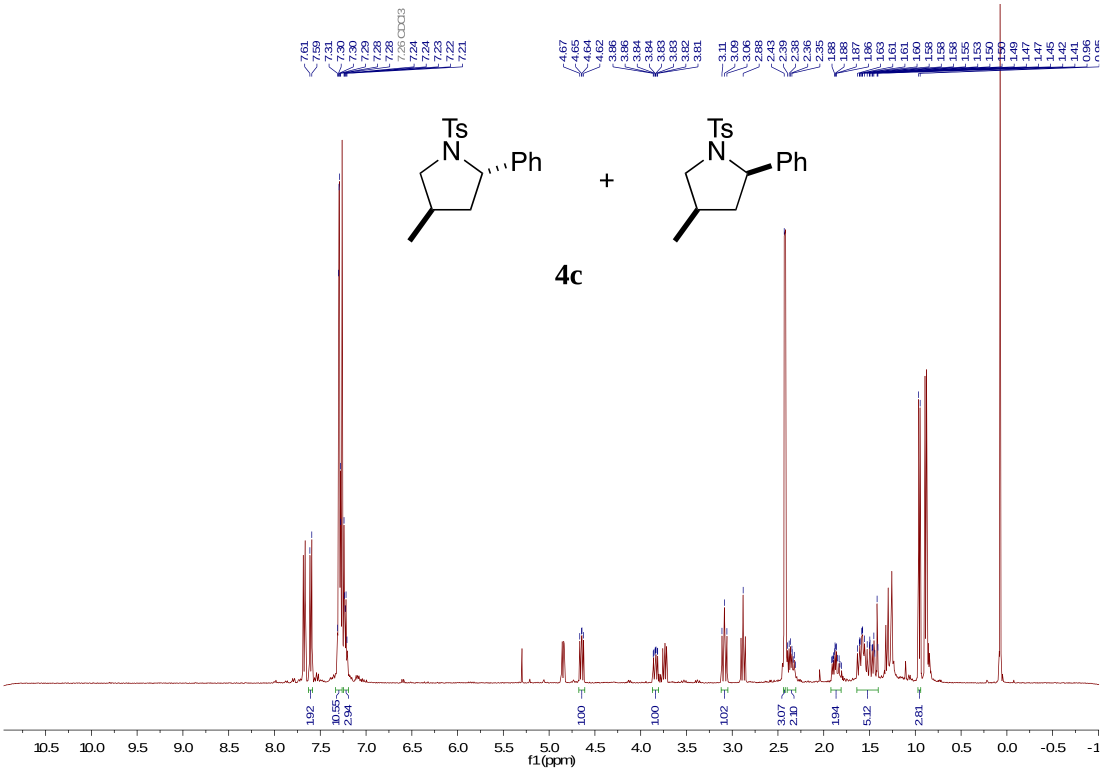

Figure S43. $^{13}C\{^{1}H\}$ NMR spectrum of **4c** (100 MHz, $CDCl_3$)

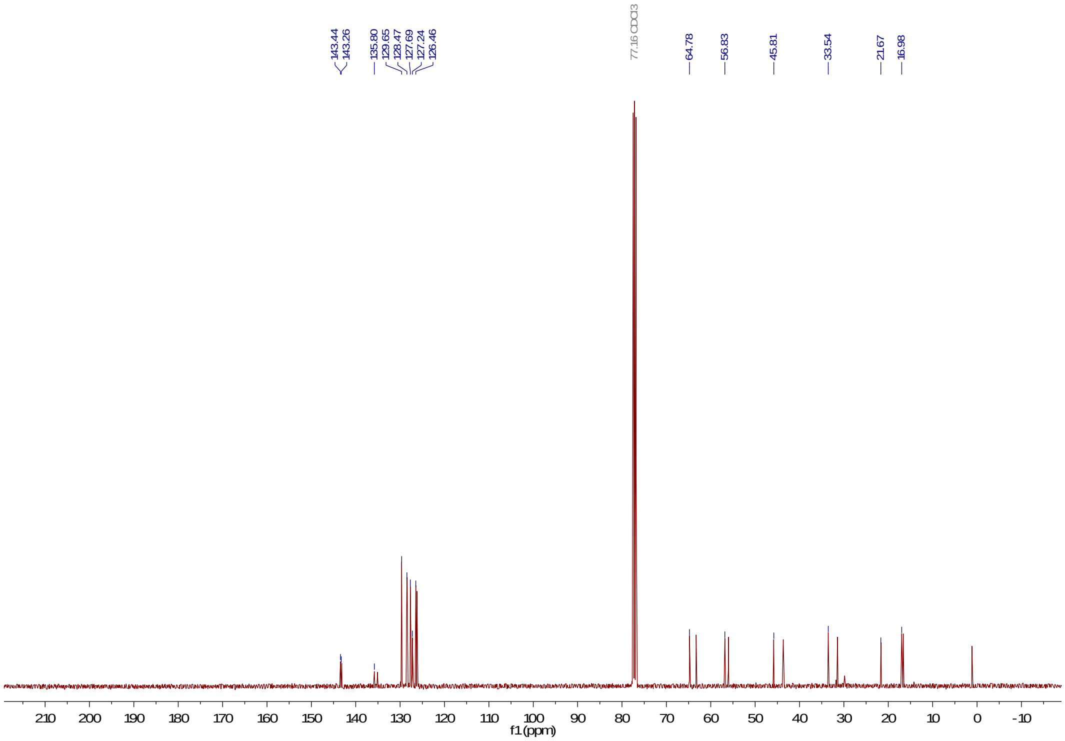

Figure S44. ^{1}H NMR spectrum of **4d** (400 MHz, $CDCl_3$)

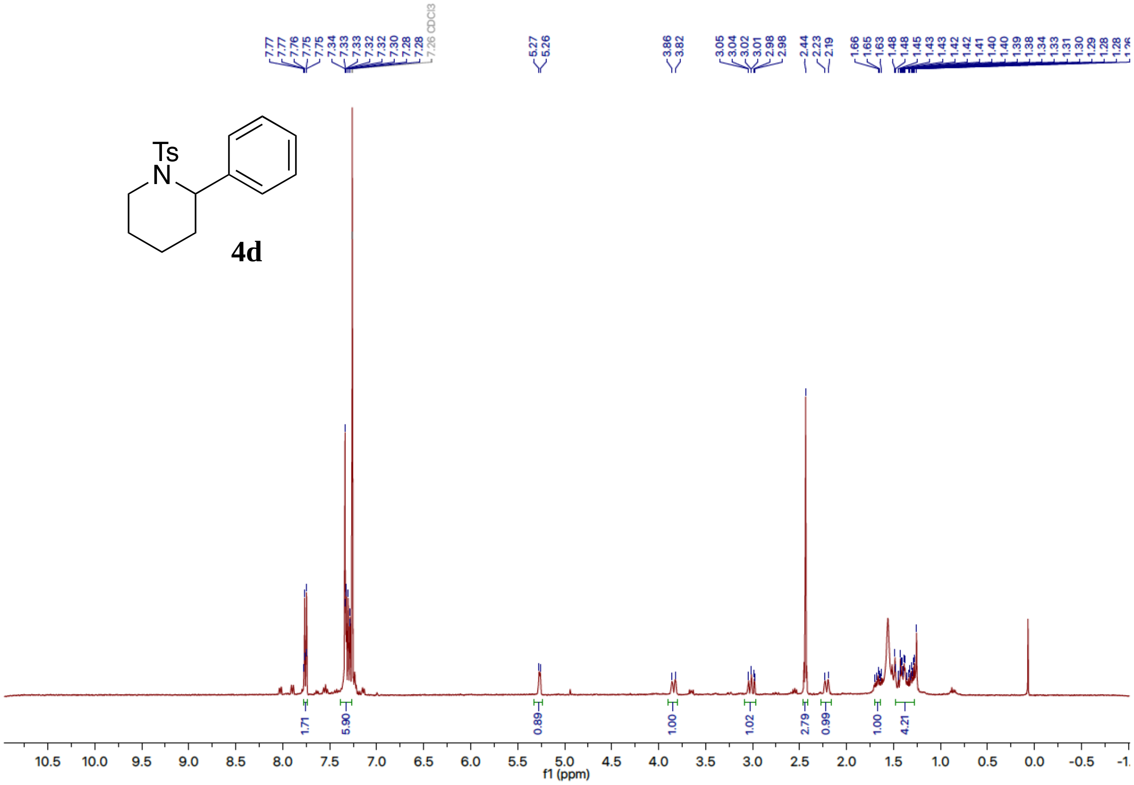

Figure S45. ^{3}C{^{1}H} NMR spectrum of **4d** (100 MHz, $CDCl_3$)

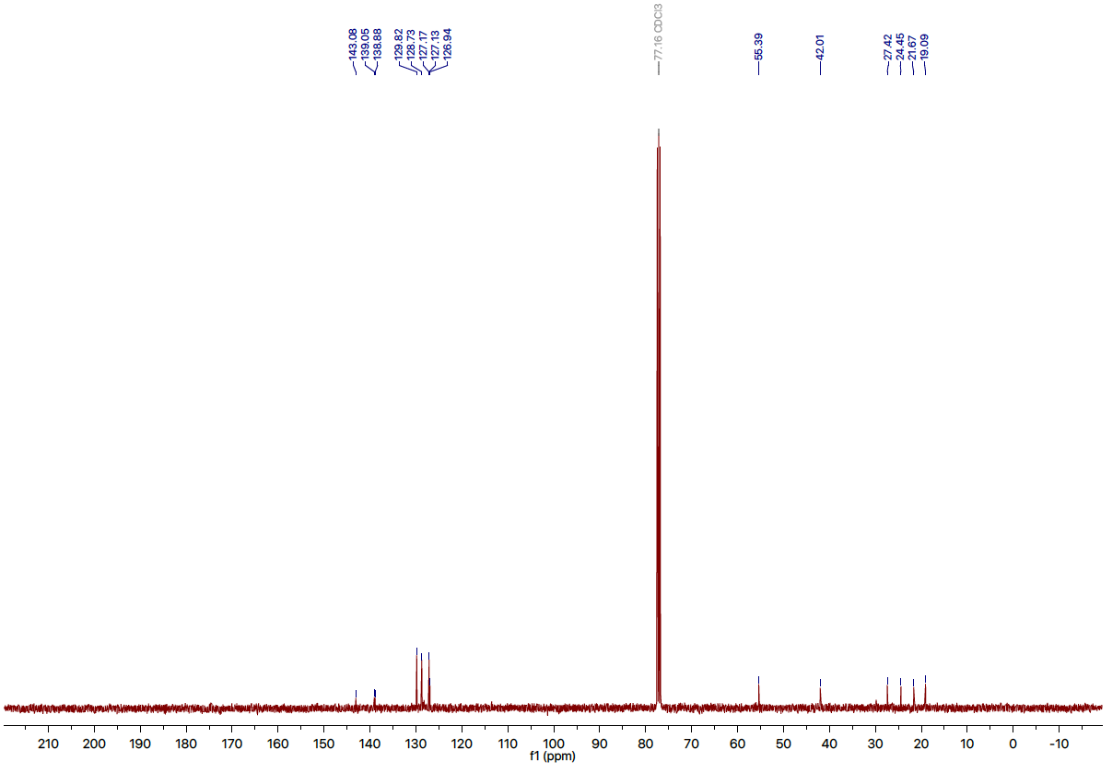

XVII. References

(1) Fujisawa, K.; Ono, T.; Ishikawa, Y.; Amir, N.; Miyashita, Y.; Okamoto, K.-I.; Lehnert, N. *Inorg. Chem.* **2006**, *45*, 1698.

(2) Caballero, A.; Díaz-Requejo, M. M.; Belderraín, T. R.; Nicasio, M. C.; Trofimenko, S.; Pérez, P. J. *J. Am. Chem. Soc.* **2003**, *125*, 1446.

(3) Mairena M. A.; Urbano, J.; Carbajo, J.; Maraver, J. J.; Alvarez, E.; Díaz-Requejo, M. M.; Pérez, P. J. *Inorg. Chem.* **2007**, *46*, 7428.

(4) Mealli, C.; Arcus, C. S.; Wilkinson, J. L.; Marks, T. J.; Ibers, J. A. *J. Am. Chem. Soc.* **1976**, *98*, 711.

(5) Álvarez, M.; Urbano, J.; Fructos, M. R.; Álvarez, E.; Pérez, P. J. *Eur. J. Inorg. Chem.* **2018**, 2026.

(6) Martínez, C.; Muñiz, K. *Angew. Chem. Int. Ed.* **2015**, *54*, 8287.

(7) Becker, P.; Duhamel, T.; Stein, C. J.; Reiher, M.; Muñiz, K. *Angew. Chem. Int. Ed.* **2017**, *56*, 8004.

(8) Elliott, L.D.; Wrigglesworth. J.W.; Cox, B.; Lloyd-Jones, G.C.; Brooker-Milburn, K.I. *Org. Lett.* **2011**, *13*, 728.

(9) Zhang, H.W.; Muñiz, K. *ACS Catal.* **2017**, *7*, 4122.

XVIII. Computational Details

All the calculations were carried out using the Gaussian09 (V D.01)[1] program package and density functional theory as the method. B3LYP-D3[2,3,4,5] has been used as the functional due to its good agreement in similar copper catalyzed reactions.[6,7] Optimizations and frequency calculations were calculated using the basis set I: 6-31G(d)[8,9,10] basis set for all atoms except for Cu, where LANL2TZ(f) and the associated pseudopotential was used.[11,12,13] Potential energies were further refined using the larger basis set (basis set II) 6-311+G(d,p)[14] for all atoms except for Cu, where we kept the same basis set. All the stationary points were characterized as minima (zero imaginary frequencies) or transition states (one imaginary frequency) using vibrational frequency analysis. Free energy corrections were applied at 298K and 1 atm. Solvation was implicitly considered in all the calculations using the SMD model and toluene as solvent ($\varepsilon = 2.3741$).[15]

All the energies reported along the manuscript are free energies in kcal/mol calculated as the sum of the potential energy with the large basis set plus the free energy correction calculated with the low basis set.

Minimum energy crossing points (MECP) between singlet and triplet states were located using the Harvey's software[16,17] and the frequencies were calculated as the average of the projected frequencies at the MECP geometry.

(1) Gaussian 09, Revision D.01, M. J. Frisch, G. W. Trucks, H. B. Schlegel, G. E. Scuseria, M. A. Robb, J. R. Cheeseman, G. Scalmani, V. Barone, G. A. Petersson, H. Nakatsuji, X. Li, M. Caricato, A. Marenich, J. Bloino, B. G. Janesko, R. Gomperts, B. Mennucci, H. P. Hratchian, J. V. Ortiz, A. F. Izmaylov, J. L. Sonnenberg, D. Williams-Young, F. Ding, F. Lipparini, F. Egidi, J. Goings, B. Peng, A. Petrone, T. Henderson, D. Ranasinghe, V. G. Zakrzewski, J. Gao, N. Rega, G. Zheng, W. Liang, M. Hada, M. Ehara, K. Toyota, R. Fukuda, J. Hasegawa, M. Ishida, T. Nakajima, Y. Honda, O. Kitao, H. Nakai, T. Vreven, K. Throssell, J. A. Montgomery, Jr., J. E. Peralta, F. Ogliaro, M. Bearpark, J. J. Heyd, E. Brothers, K. N. Kudin, V. N. Staroverov, T. Keith, R. Kobayashi, J. Normand, K. Raghavachari, A. Rendell, J. C. Burant, S. S. Iyengar, J. Tomasi, M. Cossi, J. M. Millam, M. Klene, C. Adamo, R. Cammi, J. W. Ochterski, R. L. Martin, K. Morokuma, O. Farkas, J. B. Foresman, and D. J. Fox, Gaussian, Inc., Wallingford CT, **2016**.
(2) Becke, A. D. *J. Chem. Phys.* **1993**, *98*, 5648-5652.
(3) Lee, C.; Yang, W.; Parr, R. G. *Phys. Rev. B.: Condens. Mater.* **1988**, *37*, 785-789.
(4) Stephens, P. J.; Devlin, F. J.; Chabalowski, C. F.; Frisch, M. J. *J. Phys. Chem.* **1994**, *98*, 11623-11627.
(5) Grimme, S.; Antony, J.; ehrlich, S.; Krieg, H. *J. Chem. Phys.* **2010**, *132*, 154104-154119.
(6) Rodríguez, M. R.; Beltrán, A.; Mudarra, A. L.; Álvarez, E.; Maseras, F.; Díaz-Requejo, M. M.; Pérez, P. J. *Angew. Chem. Int. Ed.* **2017**, *56*, 12842-12847.
(7) Gava, R.; Olmos, A.; Noverges, B.; Varea, T.; Funes-Ardoiz, I.; Belderrain, T. R.; Caballero, A.; Maseras, F.; Asensio, G.; Pérez, P. J. *ChemCatChem* **2015**, *7*, 3254-3260.
(8) Francl, M. M.; Pietro, W. J.; Hehre, W. J.; Binkley, J. S.; Gordon, M. S.; DeFrees, D. J.; Pople, J. A. *J. Chem. Phys.* **1982**, *77*, 3654-3665.
(9) Hariharan, P. C.; Pople, J. A. *Theoret. Chim. Acta* **1973**, *28*, 213-222.
(10) Hehre, W. J.; Ditchfield, R.; Pople, J. A. *J. Chem. Phys.* **1972**, *56*, 2257-2261.
(11) Hay, P. J.; Wadt, W. R. *J. Chem. Phys.* **1985**, *82*, 270-284.
(12) Roy, L. E.; Hay, P. J.; Martin, R. L. *J. Chem. Theor. Comput.* **2008**, *4*, 1029-1031.
(13) Ehlers, A. W.; Böhme, M.; Dapprich, S.; Gobbi, A.; Höllwarth, A.; Jonas, V.; Köhler, K. F.; Stegmann, R.; Veldkamp, A.; Frenking, G. *Chem. Phys. Lett.* **1993**, *208*, 111-114.
(14) Krishnan, R.: Binkley, J. S.; Seeger, R.; Pople, J. A. *J. Chem. Phys.* **1980**, *72*, 650-654.
(15) Marenich, A. V.; Cramer, C. J.; Truhlar, D. G. *J. Phys. Chem. B* **2009**, *113*, 6378-6396.
(16) Harvey, J. N.; Aschi, M.; Schwarz, H.; Koch, W. *Theor. Chem. Acc.* **1998**, *99*, 95-99.
(17) Poli, R.; Harvey, J. N. *Chem. Soc. Rev.* **2003**, *36*, 255-263.
